# Wafer-scale robust graphene electronics under industrial processing conditions

**DOI:** 10.1039/d6cc01037g

**Published:** 2026-07-03

**Authors:** E. P. van Geest, B. S. Can, M. Makurat, C. Maheu, H. Sezen, M. D. Barnes, D. Bijl, M. Buscema, S. Shankar, D. J. Wehenkel, R. van Rijn, J. P. Hofmann, J. M. van Ruitenbeek, G. F. Schneider

**Affiliations:** a Leiden Institute of Chemistry, Leiden University Einsteinweg 55 2333CC Leiden The Netherlands g.f.schneider@chem.leidenuniv.nl; b Leiden Institute of Physics, Leiden University, Kamerlingh Onnes Laboratory Niels Bohrweg 2 2333 CA Leiden The Netherlands ruitenbeek@physics.leidenuniv.nl; c Nantes Université, CNRS, Institut des Matériaux de Nantes Jean Rouxel IMN F-44000 Nantes France; d Surface Science Laboratory, Department of Materials and Geosciences, Technical University of Darmstadt Peter-Grünberg-Straße 4 64287 Darmstadt Germany; e Applied Nanolayers B.V. Feldmannweg 17 2628 CD Delft The Netherlands

## Abstract

When commercial grade electronic devices contain nanomaterials like graphene, it is crucial that these materials resist industrial processes and harsh environments. For environments that contain water, graphene delamination due to intercalation is a notorious problem. This renders graphene incompatible with key wafer-processing steps in the semiconductor industry. In this work, a covalent pyrene-based adhesion layer is synthesized in a facile, two-step procedure. Through π–π interactions, the adhesion of graphene to silicon wafers was maintained under harsh processing conditions, *i.e.* acidic and alkaline solutions, several organic solvents, and sonication. Moreover, devices were produced with a measurement yield up to 99.7% and reproducible device-to-device electronic performance on 4-inch silicon wafers.

## Abstract 2

When commercial grade electronic devices contain nanomaterials like graphene, it is crucial that these materials resist industrial processes and harsh environments.

## Graphical abstract

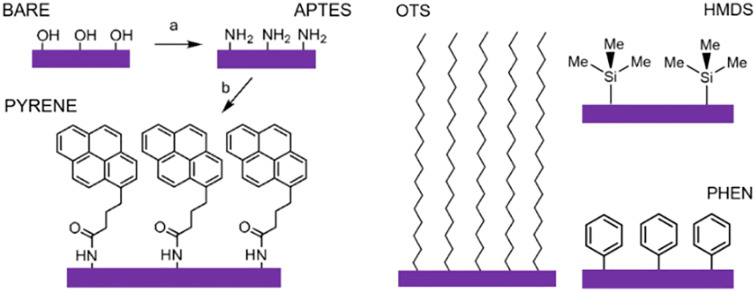

Graphene^1,2^ has been extensively explored for use in electronic devices, for example photonics and optoelectronics,^3^ micro-electromechanical systems (MEMS),^4,5^ and sensors.^6–8^ In practice, graphene on silicon wafer substrates is easily damaged by contact with chemicals and by mechanical stress. To improve stability, annealing at high temperatures can be used to increase the binding energy of graphene to SiO_2_ by two to three fold.^9–12^ Yet, graphene on hydrophilic surfaces such as SiO_2_ remains prone to water intercalation and to graphene delamination.^13^ This poses a problem, since common wafer processing steps in device fabrication require (harsh) aqueous conditions, *e.g.* acidic and alkaline conditions for etching and cleaning. The detrimental effect of such conditions can severely decrease the final device quality and yield.

An attractive approach to preventing device degradation due to water intercalation is to introduce a water-repellent adhesion layer on the silicon wafer. The SiO_2_ surface can be chemically modified through a wide range of different functionalities *via* silane chemistry, creating molecular monolayers that are highly stable and have reactive anchor groups.^14–16^ Silane-based monolayers such as hexamethyldisilazane (HMDS) and octadecyltrimethoxysilane (OTS) are commonly used in electronic devices to improve their performance.^17–19^

In this work, the hydrophobic pyrene group was covalently attached to the wafer surface *via* solution-based silane and amide coupling chemistry with commercially readily available chemicals. The pyrene moiety was attached *via* a flexible linker to facilitate optimal alignment with the graphene sheet and to promote binding. This creates a strong π–π interaction between the graphene sheet and the pyrene moieties that are, thus, covalently attached to the silicon wafer.^20,21^ Where ref. 21 quantified the enhanced adhesion force on glass resulting from surface functionalization, we compare various functionalization groups, and study the stability and electronic properties of graphene field effect transistors (GFETs) that were exposed to sonication, acidic and alkaline solutions, and organic solvents that are known to be damaging to graphene on silicon wafer. The increased adhesion leads to a drastic improvement of the stability of the GFETs under these harsh conditions, further widening the fabrication process window for graphene-based devices. Moreover, we demonstrate that the procedure could be scaled to 4-inch wafers and the modified wafers could be directly integrated in large-scale fabrication of GFETs to give a very high device yield (up to nearly 100%), with similar electronic properties as devices fabricated on bare wafers.

The surface of Si wafers having a 285 nm thermal oxide layer (or 90 nm, when stated explicitly) were covalently functionalized with a pyrene moiety (PYRENE) using a two-step process (see Fig. 1A; for details see the supplementary information (SI)). In short, clean oxygen-plasma-treated wafers were first chemically modified with a 5 vol% solution of aminopropyl–triethoxysilane (APTES) in ethanol to introduce NH_2_ groups at the wafer surface. Pyrene groups were then attached *via* an amide coupling reaction in a triethylamine (TEA)-basified DMF solution (4 drops per 10 ml DMF) containing 1-pyrenebutyric acid (15 mM) and hexafluorophosphate azabenzotriazole tetramethyl uronium (HATU, 22 mM), at room temperature. After three days, the wafers were retrieved from the solution, cleaned by sonication in acetone, followed by solvent rinses in acetone, water, and isopropanol, respectively, and dried with pressurized N_2_. Non-functionalized Si/SiO_2_ wafers (BARE), as well as wafers that were functionalized with OTS, HMDS, APTES, or phenyltriethoxysilane (PHEN) served as controls (see SI).

**Fig. 1:**
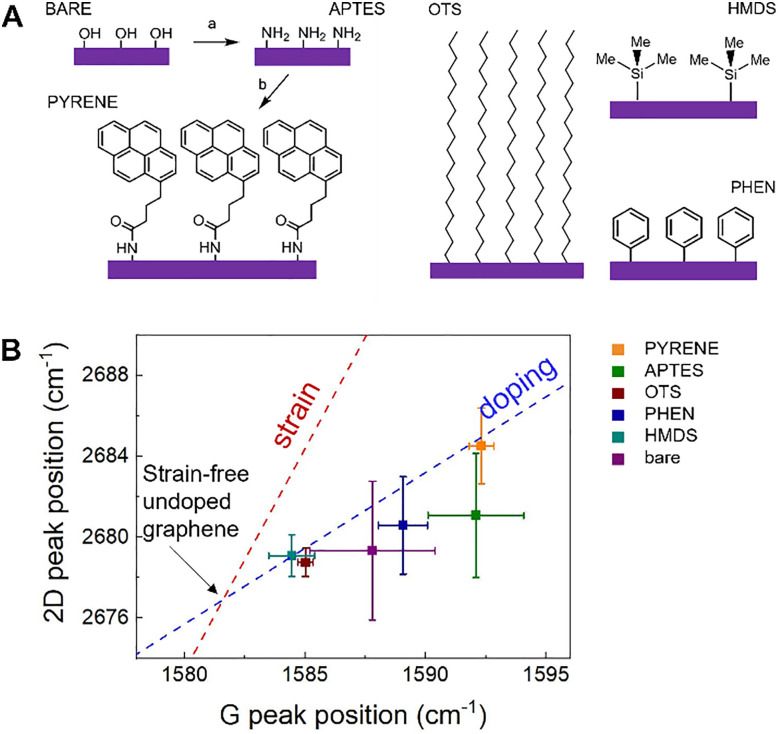
(A) Synthesis scheme for the two-step functionalization by pyrene. Reference surface modifications OTS, HMDS and PHEN are shown on the right. (B) Plot of the relative positions of the G and 2D peaks in the Raman spectra. Each data point represents four independently functionalized samples (five spectra per sample). Error bars are generated from four independently functionalized wafers. The red and blue lines indicate zero strain and zero doping axes.^22^

Increase of contact angles revealed that the hydrophobicity of the wafer increased after its chemical modification (SI, Table S1 and Fig. S1 and S2). The PYRENE wafers are more hydrophobic than HMDS-functionalized wafers, but less hydrophobic than OTS wafers. Atomic force microscopy (AFM) showed that the surface roughness of the wafers increased after the chemical modification (Table S1 and Fig. S3), as indicated by the *R*_a_ values. The RMS values are higher than for bare wafers due to large particles on the modified surfaces. X-ray photoelectron spectroscopy (XPS) confirmed the presence of nitrogen after the APTES reaction, while the C 1s emission lines increased as well (Fig. S4). For the pyrene functionalized wafers, the XPS emission lines for all the expected carbon-based bonds are identified; the C=C peak at 284.7 eV demonstrates the presence of the pyrene moieties at the surface (Fig. S4).

To study how the different surface modifications influenced the physical properties of the transferred graphene layer, Raman and liquid gating experiments were conducted. Graphene (grown on a Cu film by chemical vapor deposition) was transferred to a wafer using PMMA-assisted transfer. Raman analysis of the graphene sheets (*λ* = 532 nm, *P* = 2.3 mW) was performed after the PMMA was removed. On all graphene samples, the common peaks indicative of graphene were found (*i.e.*, the 2D, G, and occasionally the damage-indicating D peak, see Fig. S5).^23^ The 2D peak is plotted against the G peak position in Fig. 1B, which provides information on the degrees of strain and doping of graphene.^22^ For each consecutive step of the functionalization (BARE, APTES, PYRENE), the doping of graphene increases, as shown by a shift further to the right along the blue doping line. However, Raman data suggest that the graphene is less strained when it rests on the pyrene layer, as the (2D, G) point is exactly on the blue doping line, which is also the case for OTS and HMDS.

The effect of the different surface modifications on the electronic properties of liquid-gated GFETs was less obvious. GFETs were fabricated on the modified wafers (SI); through protecting the Au electrodes with a protective resin, the fabricated devices were made suitable for liquid gating (Fig. S1, S6 and S7). In such gating experiments, the presence of p-type dopants (*e.g.*, intercalated water, polymer residues, or charged surface traps) near the graphene is reflected by a positive shift of the voltage at the charge neutrality point, *V*_CNP_, of graphene. Molecular layers can reduce the effects of such doping, *e.g.* by screening the graphene from the dopant.^24–27^ They can also reduce the presence of dopants, *e.g.* by reducing water adsorption through hydrophobic layers. The reduction of doping is indeed most prominent for the highly hydrophobic layer OTS, which displays the lowest *V*_CNP_ values. For the APTES-layer *V*_CNP_ is also relatively low, which is in line with reported n-doping by the electron-donating NH_2_ groups.^28^ However, the *V*_CNP_ for the GFETs on PYRENE wafers are the same as for the unmodified surfaces, and similar *V*_CNP_ values were found for GFETs on the HMDS and PHEN functionalized wafers (Fig. S7 and Table S2). It thus seems that the pyrene layer did not have a significant effect on the doping of graphene. This appears to be in contradiction with the Raman analysis in Fig. 1B. However, the presence of an electrolyte during liquid gating modifies the environment of the graphene prohibiting direct comparison.

Another series of GFET devices was prepared to study the mechanical stability and electronic properties of graphene under mechanical stress induced by sonication in acetone. Devices on APTES and PYRENE wafers showed the highest stability towards sonication since all of them remained intact until 20 minutes of sonication. Devices on other surfaces showed device failure exceeding 25% due to loss of electrical contact between the gold electrodes (Fig. 2A and Table S2).

**Fig. 2:**
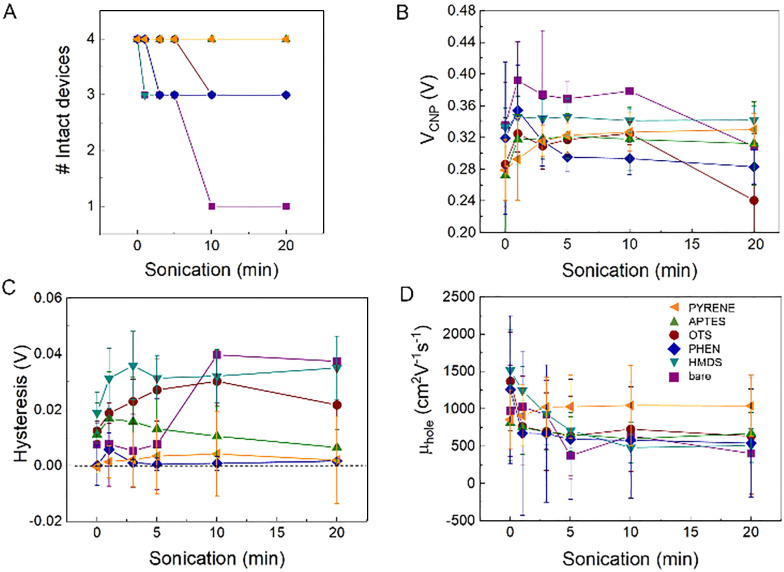
Stability of GFETs as a function of sonication time, in acetone, analysed by liquid gating. (A) Device survival. (B) Position of *V*_CNP_ in forward voltage sweeps. (C) Hysteresis in *V*_CNP_ for forward and reverse voltage sweeps. (D) Hole mobility.

We observe that sonication also affects the electronic properties of the GFETs (Fig. 2B and D). Liquid gating was performed using 0.1 M LiClO_4_ as the electrolyte. The gate voltage was swept ten times between 0 and 0.6 V at 0.01 V s^−1^, and the data of the last cycle is reported in the plots. For GFETs on APTES and pyrene-modified surfaces the resistance *R*_max_ at *V*_CNP_ did not increase significantly, while for all other surface types *R*_max_ increased already after a relatively short sonication period (<5 min, Fig. S6). The hole mobility *μ*_h_ was obtained from the gate voltage curves (Fig. 2D). In most cases, *μ*_h_ decreases by a factor of 2 or 3. However, for PYRENE it even increases.

For all surface modifications, the observed shifts in *V*_CNP_ of surviving devices are modest. The differences in the hysteresis in the positions of *V*_CNP_ for forward and backward sweeps are more pronounced. Devices with BARE, OTS, and HMDS-modified surfaces showed hysteresis directly after fabrication, which increased rapidly as they underwent sonication (Fig. 2C).

Interestingly, PHEN- and pyrene-modified devices remained practically hysteresis-free. Hysteretic behaviour indicates charge transfer or trapping, *e.g.* at the graphene/oxide interface or at defect sites in graphene. Alternatively, it can be due to slow rearrangement of polar species near the graphene, *e.g.* due to physisorbed or intercalated water.^29–31^ The small hysteresis for PYRENE is consistent with weakened interactions of the GFET with physisorbed and/or intercalated water and thus the effectiveness of the wafer coating in decreasing the adverse effects of water on graphene devices. Also, the observed stability with respect to sonication indicates that the PYRENE layer performs best for the protection of the graphene from water intercalation and mechanical damage.

Overall, the device survival, stable sheet resistance, hysteresis-free behaviour, and increased carrier mobility of the device on the pyrene-modified wafers shows that incorporation of the pyrene layer increases the robustness of GFETs under sonication, as was reported before,^21^ but also leaves the electronic properties of the devices intact.

To examine whether the wafer functionalization performs similarly at industrial scale, standard 4-inch wafers were chemically modified with the pyrene layer and coated with graphene *via* dry transfer by an industry-integrated process. We compare the results with bare and OTS-modified wafers. Contact angle and AFM inspection of the wafers prior to transfer showed that surfaces were successfully modified (Table S3). After transfer, the graphene layers were inspected with optical microscopy and Raman spectroscopy mapping (Fig. S8–S11 and Table S3 and S4). Remarkably, the effect of the surface modification was directly visible. For bare and pyrene-coated wafers, the graphene layer was intact (99% coverage for both), for OTS the sheet was badly damaged and in some places absent (coverage 94%). A wafer-scale Raman analysis revealed that graphene on bare wafer on average is clearly tensile-strained and doped. In contrast, OTS and PYRENE wafers show negligible strain and reduction in doping (Fig. 3A and Table S5), confirming our results at single-device level. Note that the doping of graphene on pyrene-coated wafers in the Raman analysis differs from the data at smaller scale, which is likely due to the differences in transfer method and graphene source materials.

**Fig. 3:**
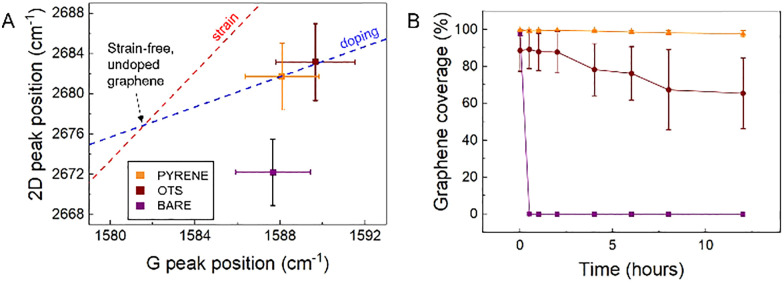
Results for full 4″ wafer-scale dry transfer of graphene on modified wafers. (A) Raman analysis of graphene on functionalized wafers. The diagonal lines represent the axes for strain and doping.^22^ (B) Graphene coverage *vs.* time for samples immersed in 0.5 M KOH, for graphene on BARE, OTS and PYRENE wafers.

Next, we evaluated whether the graphene on modified wafers survive in solutions commonly used in industrial chip processing. After dicing into 1 × 1 cm^2^ chips, these chips were immersed in either 0.5 M KOH, glacial acetic acid or *N*-methyl pyrrolidone (NMP), solvents which are commonly used for silicon etching and cleaning. Our results show that the surface modifications help to preserve the graphene quality. Graphene delaminated much slower (OTS) or hardly at all (PYRENE) as compared to the bare wafers. *E.g.*, while graphene on bare wafers was completely removed after 30 min. in the KOH solution (Fig. 3B), on PYRENE wafers the graphene was only slightly damaged after being immersed for 12 hours (98% coverage, Fig. S12 and Table S4). Similarly, for acetic acid the graphene on bare wafers was mostly removed after 4 hours, while the graphene coverage on OTS and PYRENE wafers did not change (Fig. S13). When immersed overnight in NMP, the graphene was only found to be stable on the pyrene-coated wafers; on bare SiO_2_ it was completely removed, while for OTS the graphene had mostly rolled up on itself, forming scrolls (Fig. S14). Overall, the tested surface modifications help to preserve the graphene quality under these chemical conditions, and PYRENE shows the best performance.

Finally, we have fabricated large arrays of GFETs on 4″ wafers for BARE and PYRENE, with SiO_2_ thickness of 285 and 90 nm. Finished devices were characterized *via* back-gating using an automated probe station (Fig. S15). Subsequent statistical analysis was performed on several hundreds of devices (Fig. S15–S17 and Table S6). The first performance parameter is the device measurement yield, defined as the ratio of devices successfully measured and the total number of devices, with *V*_CNP_ within the sweep range. The PYRENE wafers showed a high measurement yield of 86.2% and 99.7%, for the wafers with 285 nm and 90 nm SiO_2_ thickness respectively. Note that the definition of failure also includes devices with *V*_CNP_ outside the sweep range. These yields were significantly higher than the ones for BARE (31.4% and 89.0% for 285 and 90 nm oxide). These results imply that the adhesive layer helps to increase the device yield and the mechanical stability of the graphene during the fabrication process.

We further compared the electrical properties of the 4-terminal devices on PYRENE and BARE wafers (Table 1 and Table S6 and Fig. S15 and S16). With respect to the BARE wafers, the electronic properties appear to be preserved (p-doping and charge neutrality point). The hole and electron mobilities are similar or slightly higher on the PYRENE wafer for both 90 and 285 nm oxide layer. For GFETs produced on a wafer with 90 nm SiO_2_, the electronic characteristics were similar to the ones on 285 nm SiO_2_, although the *V*_CNP_ is lower; this is to be expected as gating is less efficient with thicker oxide, and the value found is in the range of normal values for GFETs on this type of wafers. These results confirm that the PYRENE layer can be applied directly on full wafers to obtain high device yields and preserving the device parameters.

**Table 1:** Statistical analysis for GFETS on BARE and PYRENE wafers. The hole mobility was measured in 4-terminal configuration. Standard deviation is indicated in brackets

		Devices		Mobility
Surface coating	Oxide layer (nm)	Count	Yield	*μ*_h_ × 10^3^ cm^2^ V^−1^ s^−1^
BARE	285	830	31.4	1.74 (0.11)
BARE	90	833	89.0	1.58 (0.16)
PYRENE	285	325	86.2	1.99 (0.12)
PYRENE	90	586	99.7	1.59 (0.08)

In conclusion, silicon wafers were successfully functionalized with a pyrene-based graphene adhesion layer, in a facile two-step procedure. The graphene devices fabricated on top of the modified wafers show preserved electronic properties and are more resistant to mechanical damage. Delamination of graphene in acidic and basic solutions was strongly reduced, showing that the enhanced interactions between the pyrene molecules and the graphene prevent that graphene is lifted off under these conditions. The benefits of the added pyrene layer were demonstrated on full-wafer scale, with a device yield of close to 100%, while maintaining similar electronic properties. This ultimately shows that modified wafers with the adhesive layer can be directly integrated in graphene device fabrication on the industrial scale.^32^ These will benefit from the increased stability under harsh conditions which promotes further opportunities, for example in sensors where graphene needs to be further functionalized and regenerated.

## Author contributions

E. P. van Geest designed and performed the functionalization of wafers, performed the gating experiments and overviewed the project; B. S. Can and M. Makurat contributed to the functionalization, characterization using Raman spectroscopy, and discussions; M. Makurat performed the AFM measurements; C. Maheu and H. Sezen performed the XPS measurements and participated to the related investigations and discussions; M. D. Barnes, D. Bijl, M. Buscema, S. Shankar, D. J. Wehenkel, and R. van Rijn transferred graphene on 4″ wafers, fabricated graphene field effect transistors on the wafer scale experiments and characterized/analysed the data; J. P. Hofmann, J. M. van Ruitenbeek, and G. F. Schneider supervised the work. E. P. van Geest, J. M. van Ruitenbeek and G. F. Schneider wrote the manuscript.

## Conflicts of interest

There are no conflicts to declare.

## Supplementary Material

CC-062-D6CC01037G-s001

CC-062-D6CC01037G-s002

CC-062-D6CC01037G-s003

## Data Availability

The data supporting this article have been included as part of the supplementary information (SI) as .docx, xlsx and .pptx files and are available from the article landing page. The files are presenting the raw data (see file: SupportingInformation_vanGeest_et_al-Wafer-scale_2026.docx, Datacollection-vanGeest_et_al-Wafer-scale_2026.xlsx and Datacollection_vanGeest_et_al-Wafer-scale_2026.pptx). The supplementary information file includes: materials, methods, characterization and extended data sets. See DOI: https://doi.org/10.1039/d6cc01037g.
