# Supplementary material for: Wafer-scale robust graphene electronics under industrial processing conditions
Source: Chem Commun (Camb). 2026 Jul 3;62(57):14225–9. doi: 10.1039/d6cc01037g (PMC13330647; doi:10.1039/d6cc01037g)
Supplement: CC-062-D6CC01037G-s002 [file CC-062-D6CC01037G-s002.pdf]

Supplementary Information (SI) for Chemical Communications.
This journal is © The Royal Society of Chemistry 2026

4” wafers
Optical microscope inspection after carrier removal

Graphene with PMMA polymer

Bare

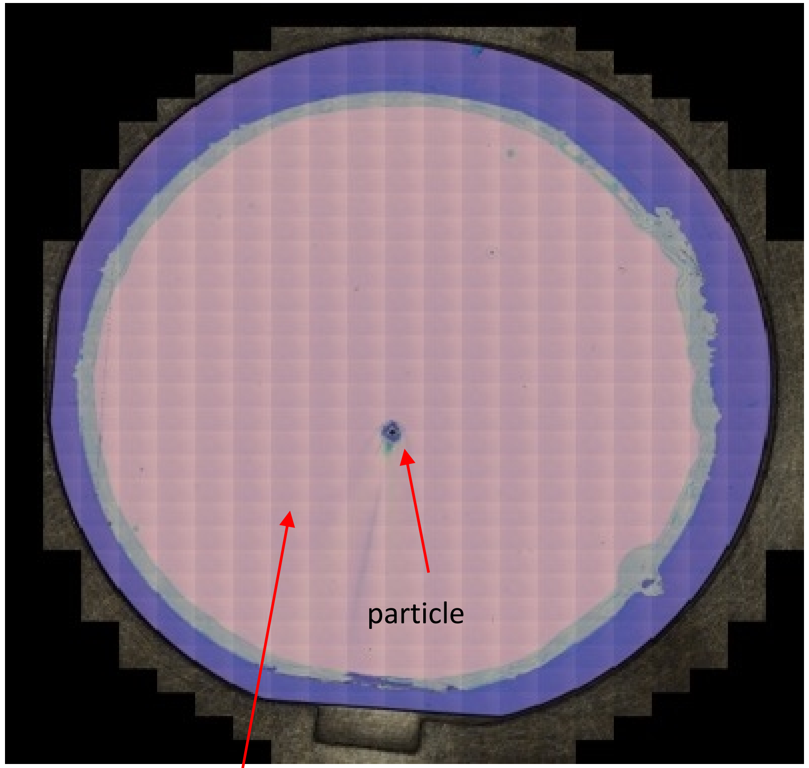

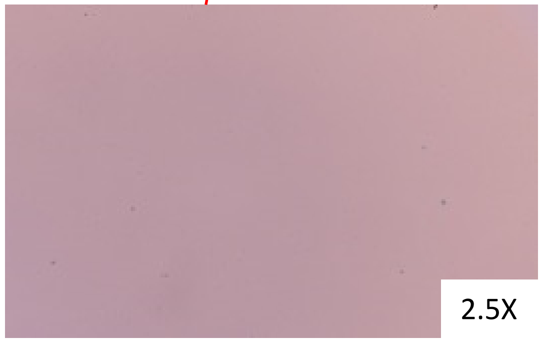

OTS

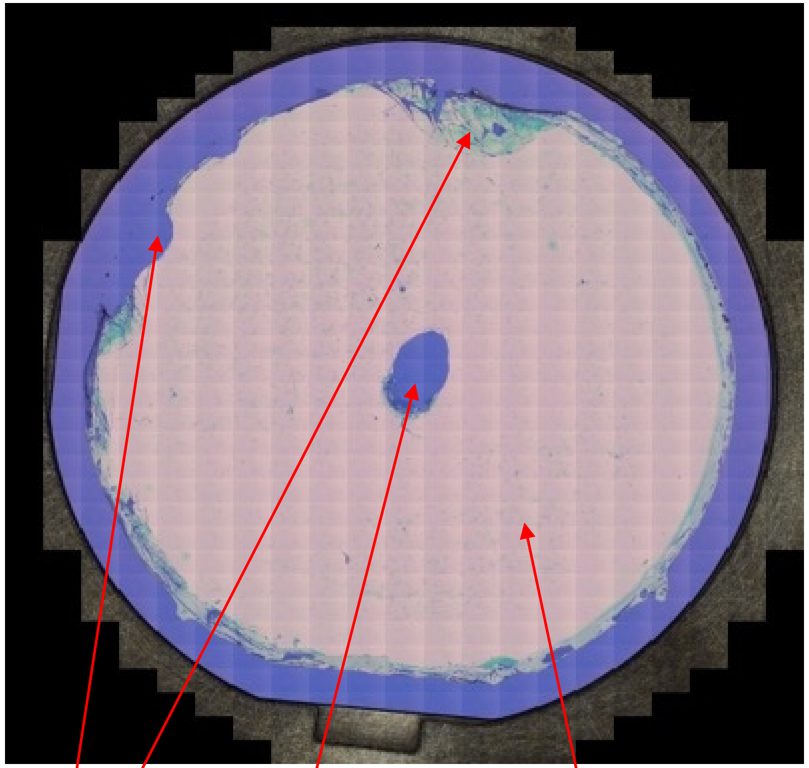

Polymer and graphene delaminated during carrier removal ➔ poor adhesion between graphene and substrate

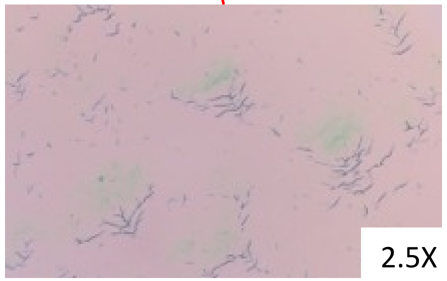

Folds in the polymer due to poor adhesion between graphene and the substrate.

Pyrene

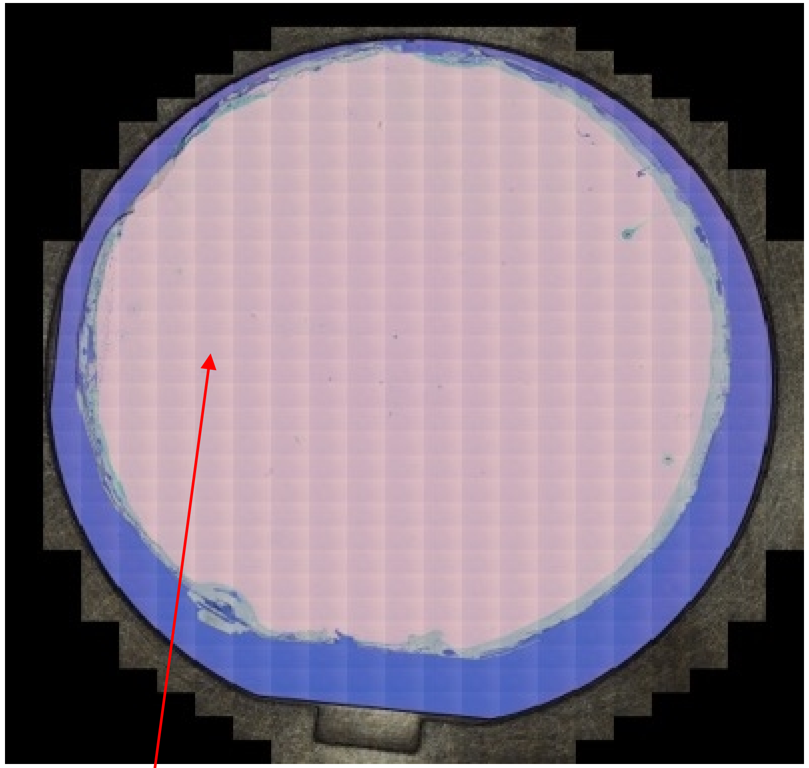

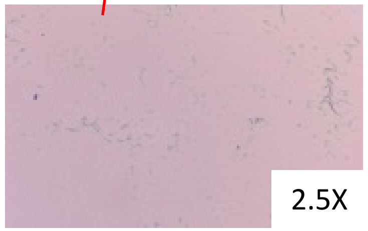

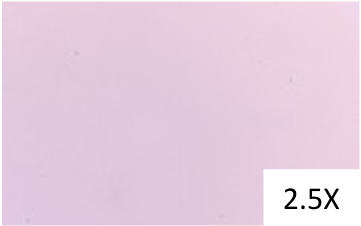

Areas with and without folds
➔ Varying adhesion across the wafer

Graphene with PMMA polymer

Bare | OTS | Pyrene

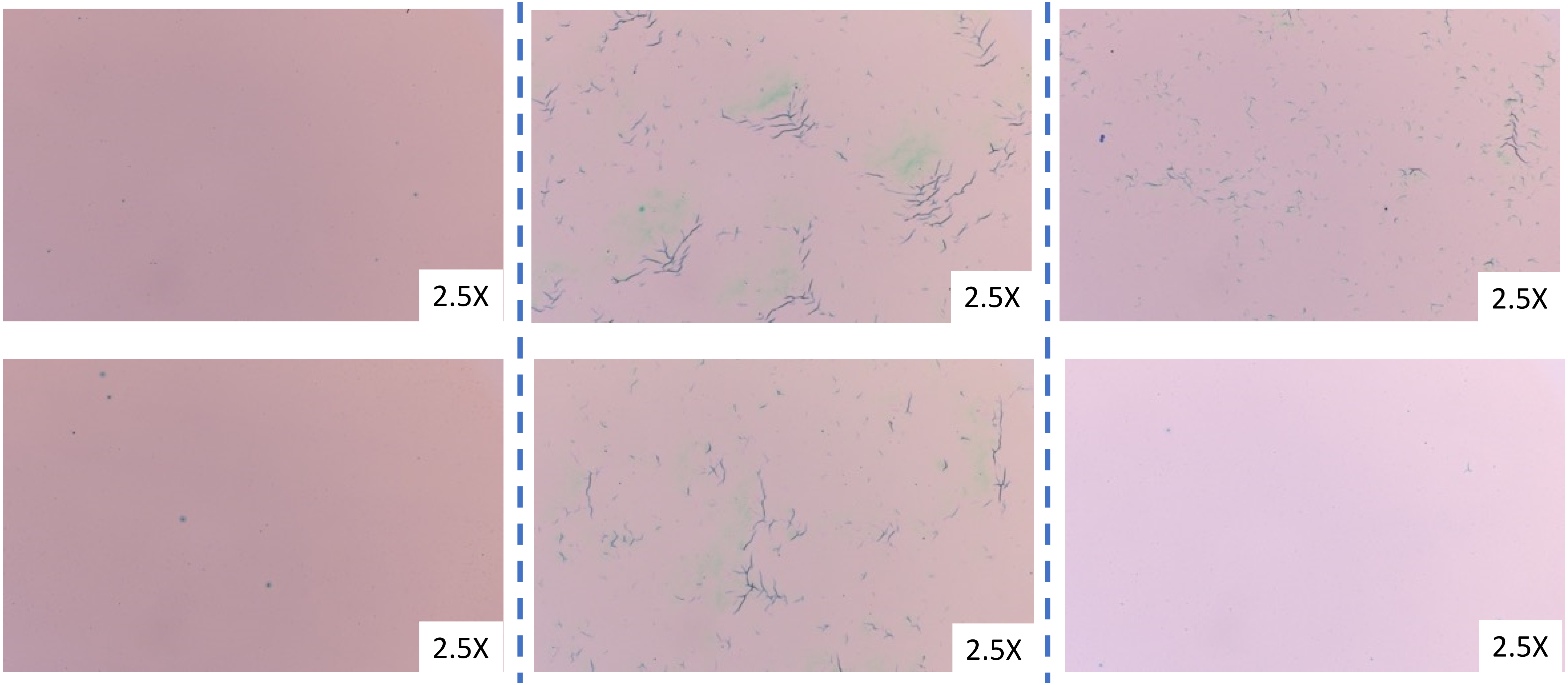

Well laminated , some particles can be observed

A lots of folds are observed in the polymer due to poor adhesion between graphene and the substrate

Graphene with PMMA polymer

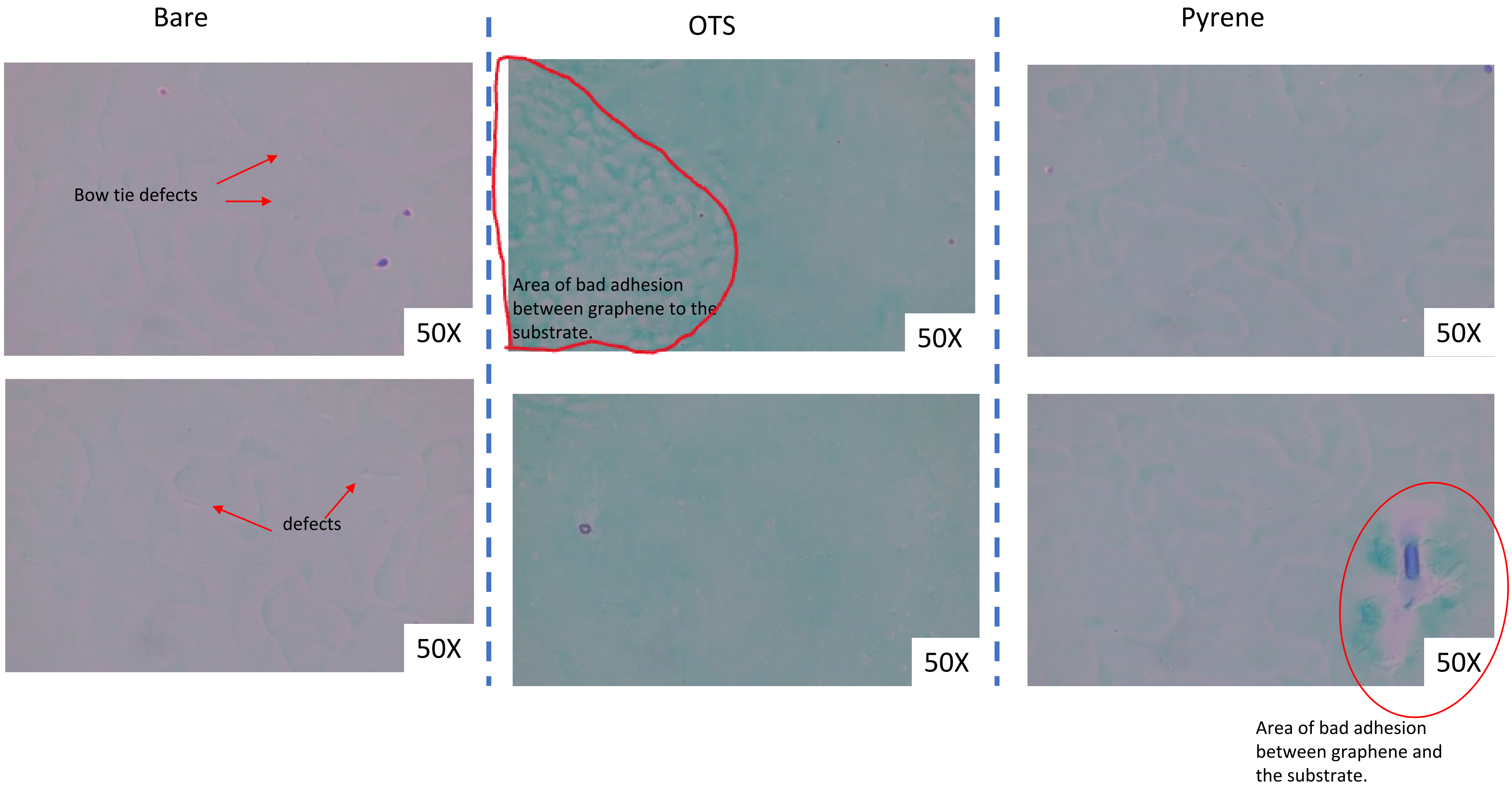

Optical microscope inspection after polymer removal

Polymer removed

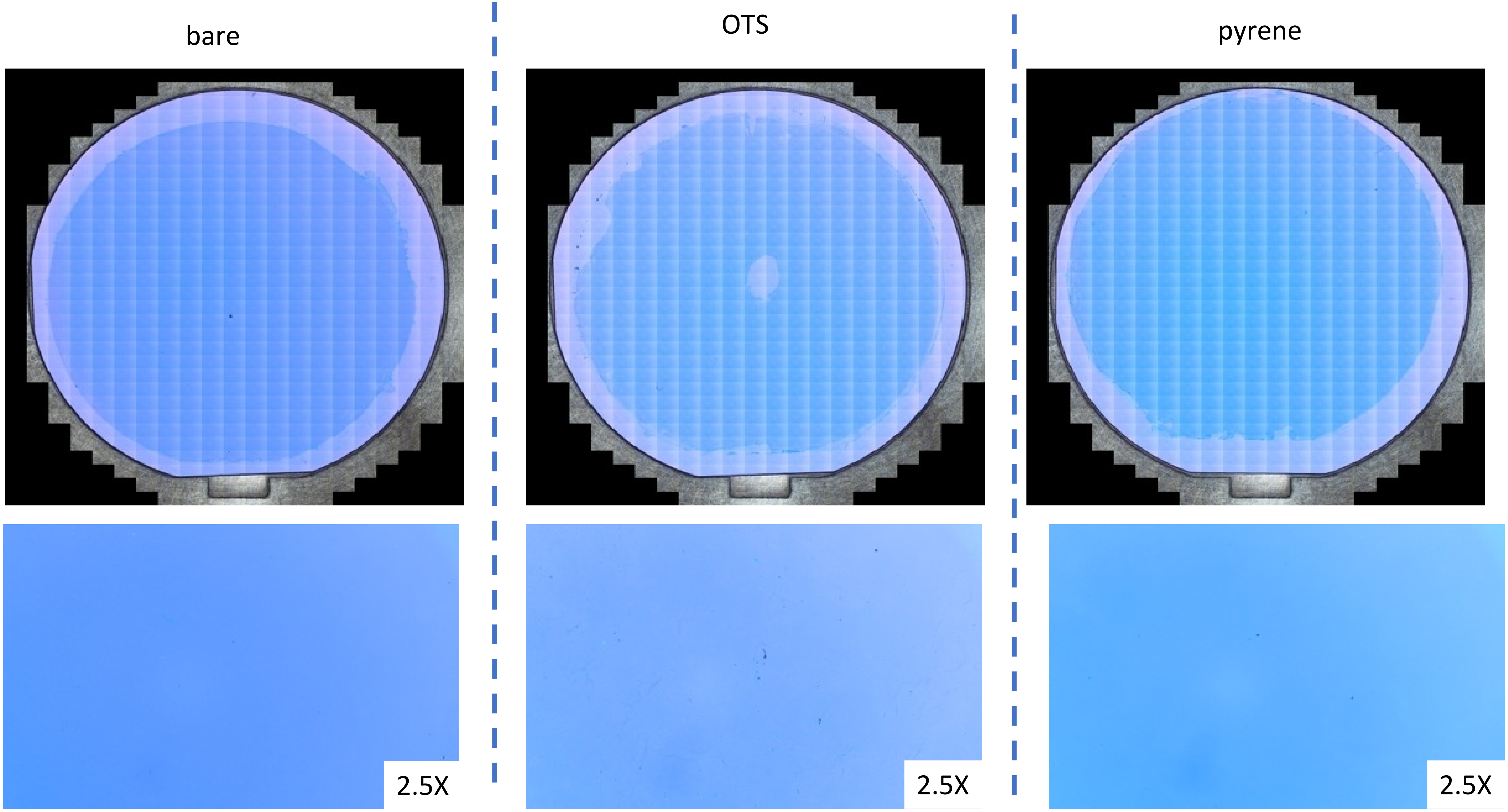

Polymer removed

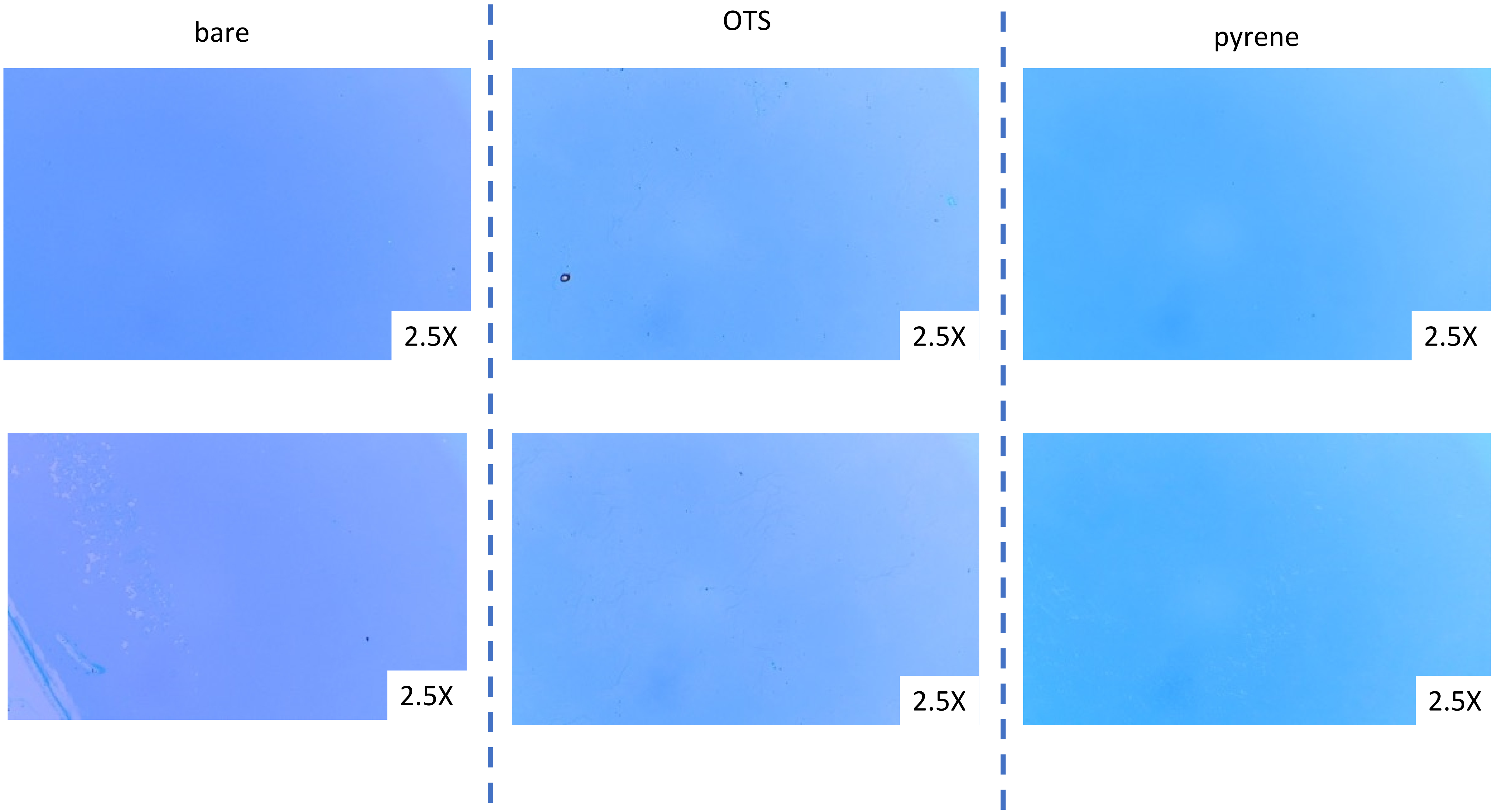

Polymer removed

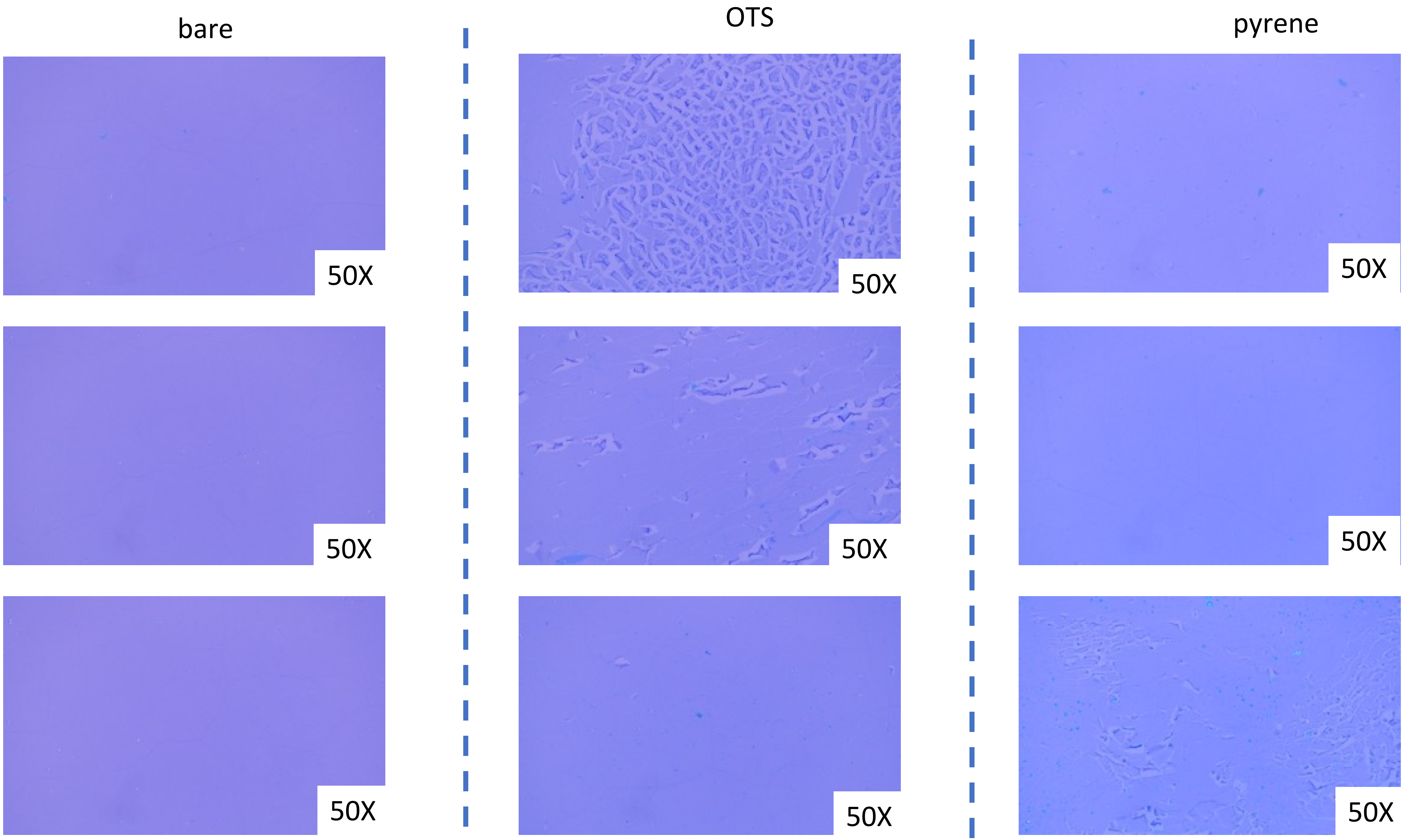

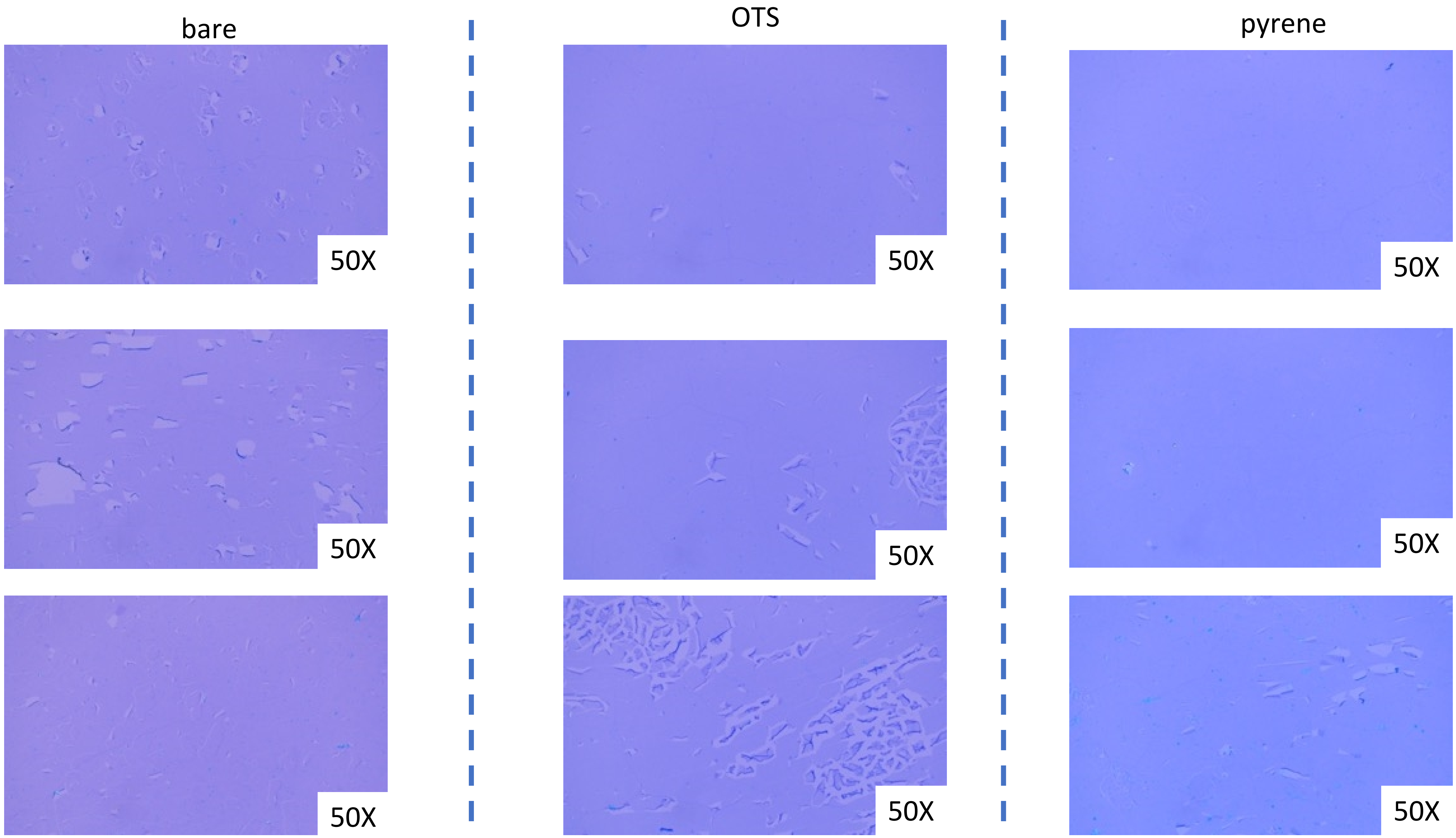
bare
OTS
pyrene
50X
50X
50X
50X
50X
50X
50X
50X
50X

Brightness and contrast enhanced image

bare

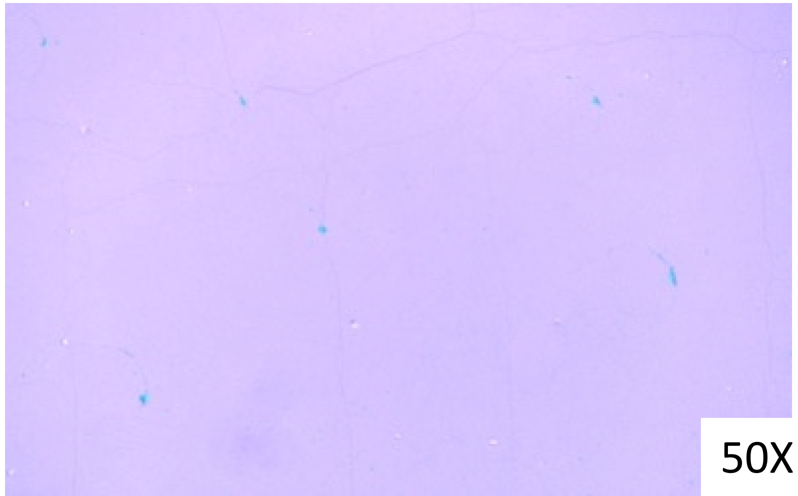

OTS

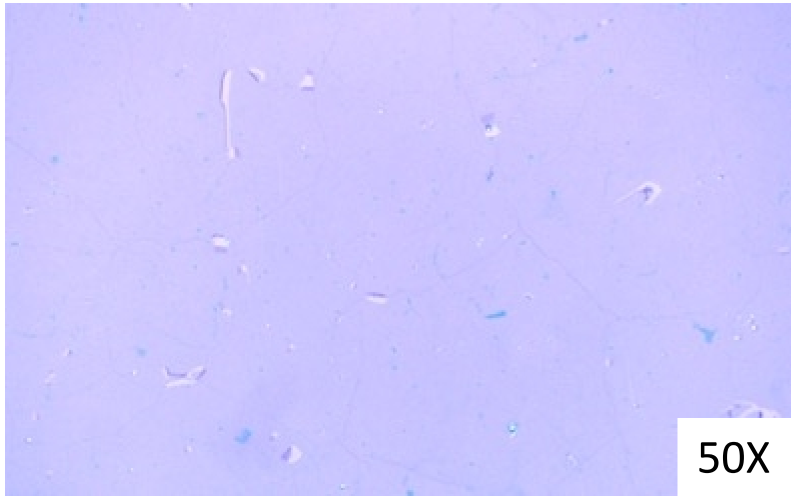

pyrene

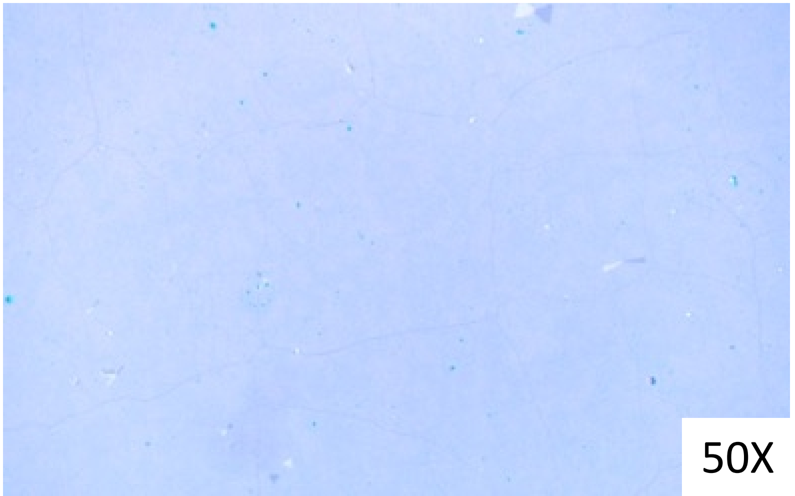

Faint patches can be observed for the pyrene sample

Raman scans on graphene on bare wafer

3 random spectra

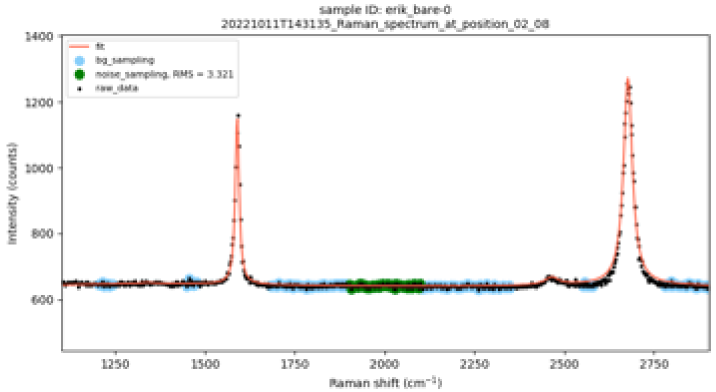

	FWHM (cm^{-1})	Pos (cm^{-1})	Int (counts)	R^2
Si	5.026	520.003	15505.353	0.9341
D	40.0	1339.164	3.796	0.99997
G	12.94	1589.123	510.515	0.99935
D+D''	44.984	2464.871	23.371	0.99994
2D	29.594	2677.802	628.4	0.9988

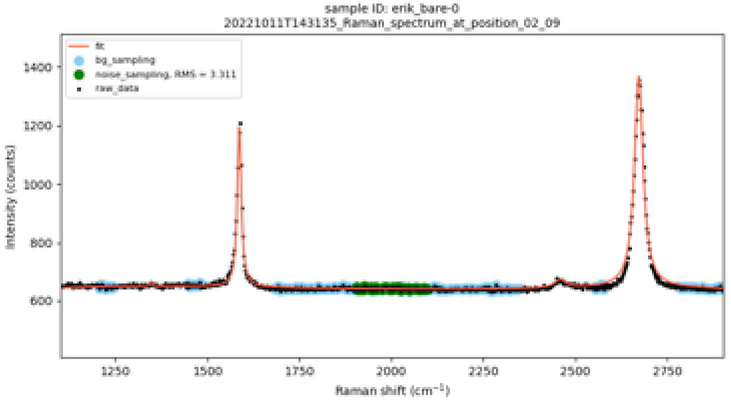

	FWHM (cm^{-1})	Pos (cm^{-1})	Int (counts)	R^2
Si	5.102	520.002	14965.02	0.92635
D	7.614	1347.248	11.856	0.99997
G	12.828	1586.081	554.467	0.99887
D+D''	35.232	2459.178	28.214	0.99993
2D	28.336	2672.642	722.716	0.99853

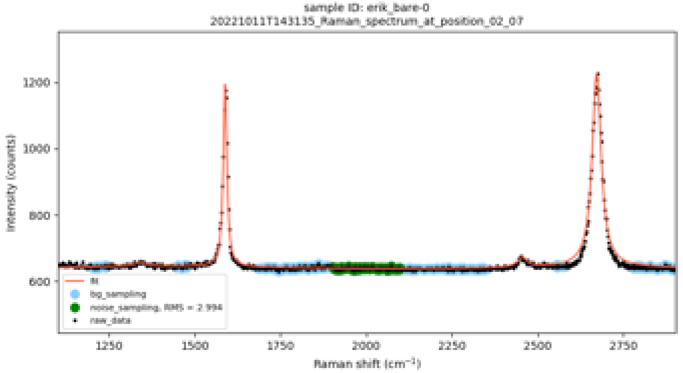

	FWHM (cm^{-1})	Pos (cm^{-1})	Int (counts)	R^2
Si	5.05	520.002	15359.828	0.92916
D	39.144	1340.252	11.622	0.99997
G	12.918	1587.828	551.088	0.9994
D+D''	33.768	2455.624	28.879	0.99994
2D	31.598	2672.672	585.548	0.99883

Summary of the Raman fits

Parameter	Value (mean)	Standard Deviation
Total Raman scans in measurement	276	N/A
Number of scans showing graphene	274	N/A
Percentage of scans with graphene	99 %	N/A
D position	1343.36	5.49
D intensity	50.34	669.09
D FWHM	25.5	10.16
G position	1587.66	1.77
G intensity	622.78	137.64
G FWHM	11.75	1.6
2D position	2672.21	3.28
2D intensity	638.74	66.85
2D FWHM	30.45	1.56
G/D Ratio	77.1	37.53

erik_bare-0: G & 2D Positions

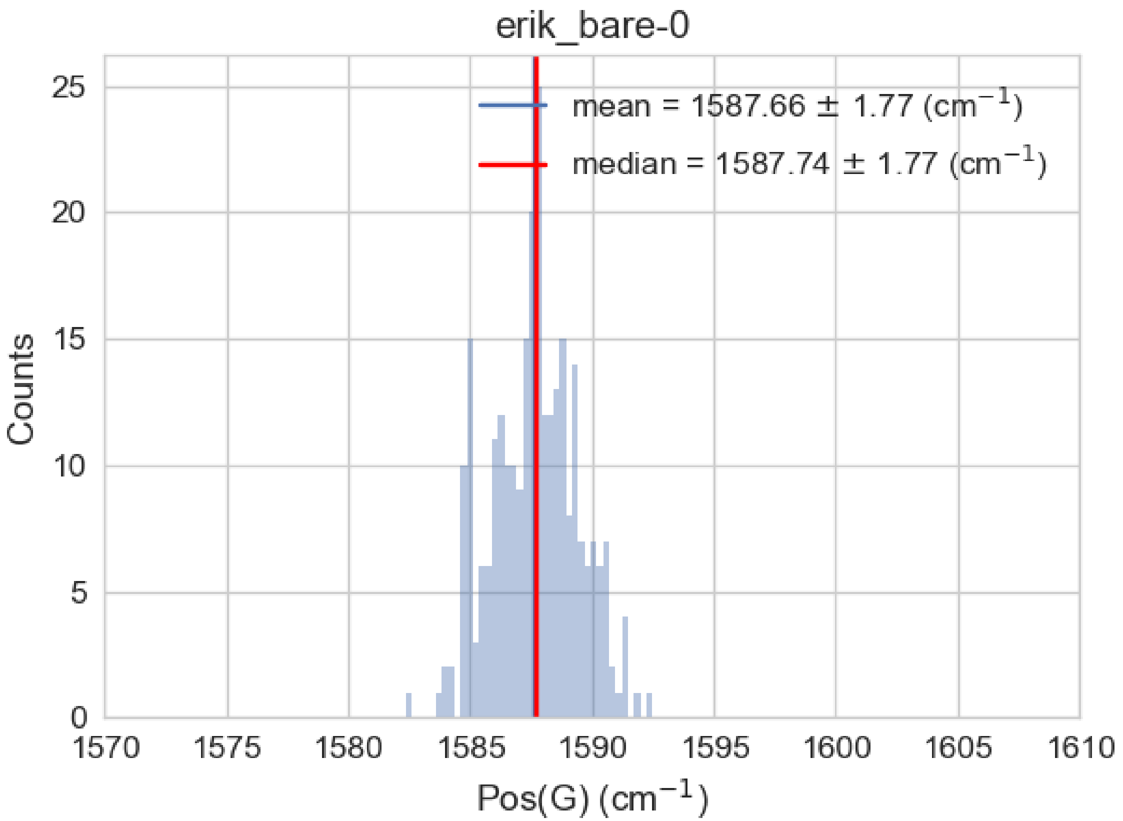

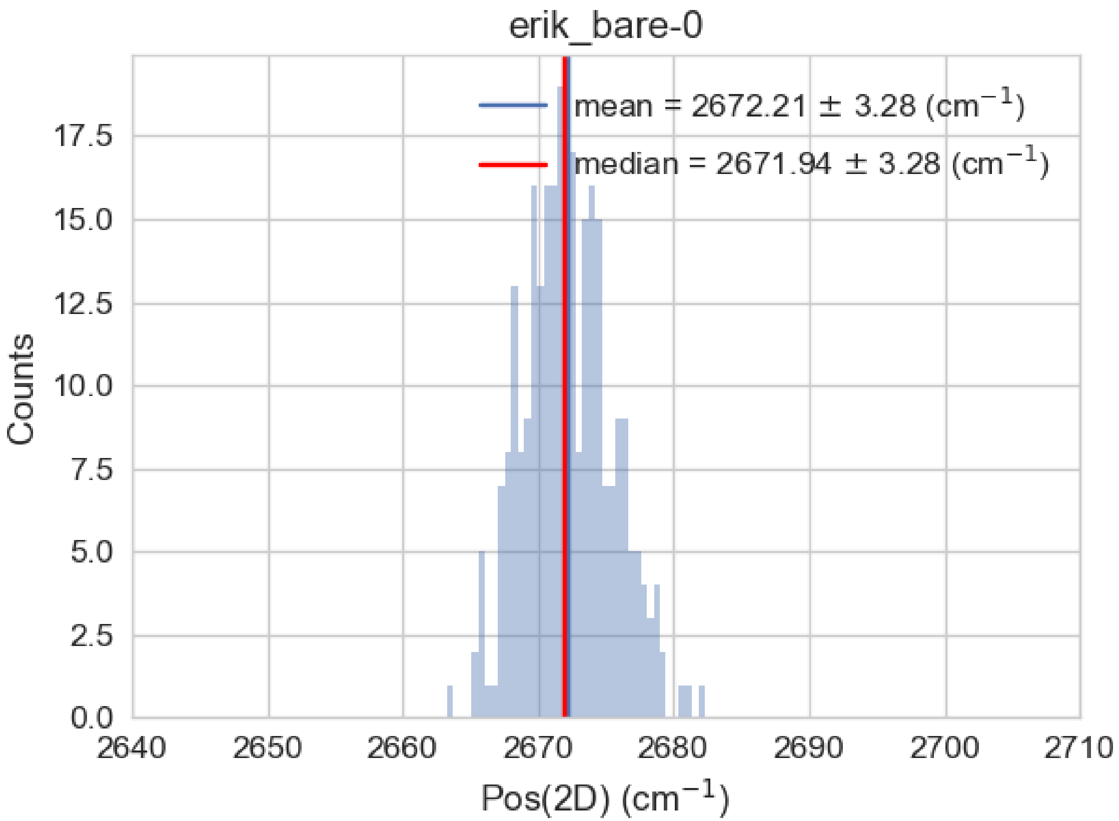

erik_bare-0: G & 2D FWHM

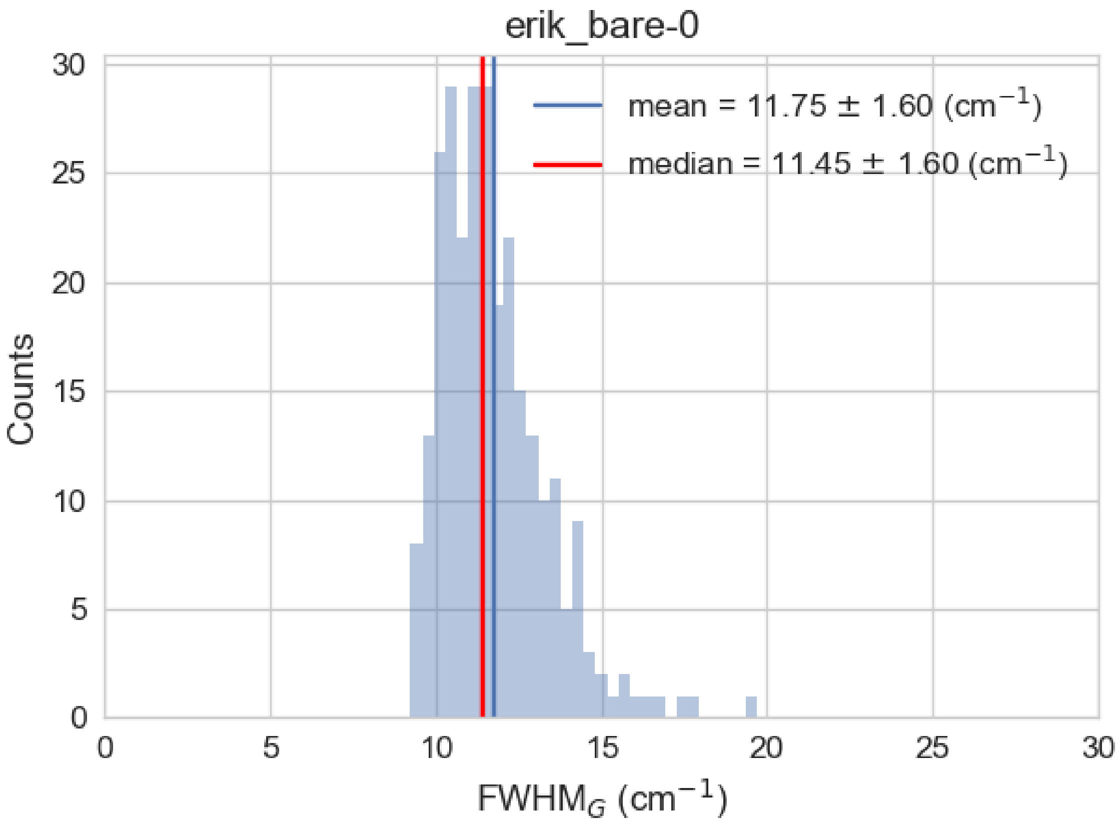

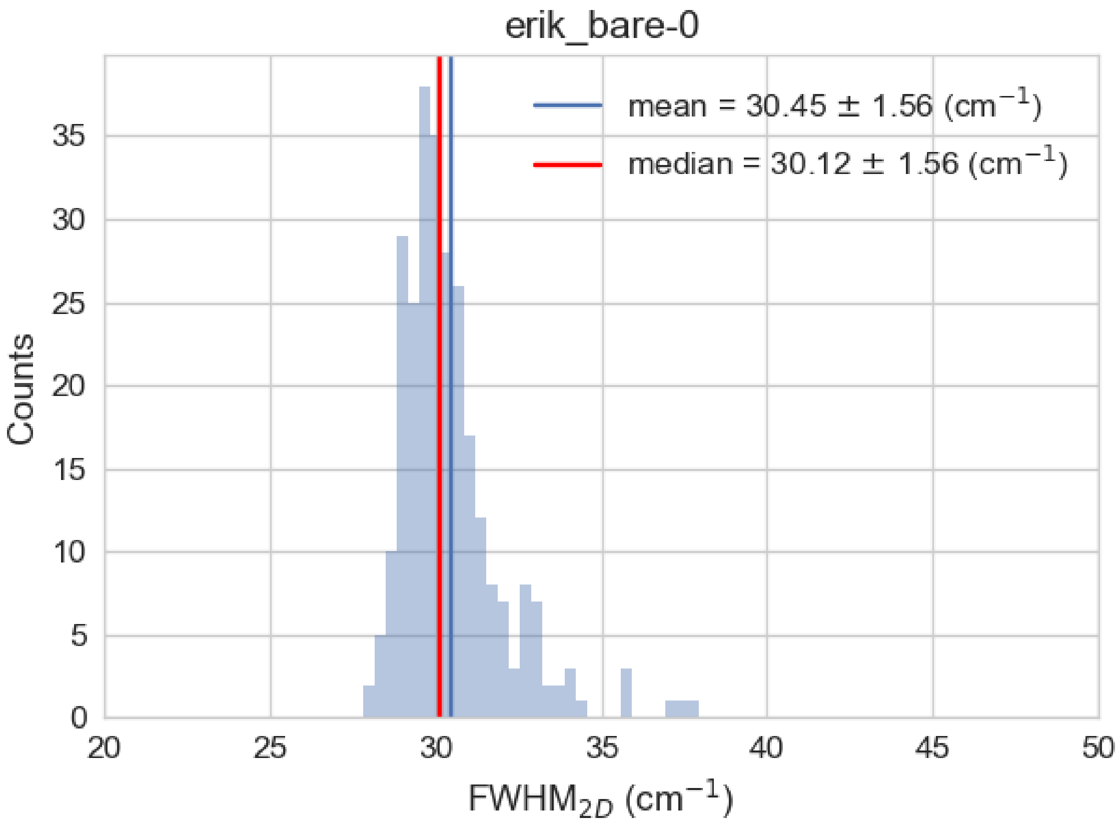

erik_bare-0: G & 2D Intensity Ratios

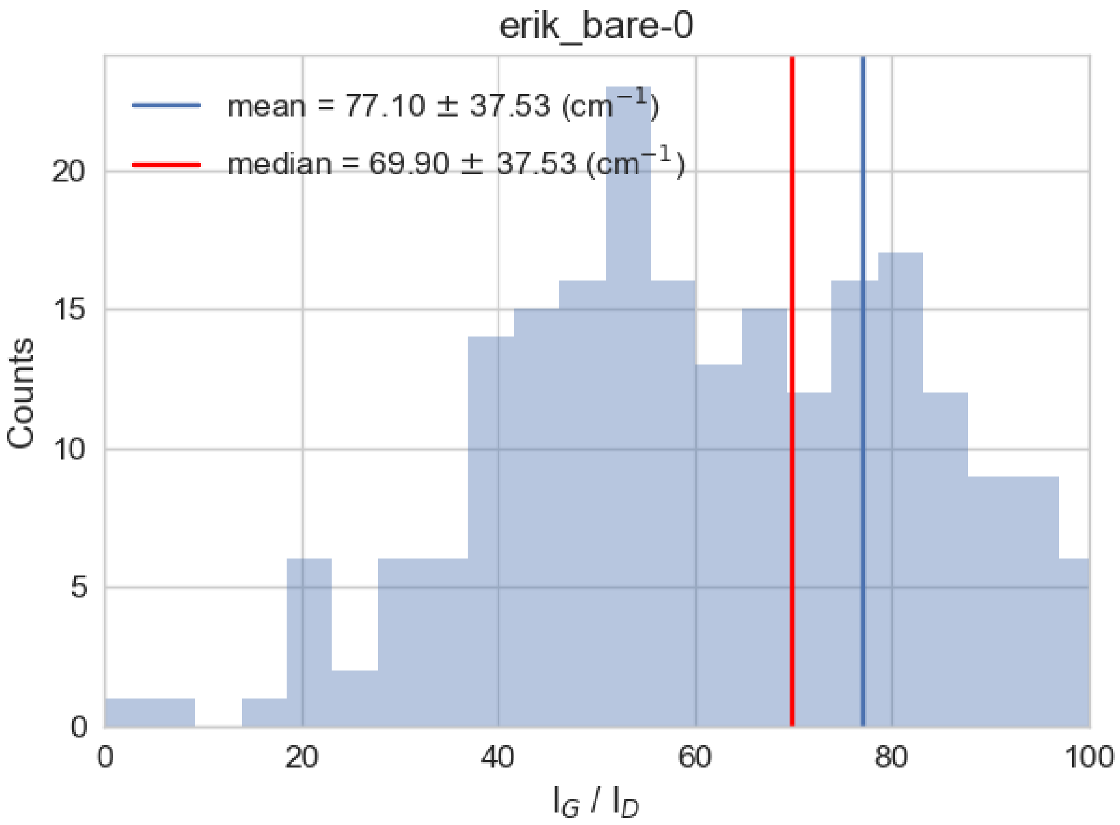

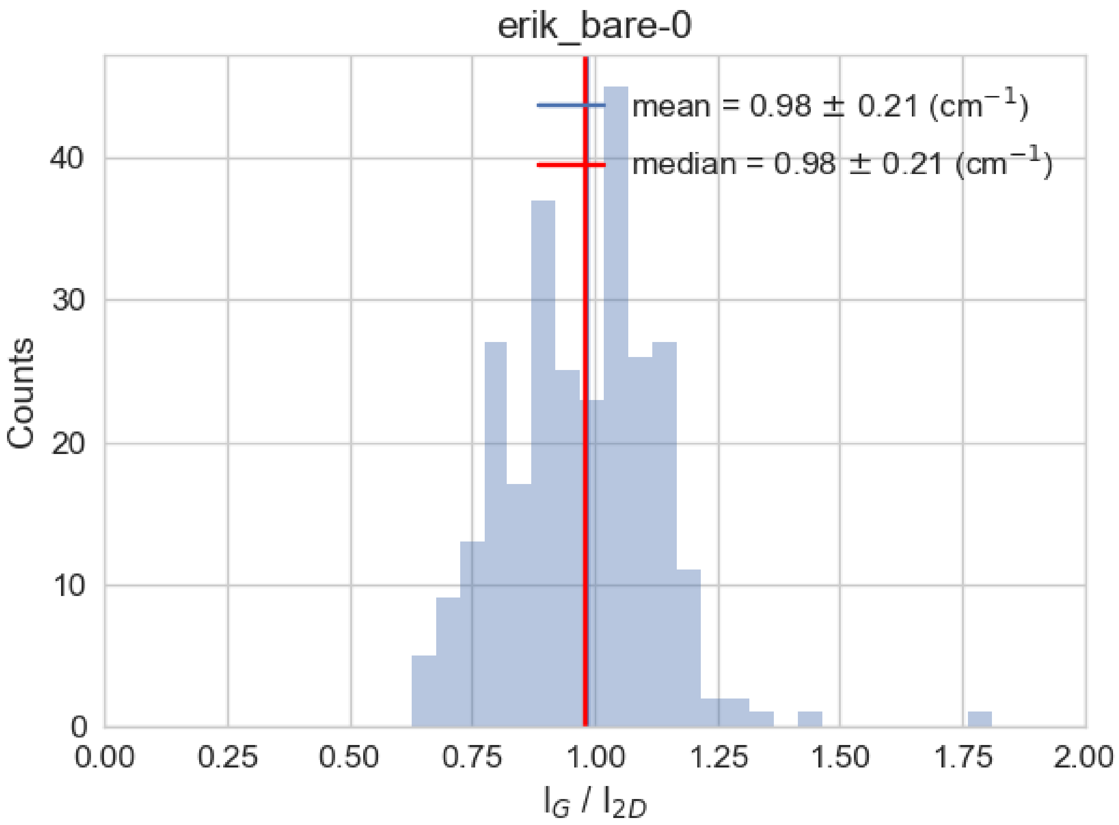

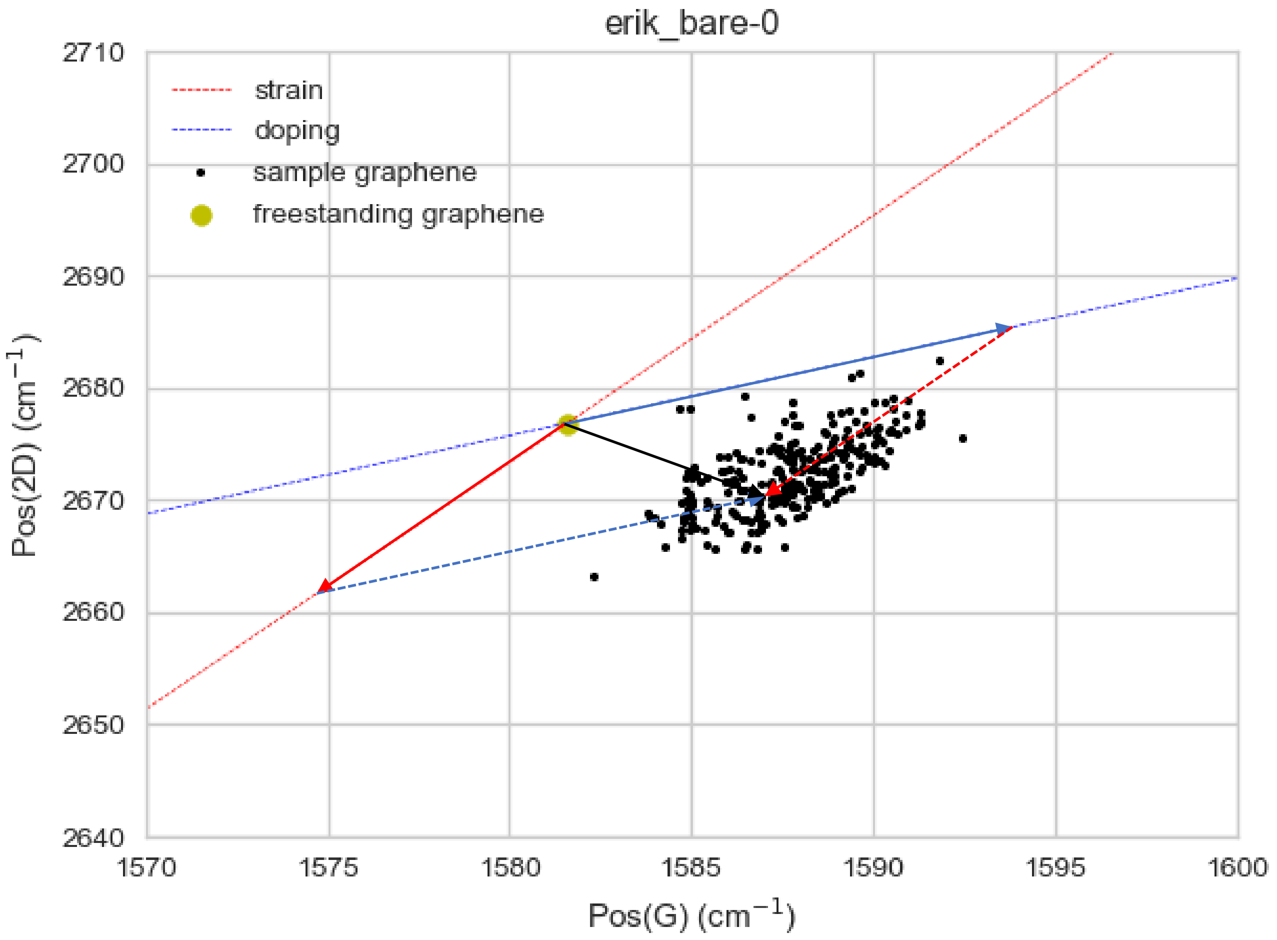
erik_bare-0
strain
doping
sample graphene
freestanding graphene
Pos(2D) (cm^{-1})
Pos(G) (cm^{-1})
2710
2700
2690
2680
2670
2660
2650
2640
1570
1575
1580
1585
1590
1595
1600

erik_bare-0: G & 2D Positions

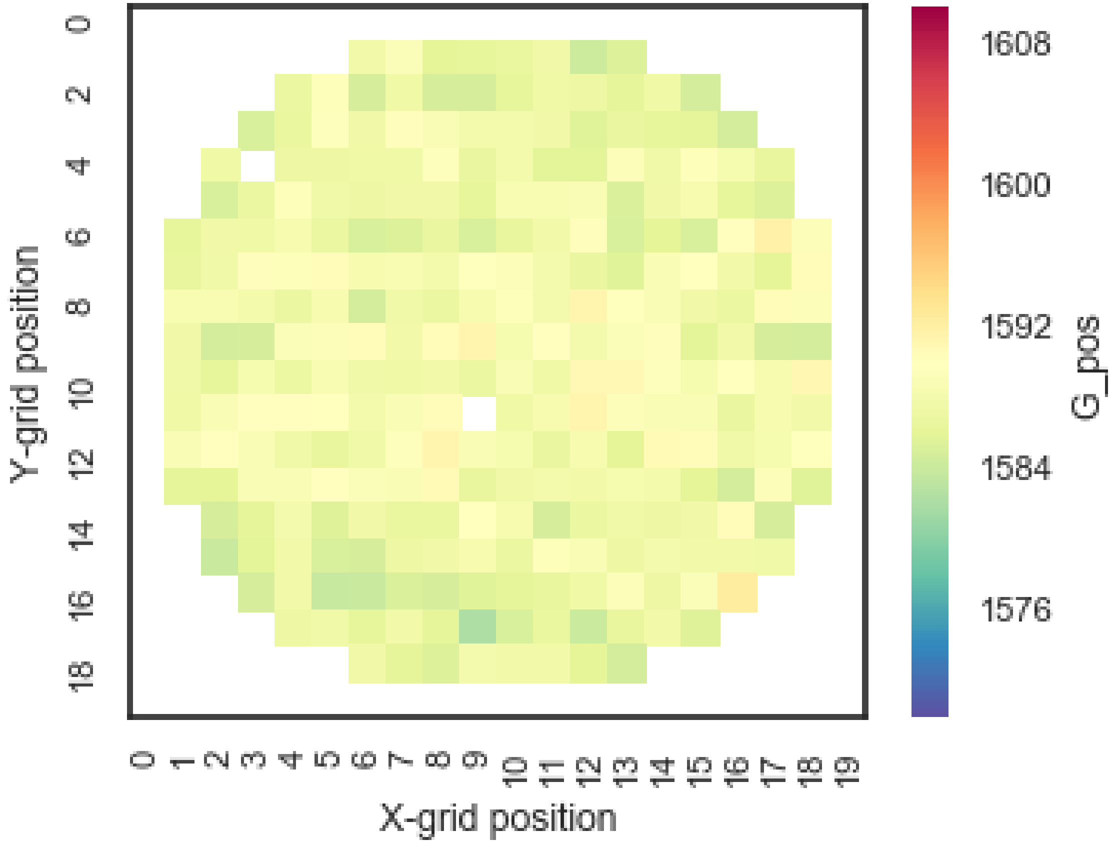

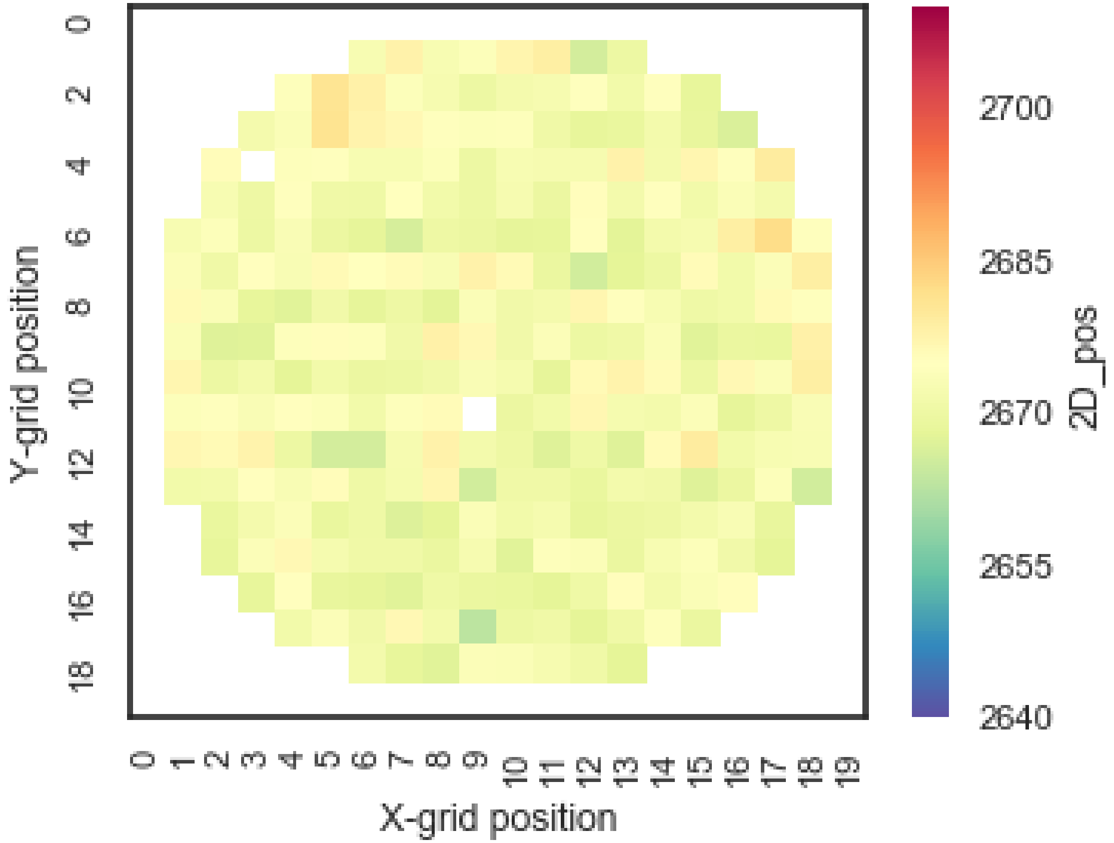

erik_bare-0: G & 2D FWHM

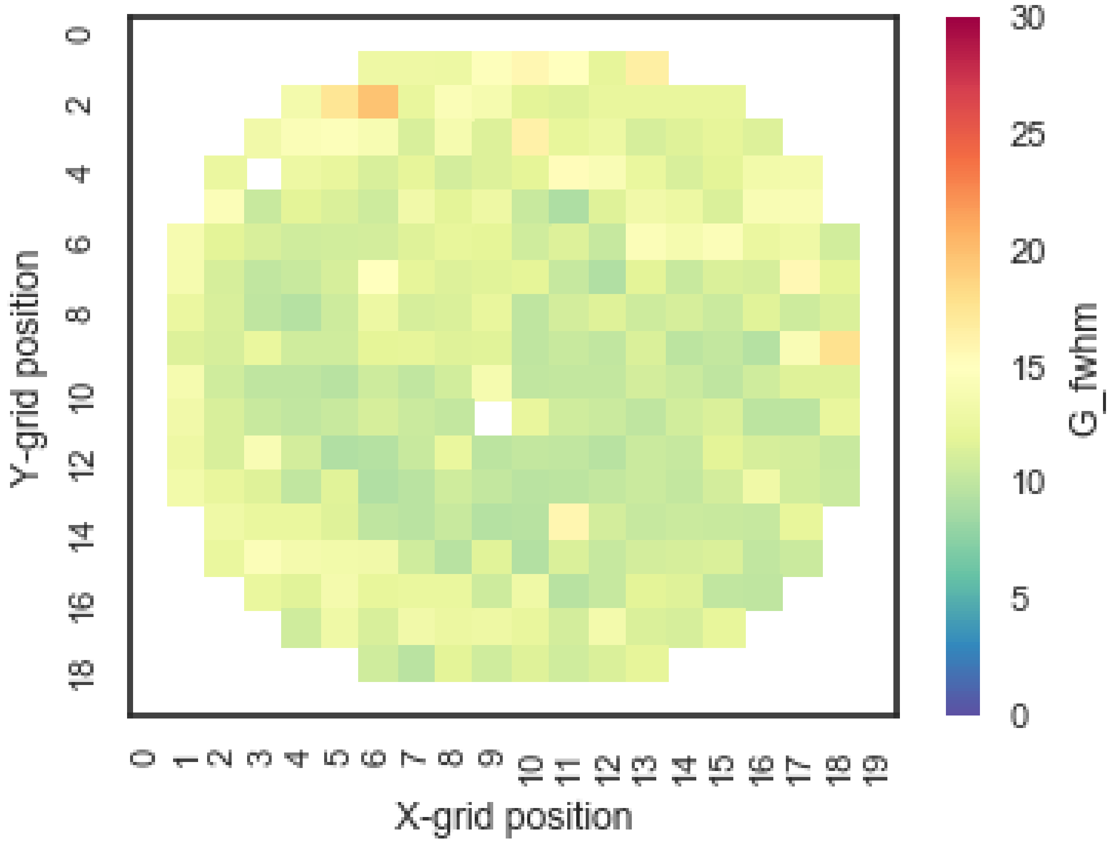

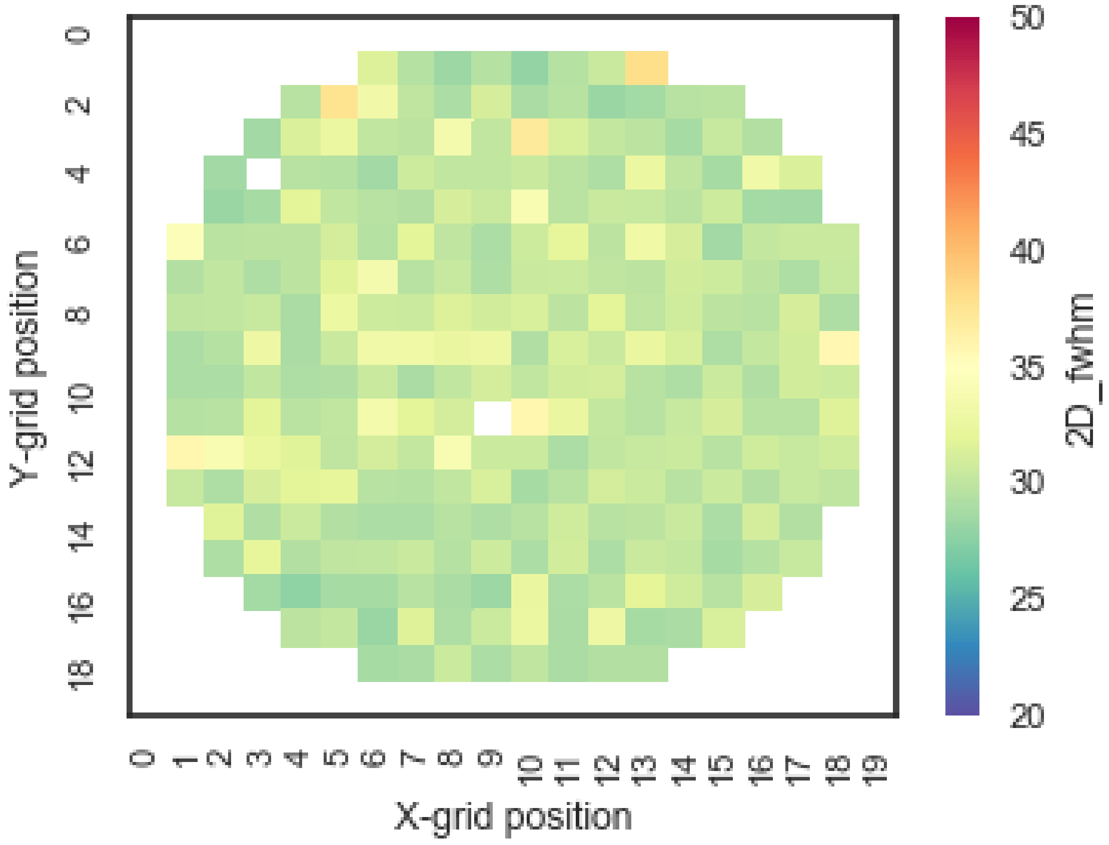

erik_bare-0: G & 2D Intensity Ratios

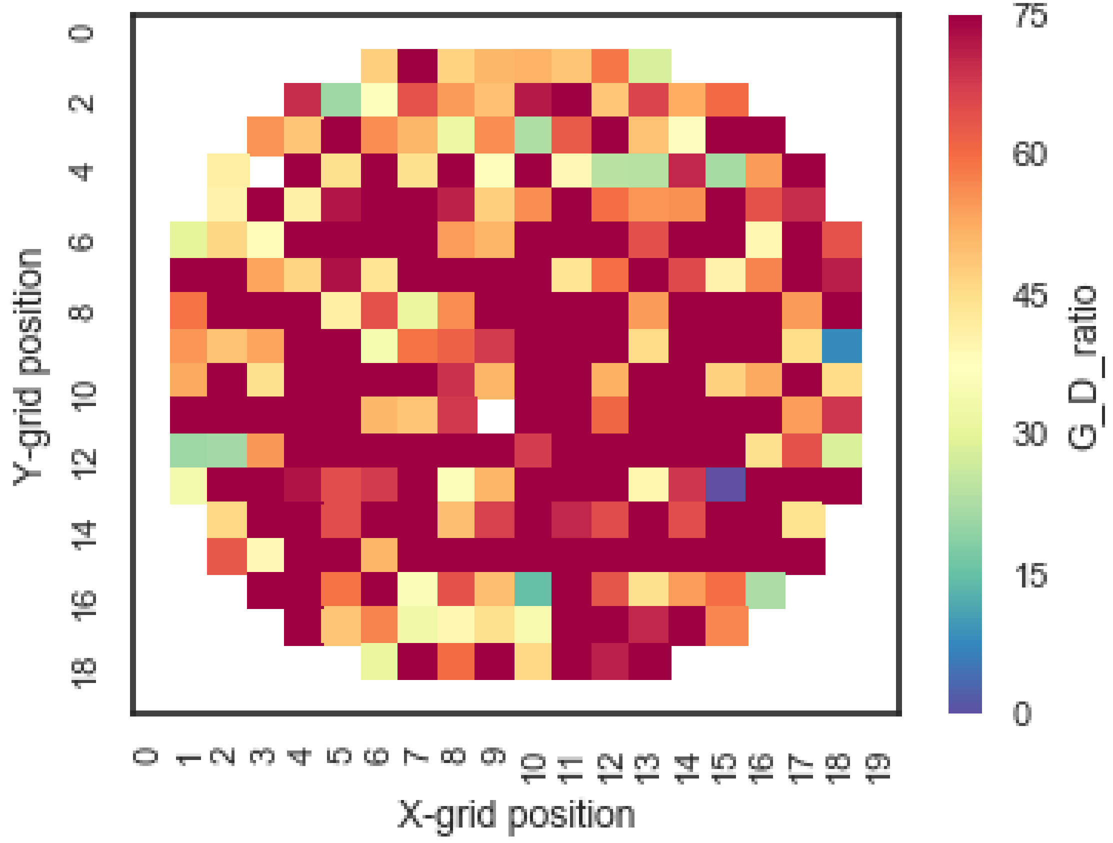

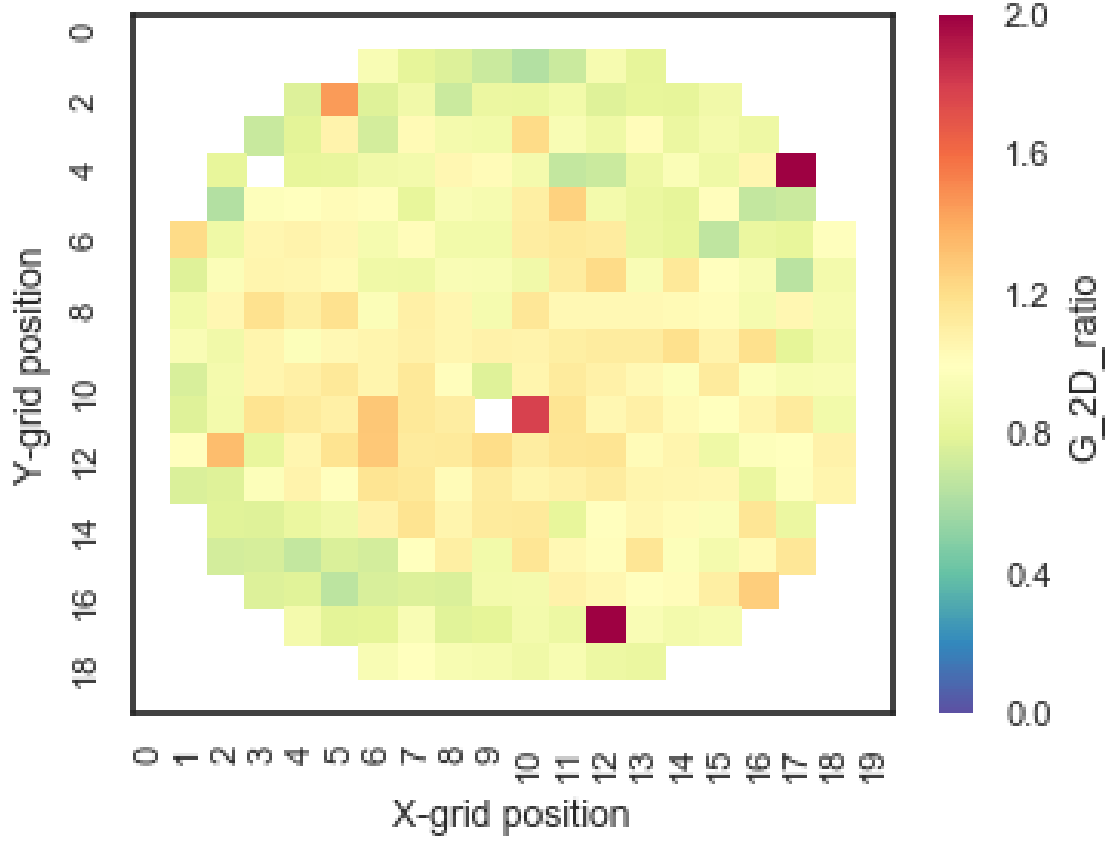

Raman data, graphene on ots wafer

3 random spectra

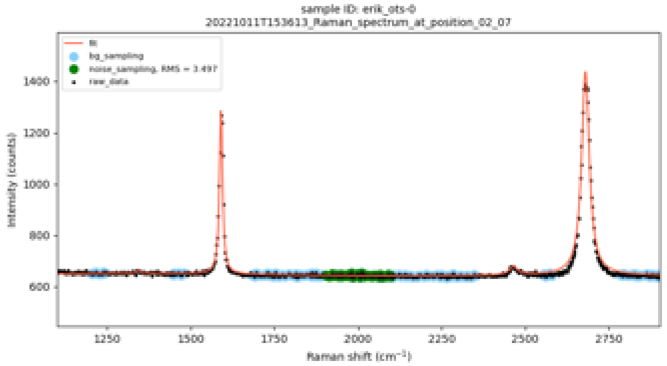

	FWHM (cm⁻¹)	Pos (cm⁻¹)	Int (counts)	R^2
Si	5.082	520.003	15052.689	0.90348
D	14.462	1343.733	9.801	0.99995
G	10.982	1590.688	646.688	0.99911
D+D''	29.072	2464.851	33.065	0.99994
2D	27.598	2680.904	794.799	0.99732

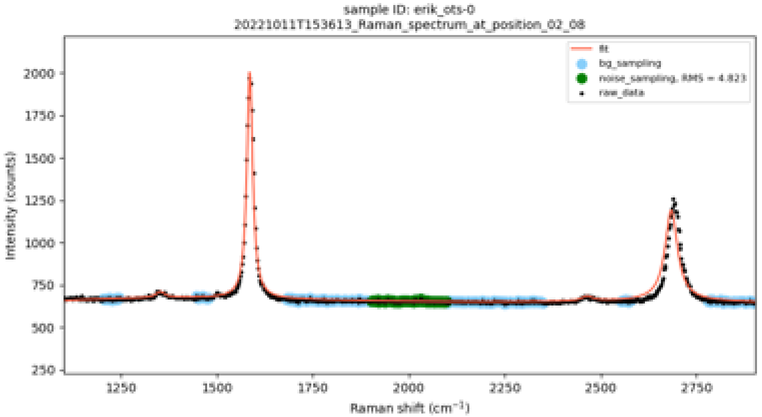

	FWHM (cm⁻¹)	Pos (cm⁻¹)	Int (counts)	R^2
Si	5.258	520.012	14592.599	0.92012
D	28.016	1351.563	35.281	0.99982
G	18.146	1585.847	1341.308	0.99686
D+D''	33.898	2464.404	27.163	0.99991
2D	39.04	2684.702	534.325	0.97313

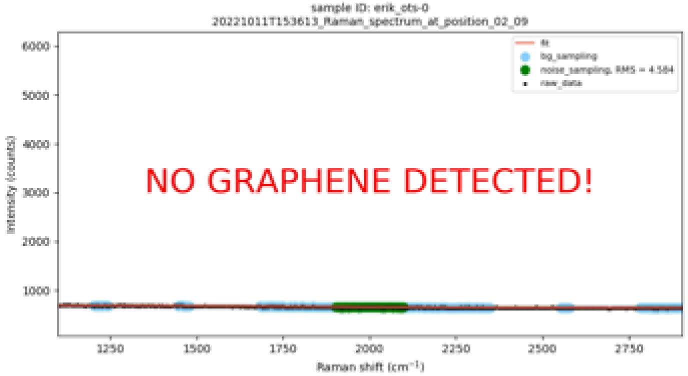

	FWHM (cm⁻¹)	Pos (cm⁻¹)	Int (counts)	R^2
Si	5.022	520.001	16263.58	0.88658
D	0.096	1368.724	5028.86	0.99996
G	4.0	1550.0	0.0	0.99996
D+D''	10.0	2479.92	7.512	0.99996
2D	10.0	2640.0	0.0	0.99997

Summary of the Raman fits

Parameter	Value (mean)	Standard Deviation
Total Raman scans in measurement	276	N/A
Number of scans showing graphene	261	N/A
Percentage of scans with graphene	94 %	N/A
D position	1347.67	4.8
D intensity	52.59	589.87
D FWHM	23.16	8.41
G position	1589.66	1.86
G intensity	675.97	523.44
G FWHM	12.4	2.56
2D position	2683.15	3.82
2D intensity	757.78	310.33
2D FWHM	28.26	3.34
G/D Ratio	60.45	38.64

erik_ots-0: G & 2D Positions

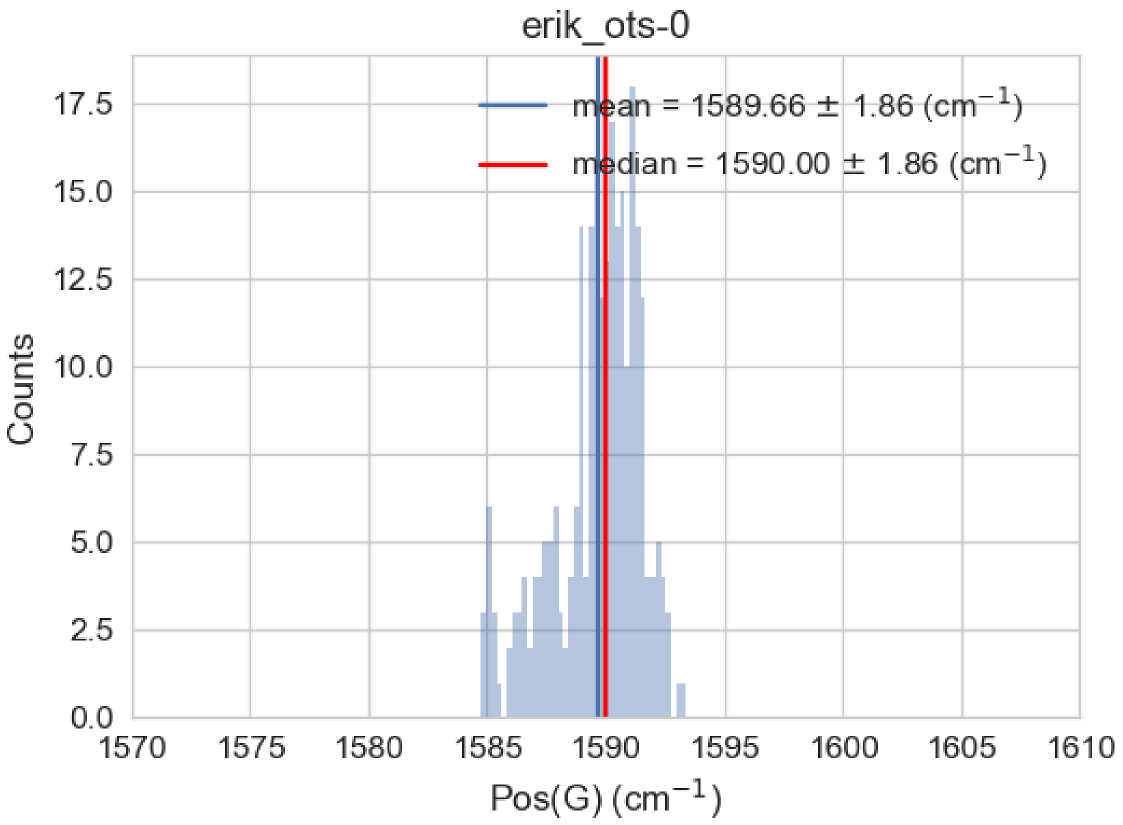

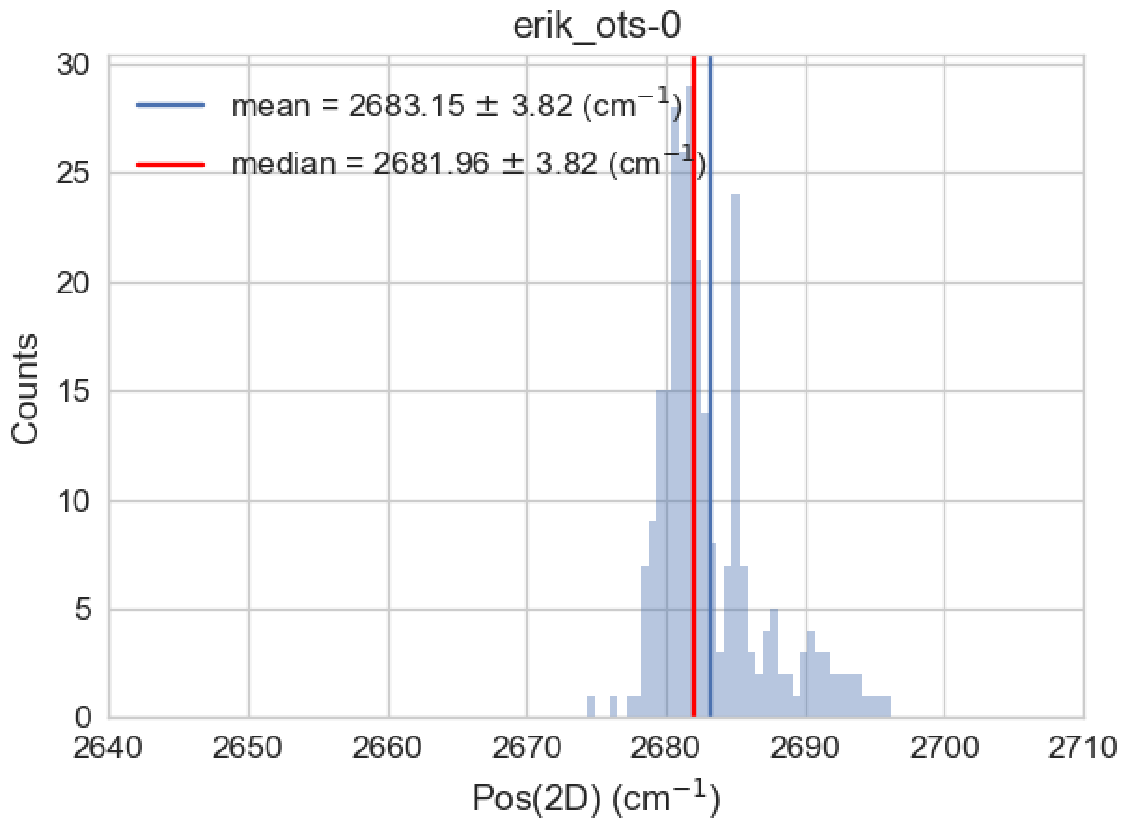

erik_ots-0: G & 2D FWHM

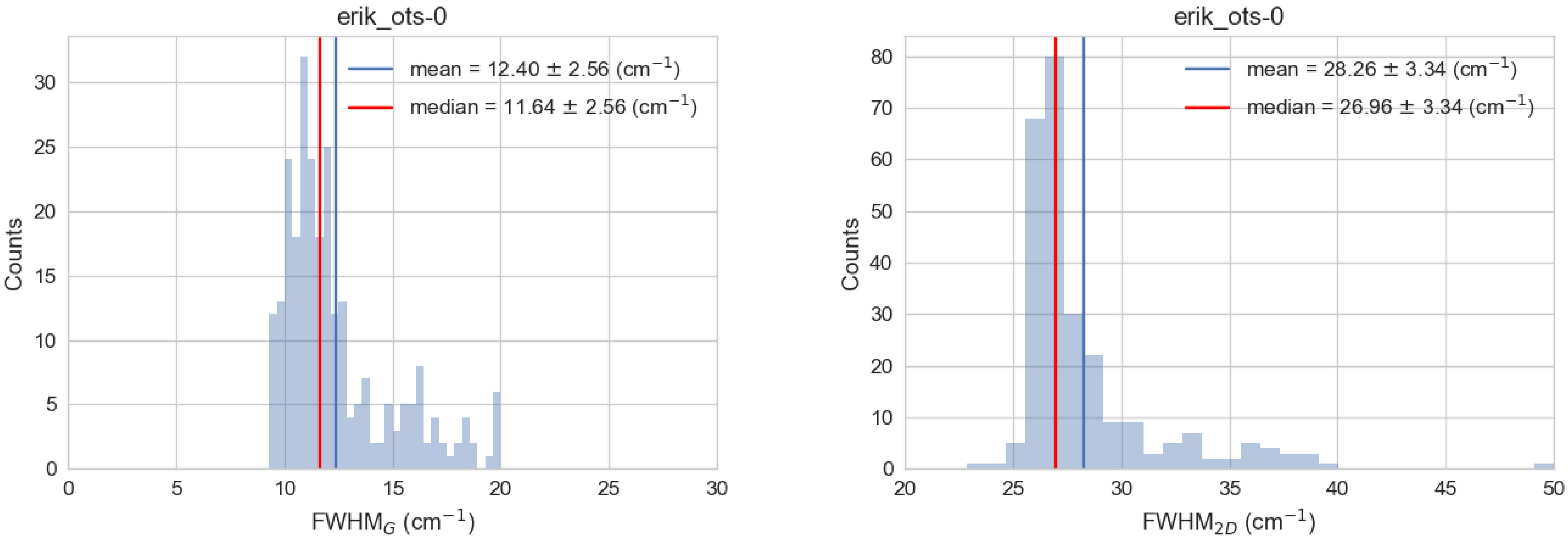

erik_ots-0: G & 2D Intensity Ratios

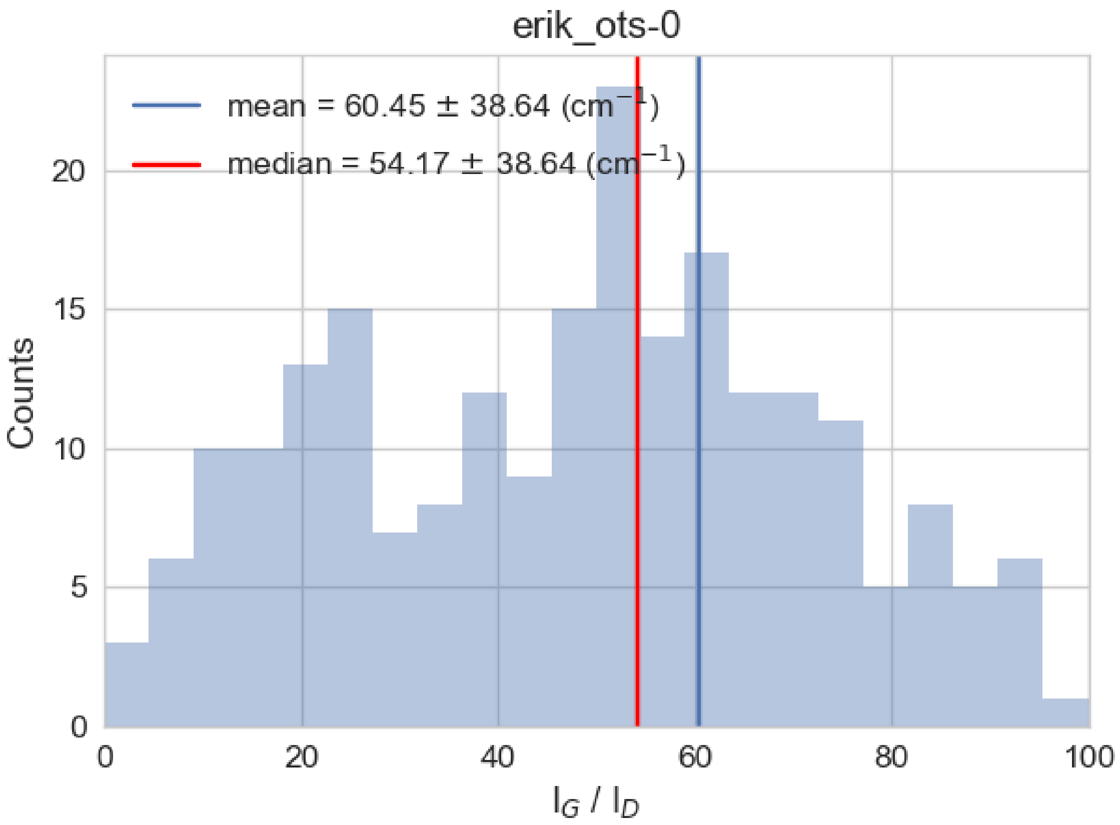

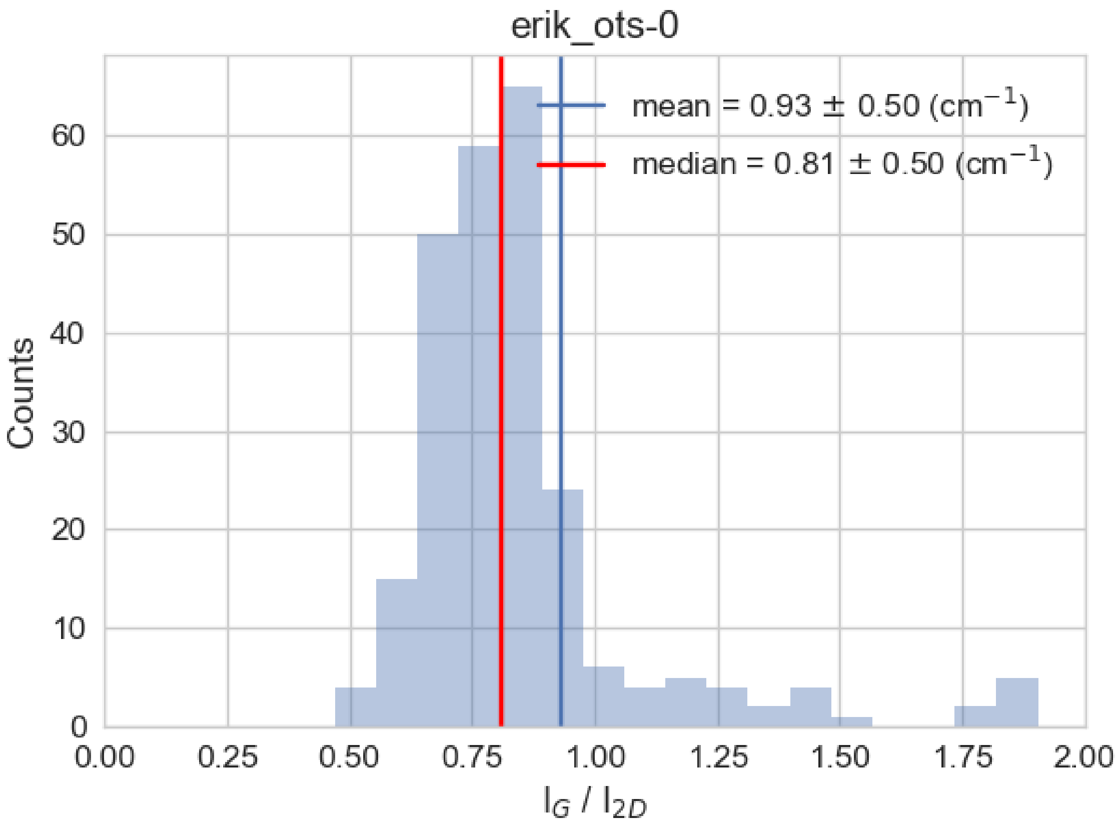

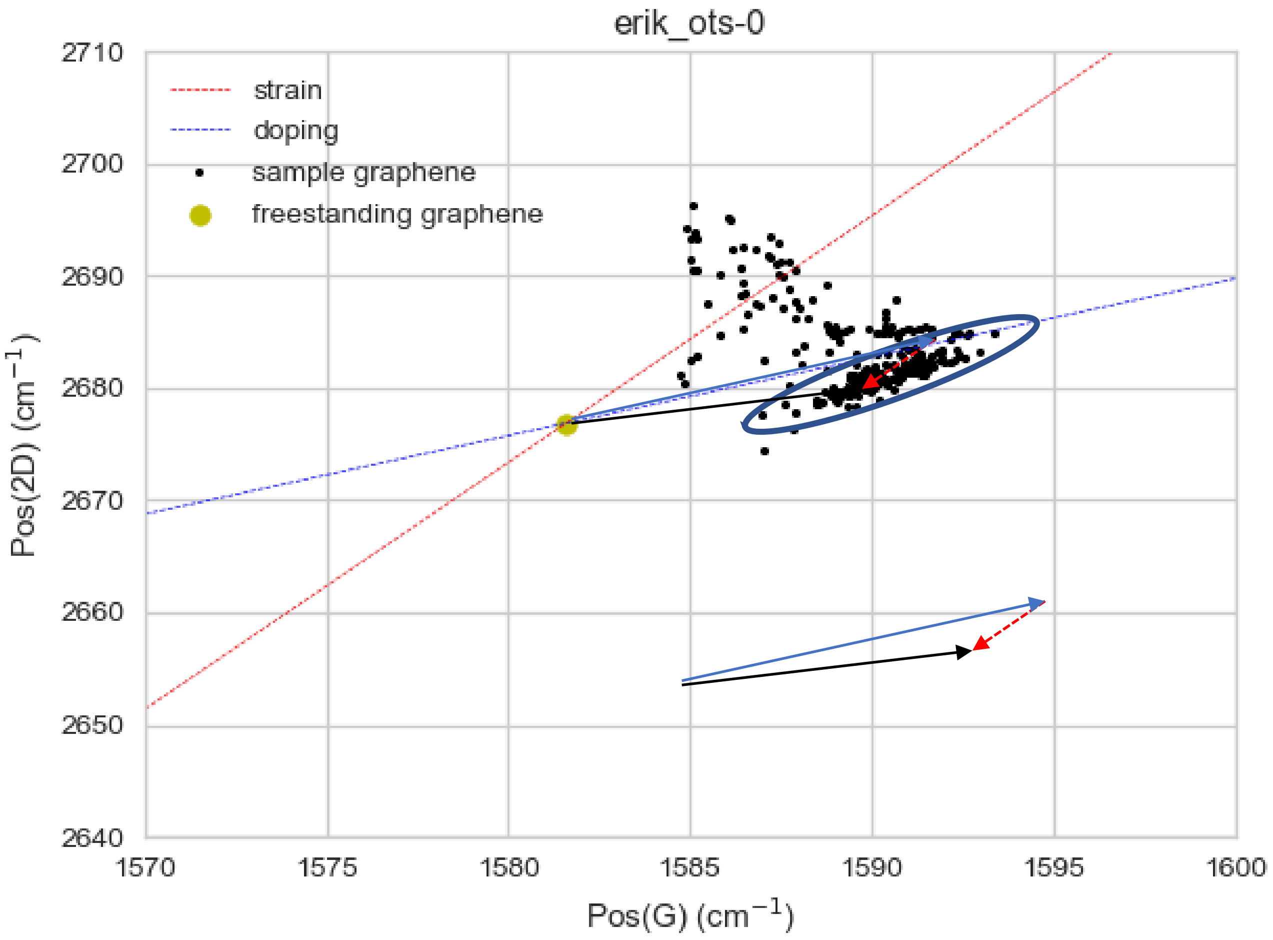
erik_ots-0
strain
doping
sample graphene
freestanding graphene
Pos(2D) (cm^{-1})
Pos(G) (cm^{-1})
2710
2700
2690
2680
2670
2660
2650
2640
1570
1575
1580
1585
1590
1595
1600

erik_ots-0: G & 2D Positions

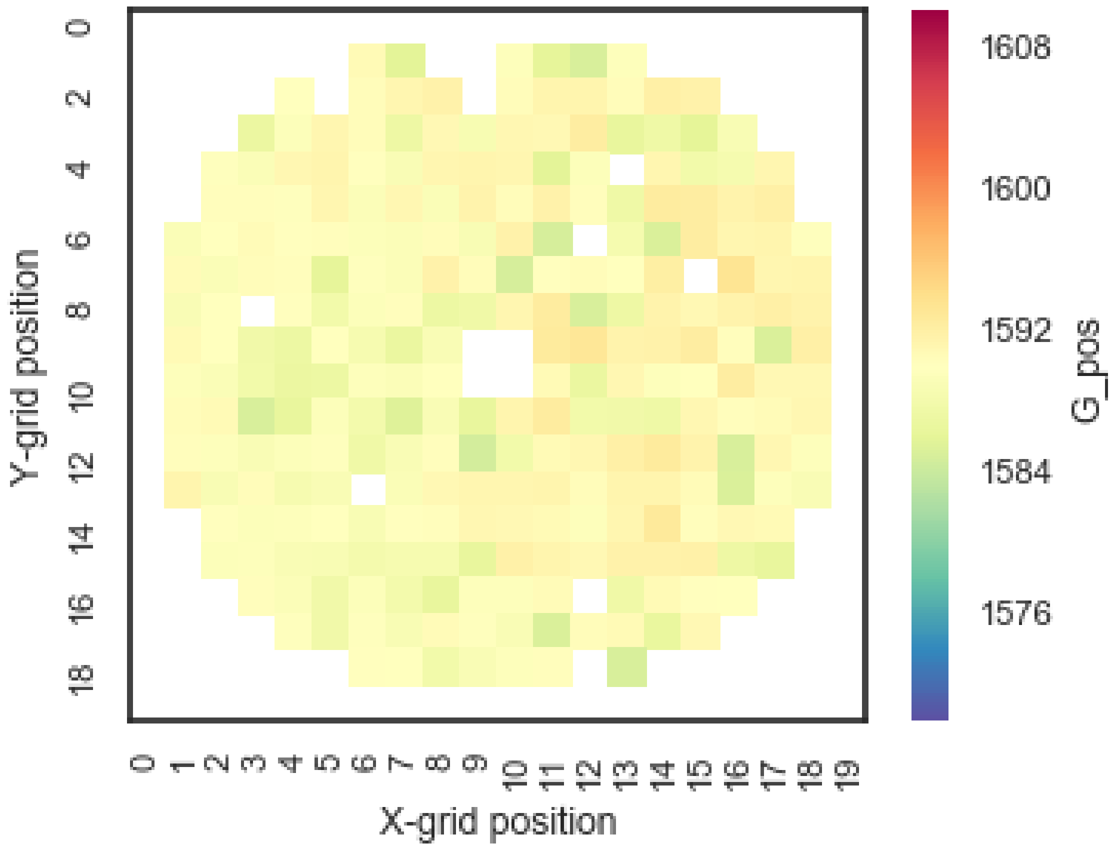

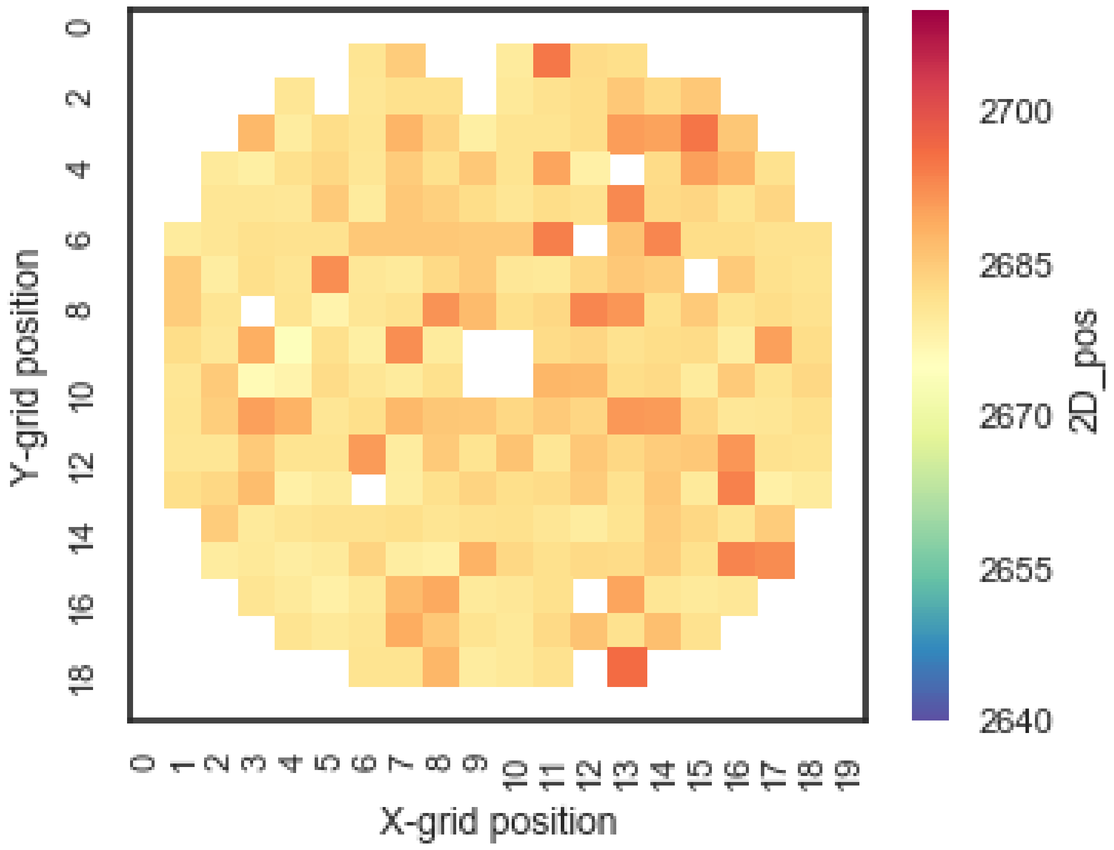

erik_ots-0: G & 2D FWHM

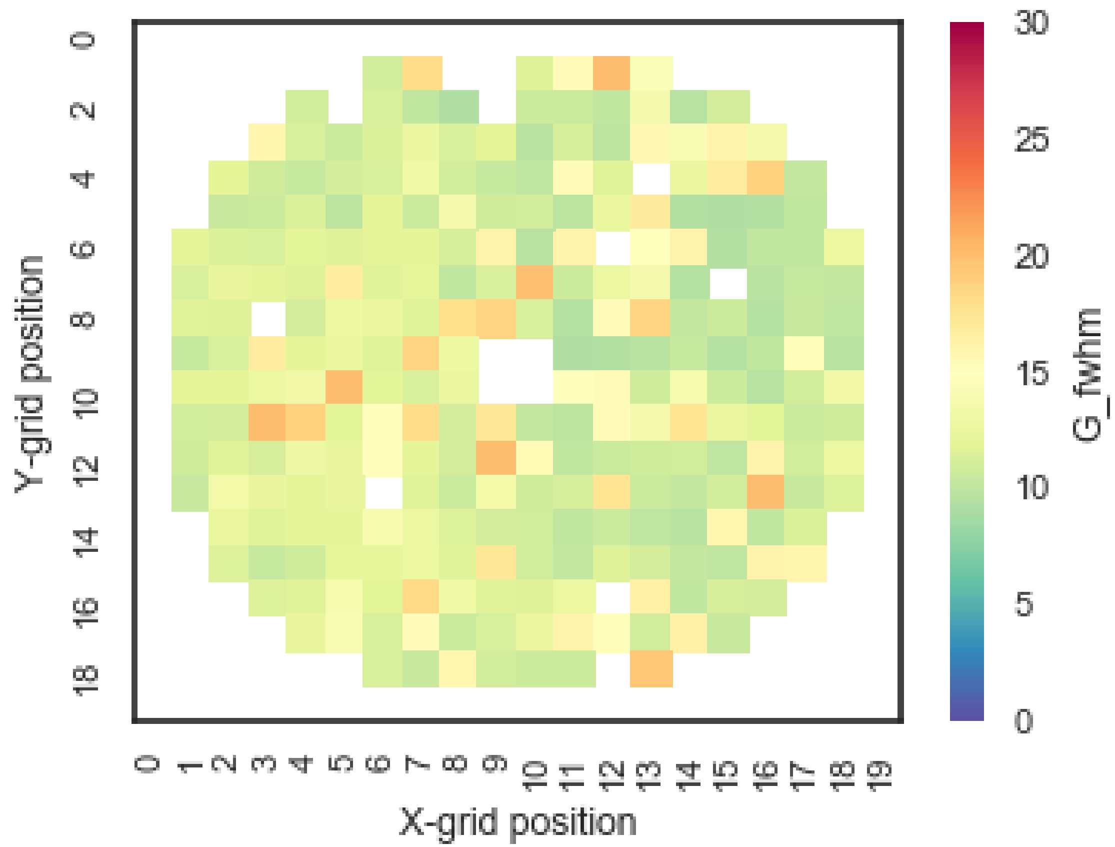

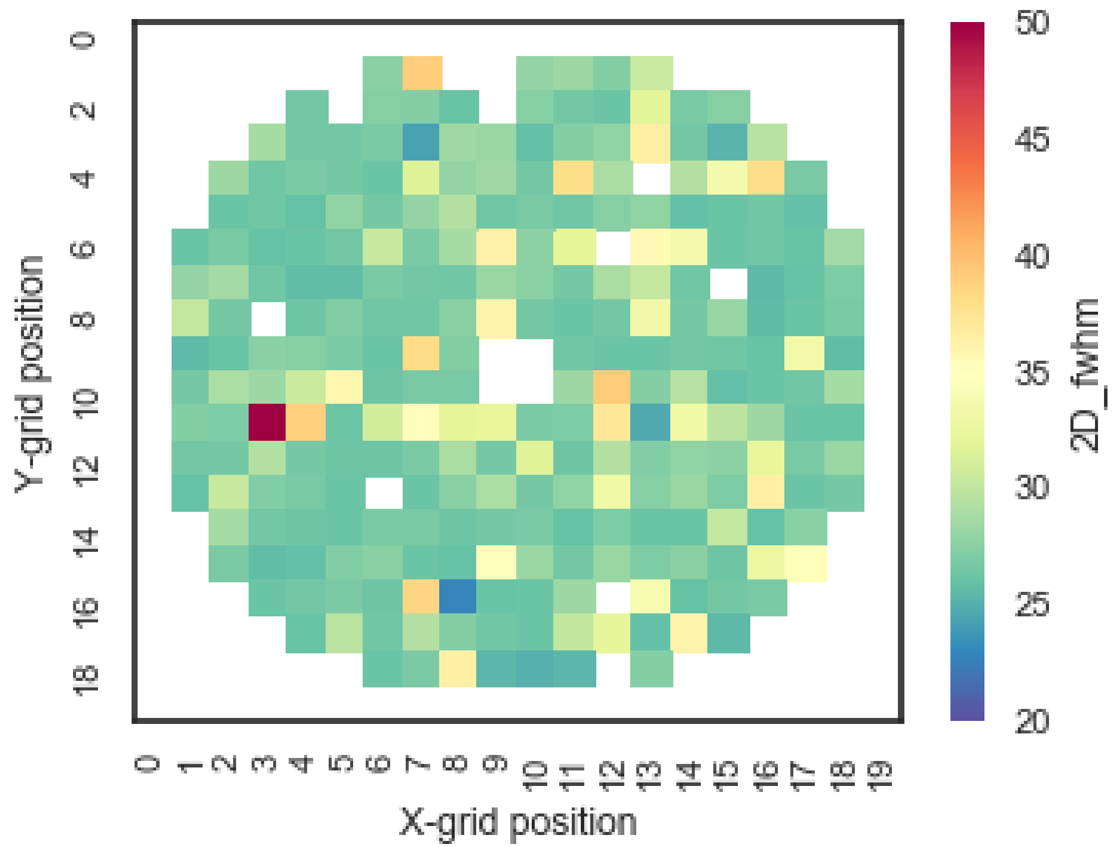

erik_ots-0: G & 2D Intensity Ratios

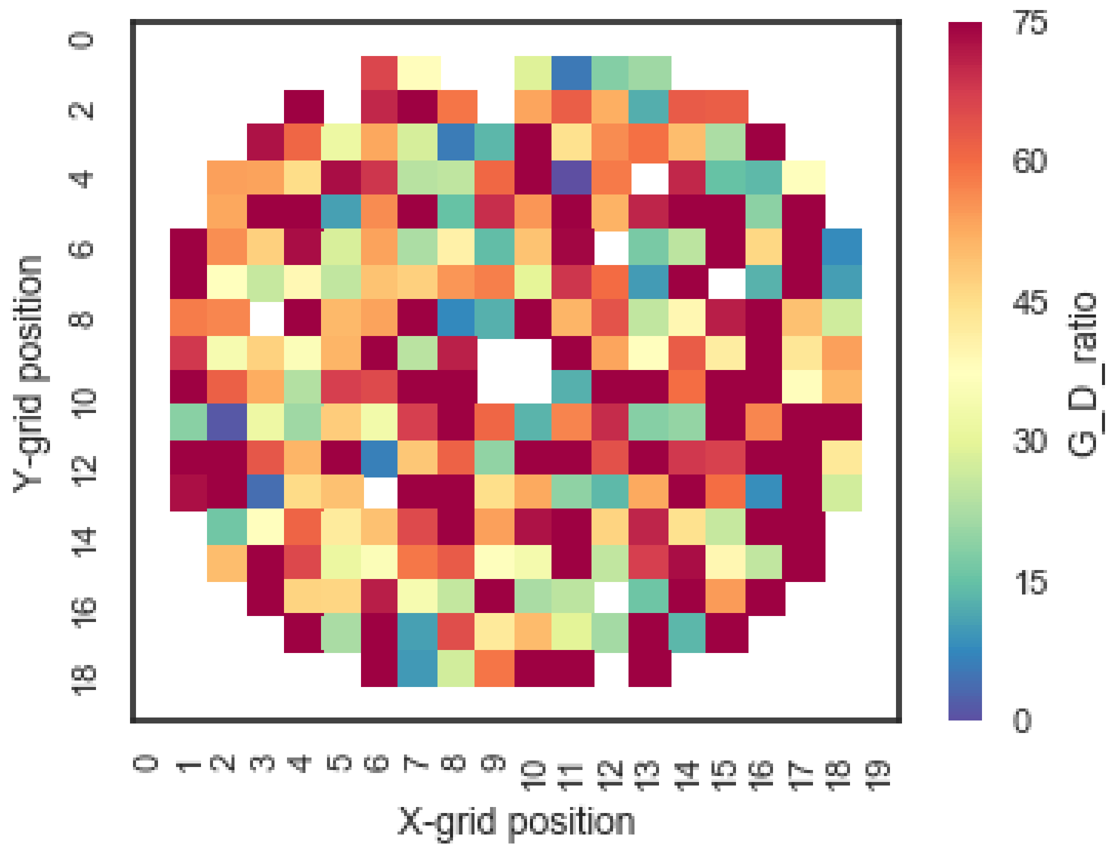

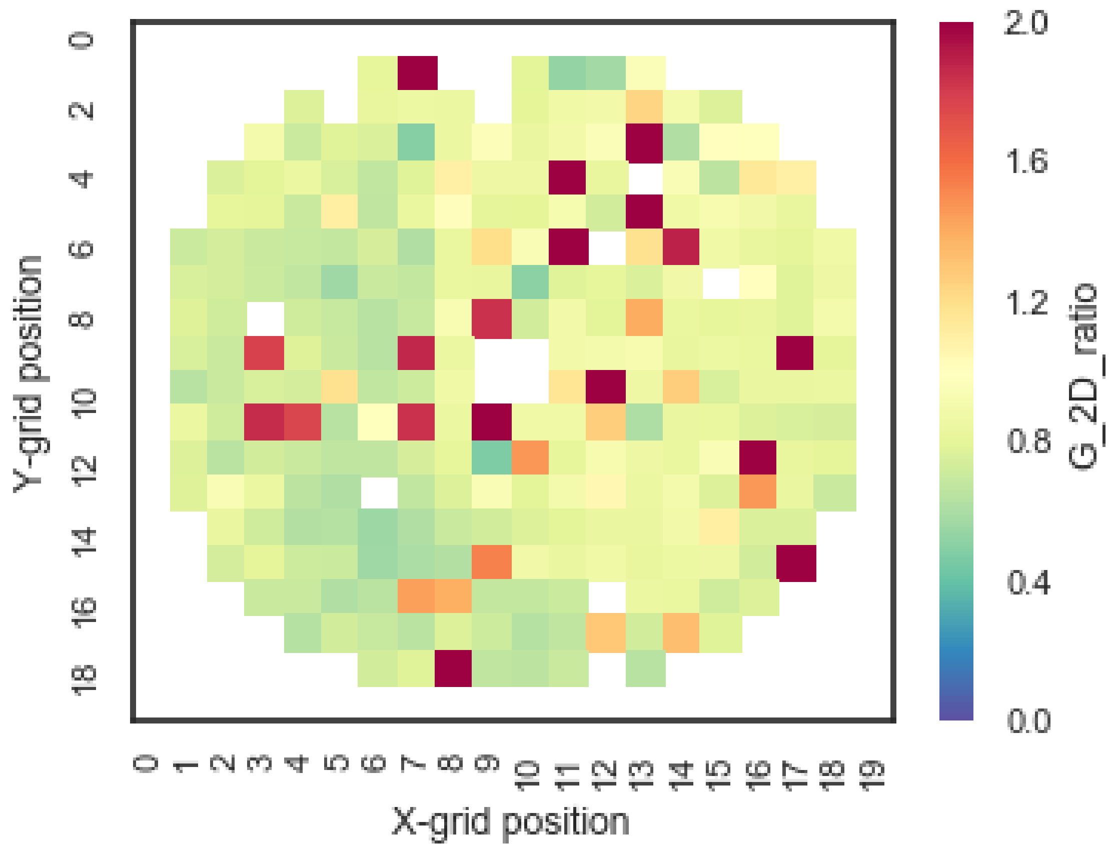

Raman data, graphene on pyrene wafer

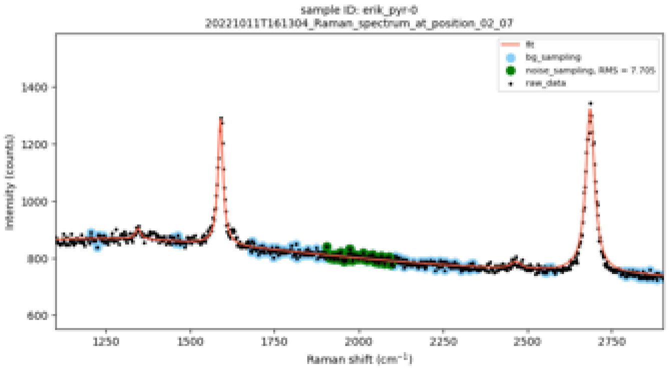

	FWHM (cm⁻¹)	Pos (cm⁻¹)	Int (counts)	R^2
Si	5.022	520.003	15356.09	0.9201
D	18.454	1345.305	38.244	0.99972
G	18.146	1590.009	438.136	0.99916
D+D''	34.374	2466.124	24.58	0.99974
2D	30.94	2686.469	572.774	0.99848

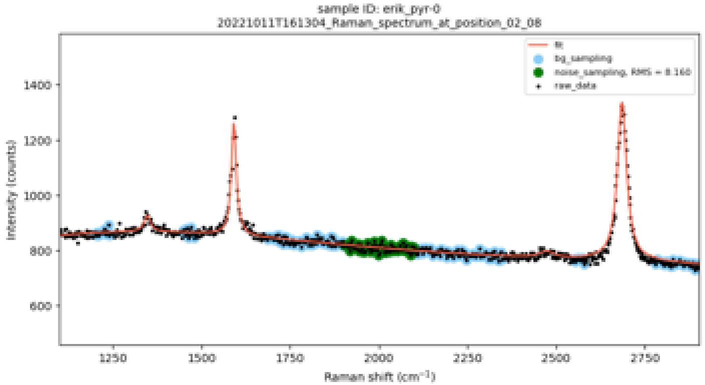

	FWHM (cm⁻¹)	Pos (cm⁻¹)	Int (counts)	R^2
Si	5.098	520.003	15126.19	0.9319
D	21.376	1345.844	58.824	0.99963
G	16.88	1590.903	408.678	0.99896
D+D''	48.032	2474.137	22.392	0.99976
2D	31.118	2687.111	574.212	0.99887

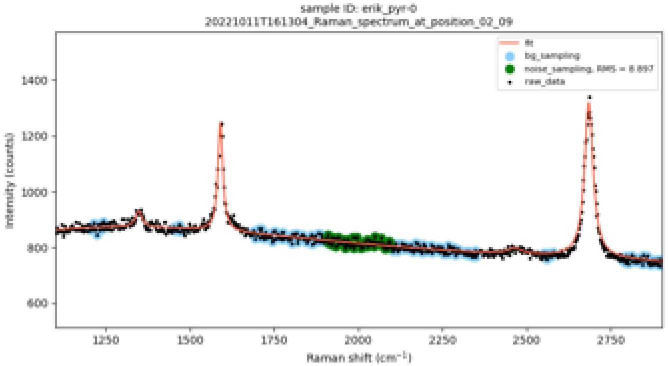

	FWHM (cm⁻¹)	Pos (cm⁻¹)	Int (counts)	R^2
Si	5.118	520.003	15282.975	0.92917
D	23.17	1347.352	57.624	0.99964
G	17.344	1590.255	388.129	0.99903
D+D''	50.0	2465.322	20.891	0.99978
2D	30.568	2685.261	555.742	0.99852

Parameter	Value (mean)	Standard Deviation
Total Raman scans in measurement	276	N/A
Number of scans showing graphene	274	N/A
Percentage of scans with graphene	99 %	N/A
D position	1347.44	5.14
D intensity	26.46	10.16
D FWHM	32.85	8.09
G position	1588.09	1.73
G intensity	490.3	43.76
G FWHM	16.54	1.17
2D position	2681.73	3.28
2D intensity	677.43	50.67
2D FWHM	29.87	1.09
G/D Ratio	20.33	5.89
G/2D Ratio	0.73	0.08

erik_pyr-0: G & 2D Positions

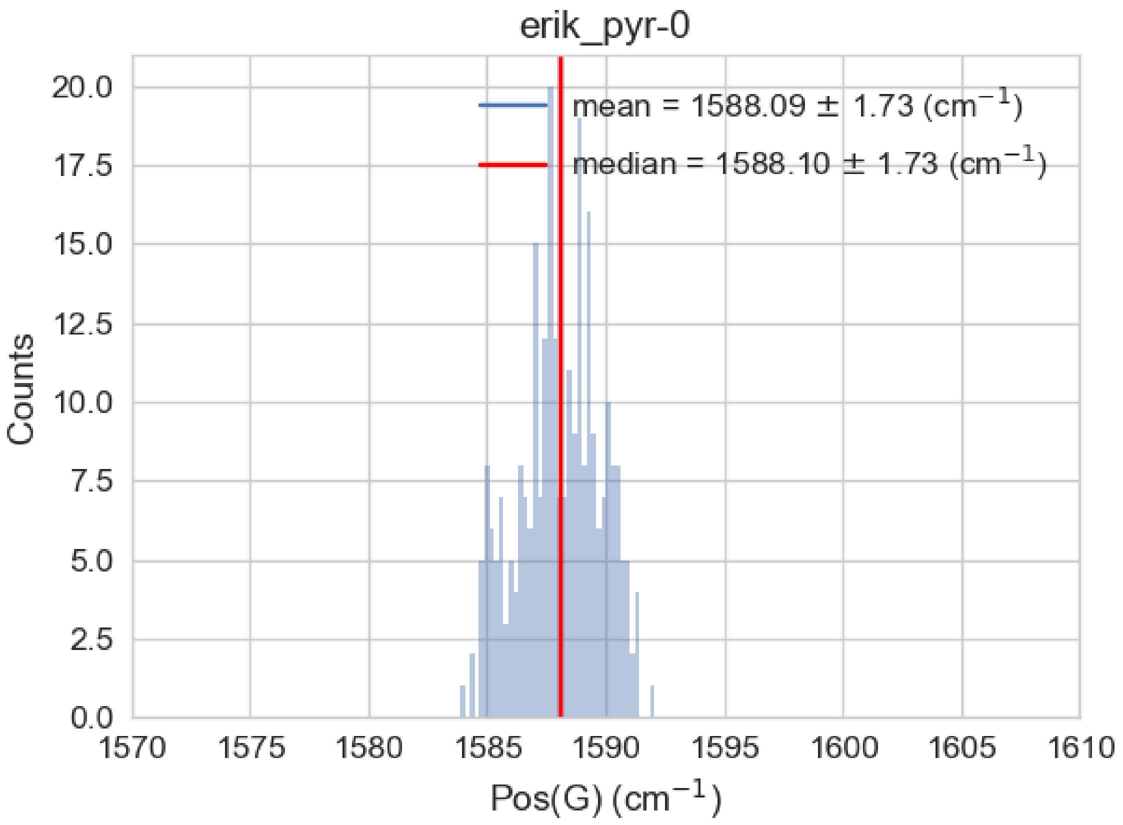

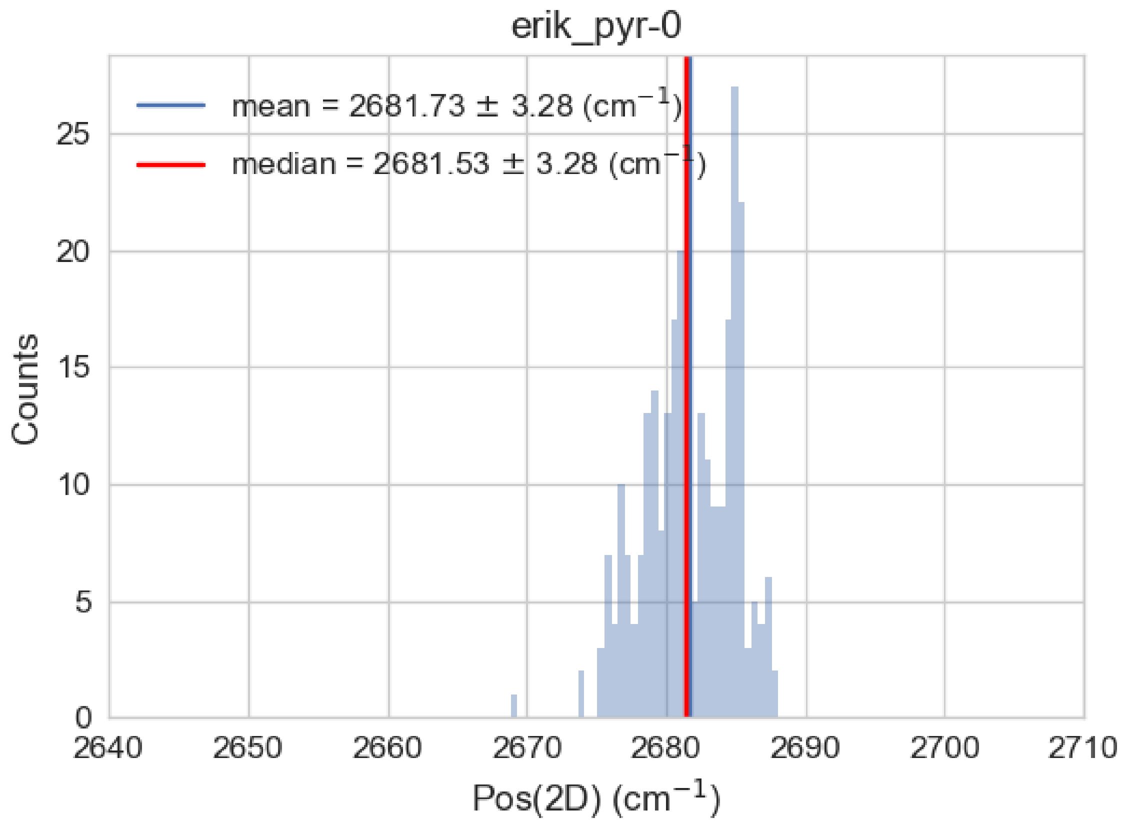

erik_pyr-0: G & 2D FWHM

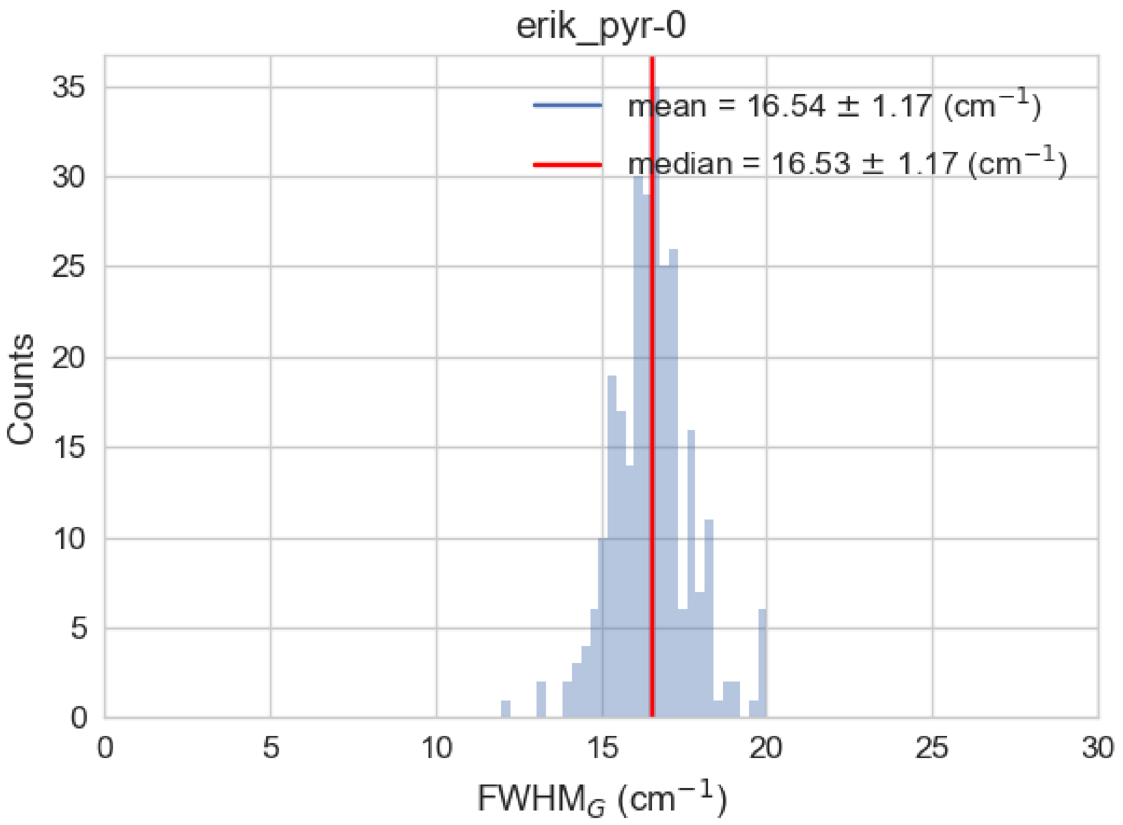

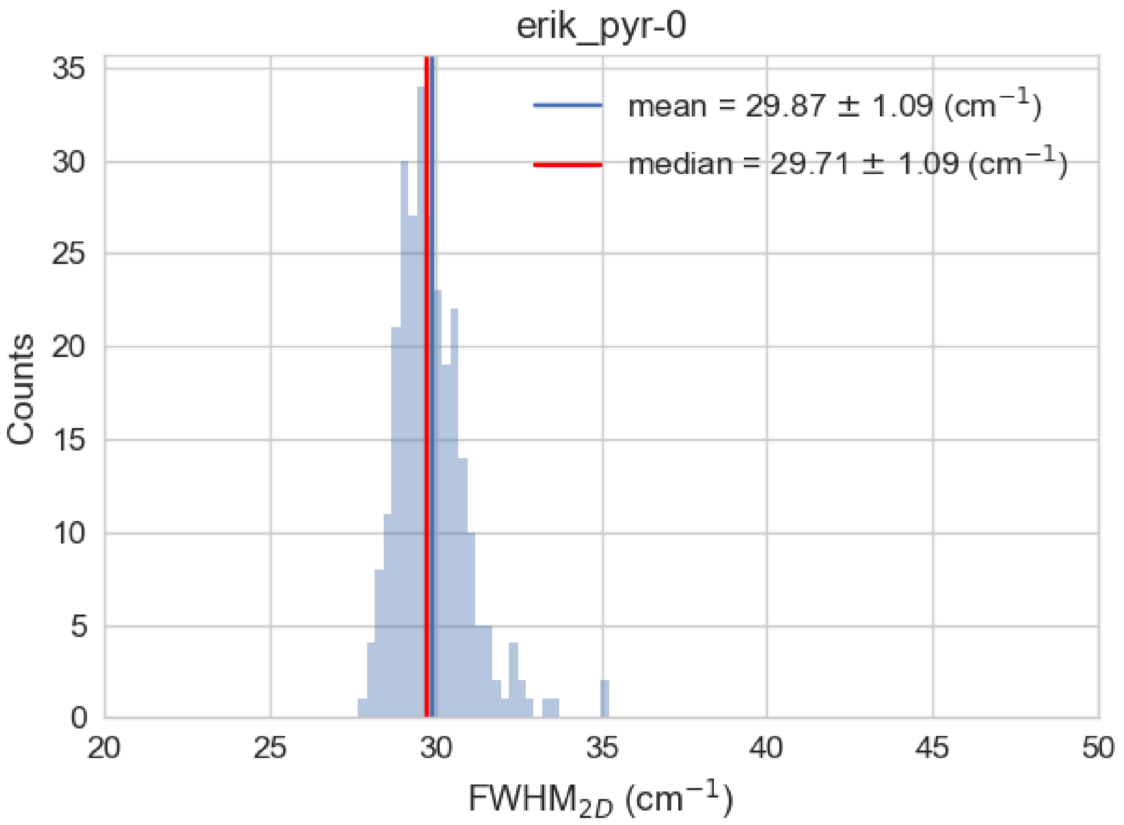

erik_pyr-0: G & 2D Intensity Ratios

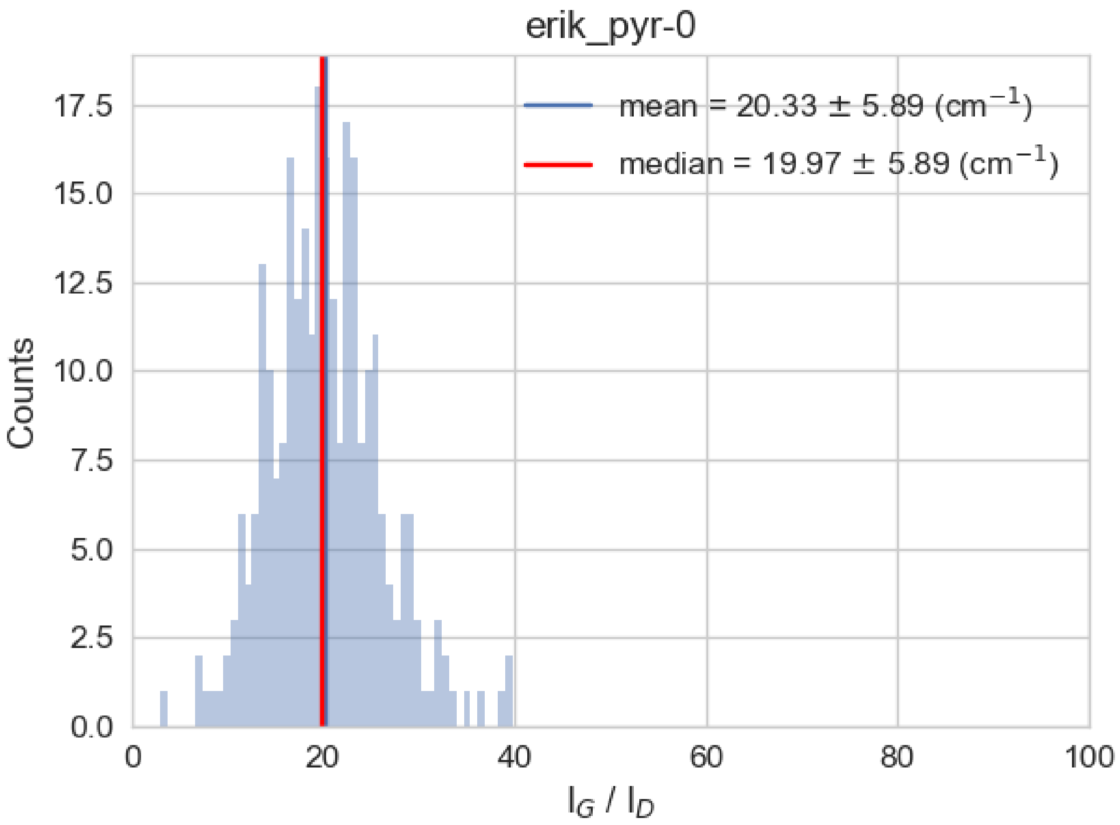

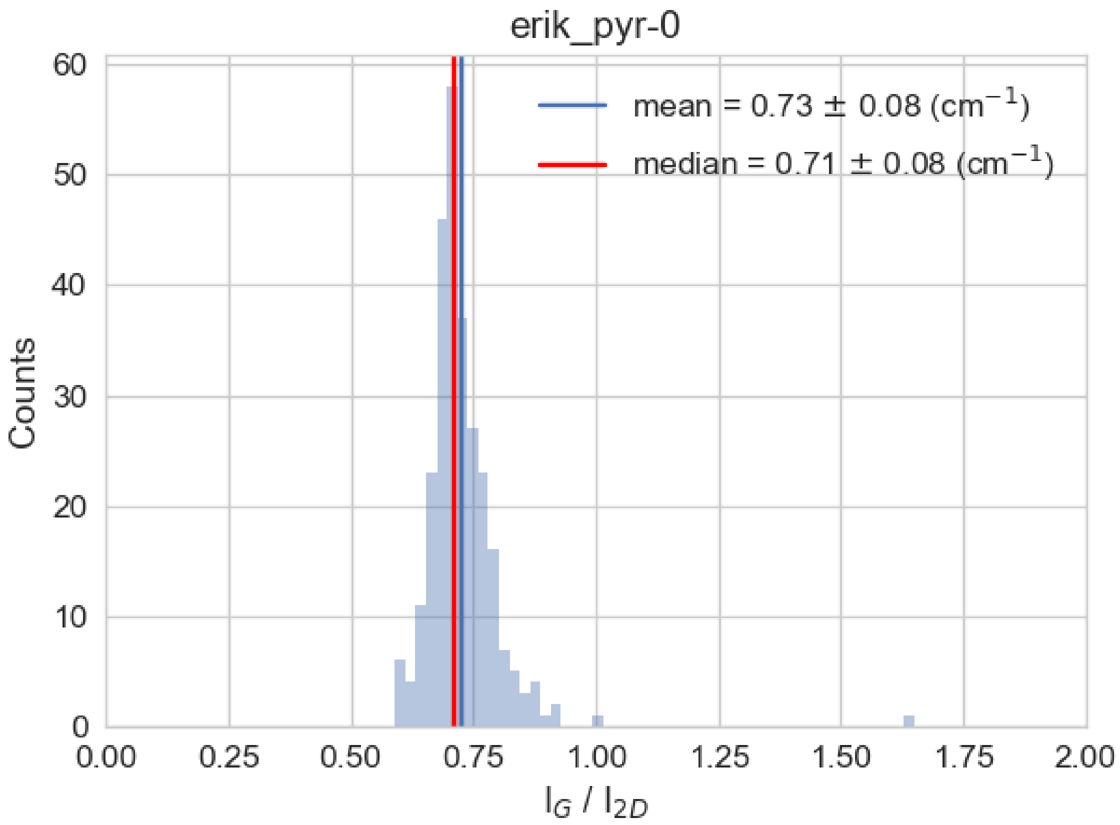

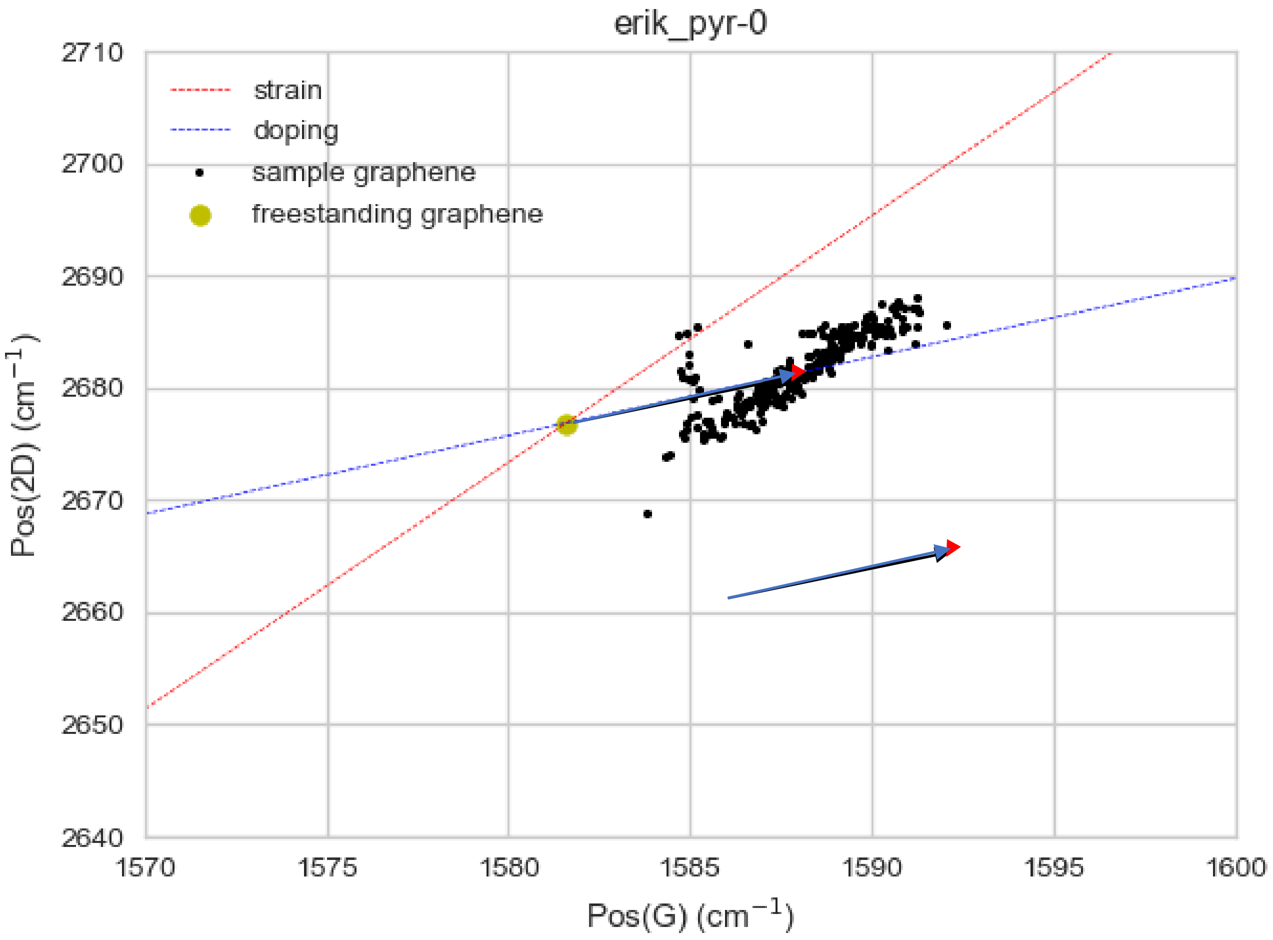
erik_pyr-0
strain
doping
sample graphene
freestanding graphene
2710
2700
2690
2680
2670
2660
2650
2640
1570
1575
1580
1585
1590
1595
1600
Pos(2D) (cm^{-1})
Pos(G) (cm^{-1})

erik_pyr-0: G & 2D Positions

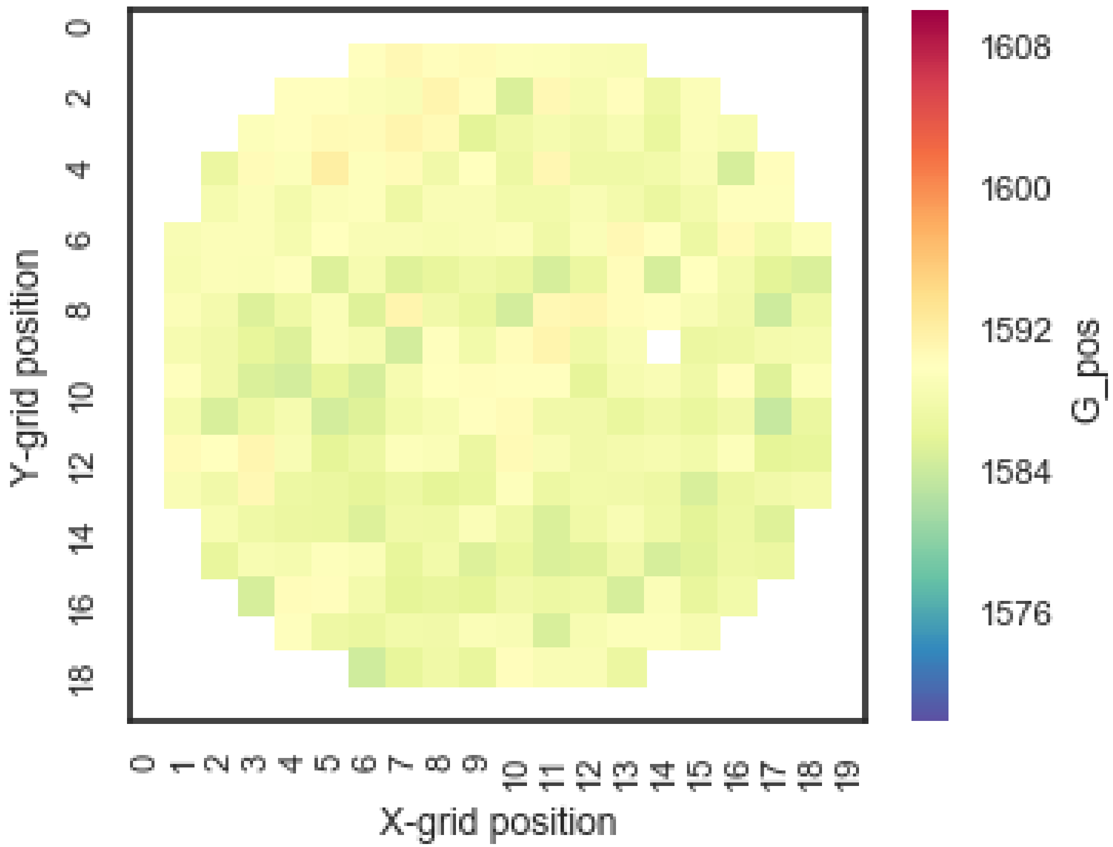

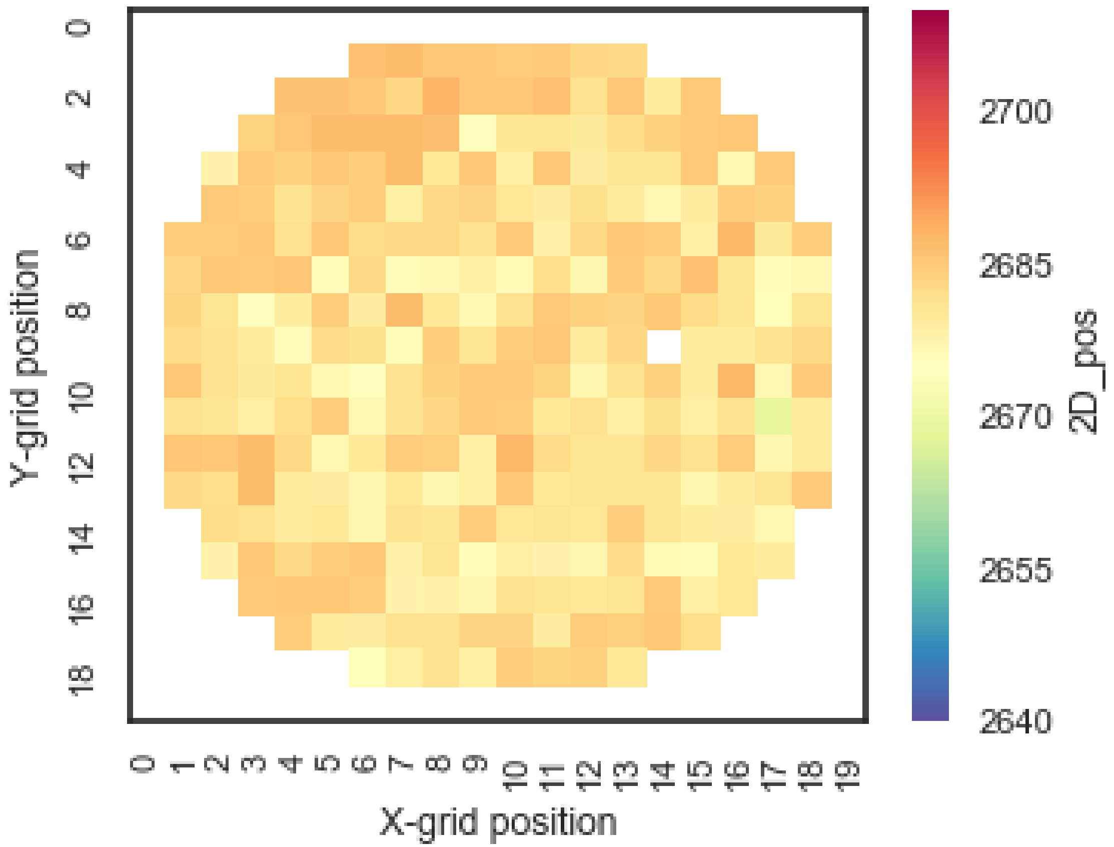

erik_pyr-0: G & 2D FWHM

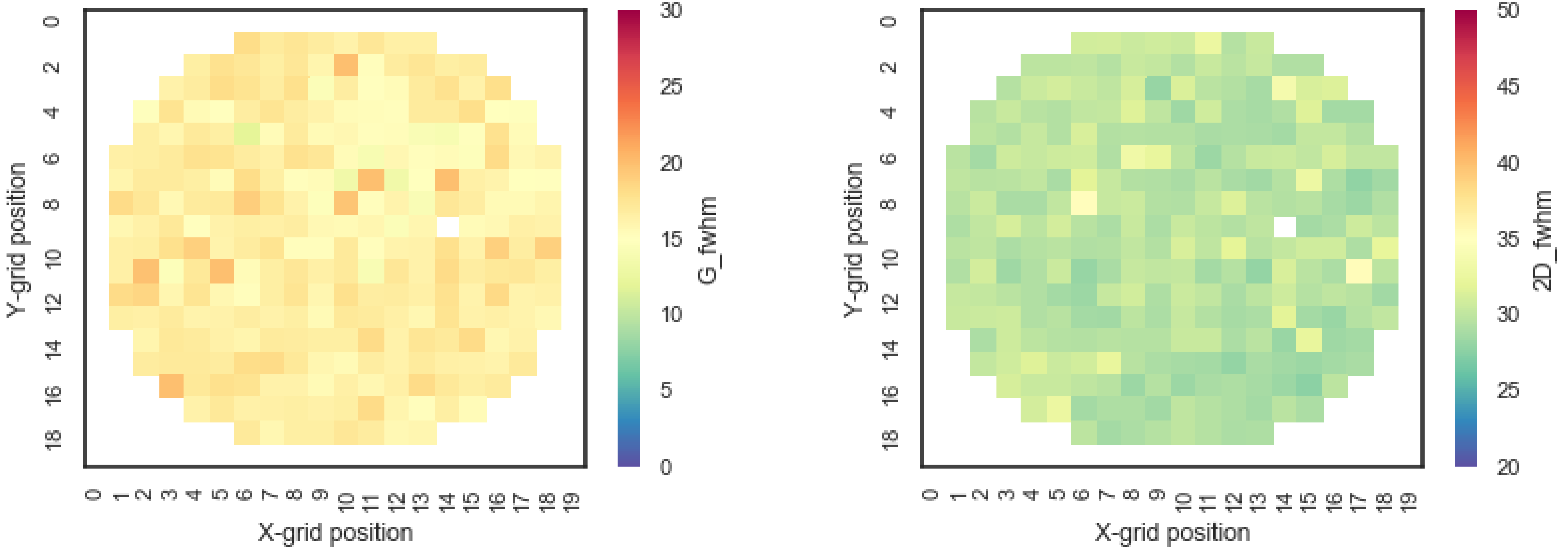

erik_pyr-0: G & 2D Intensity Ratios

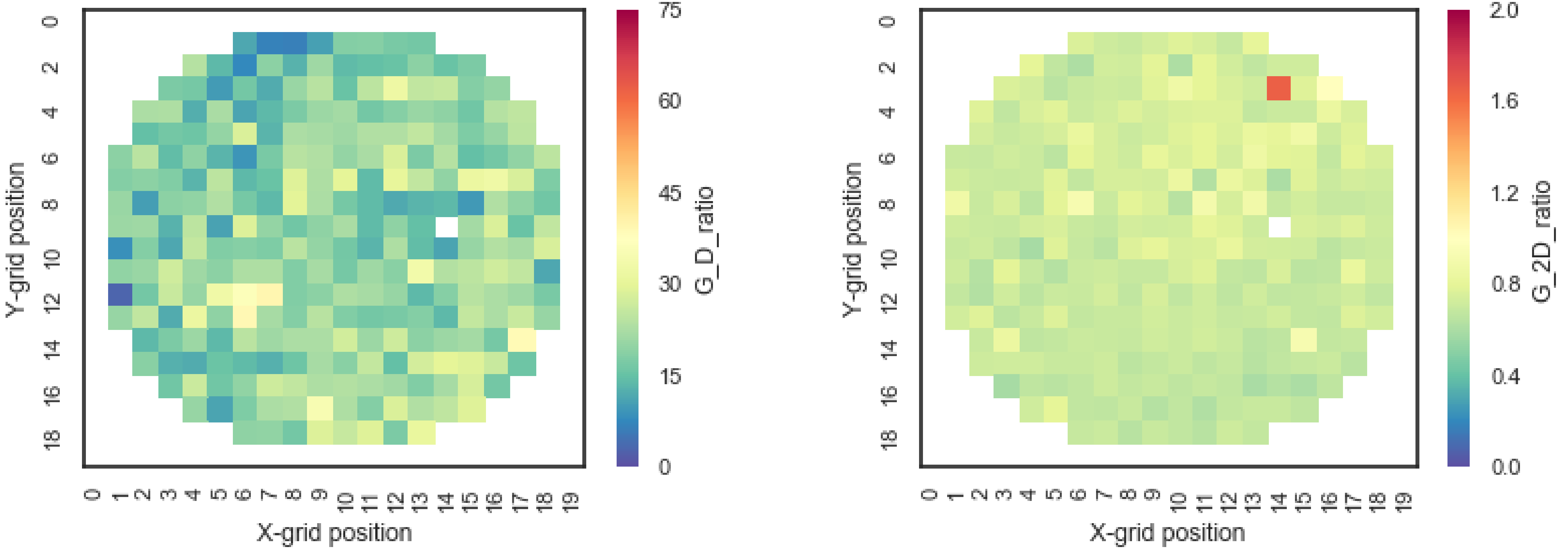

Strain and doping curves

Bare

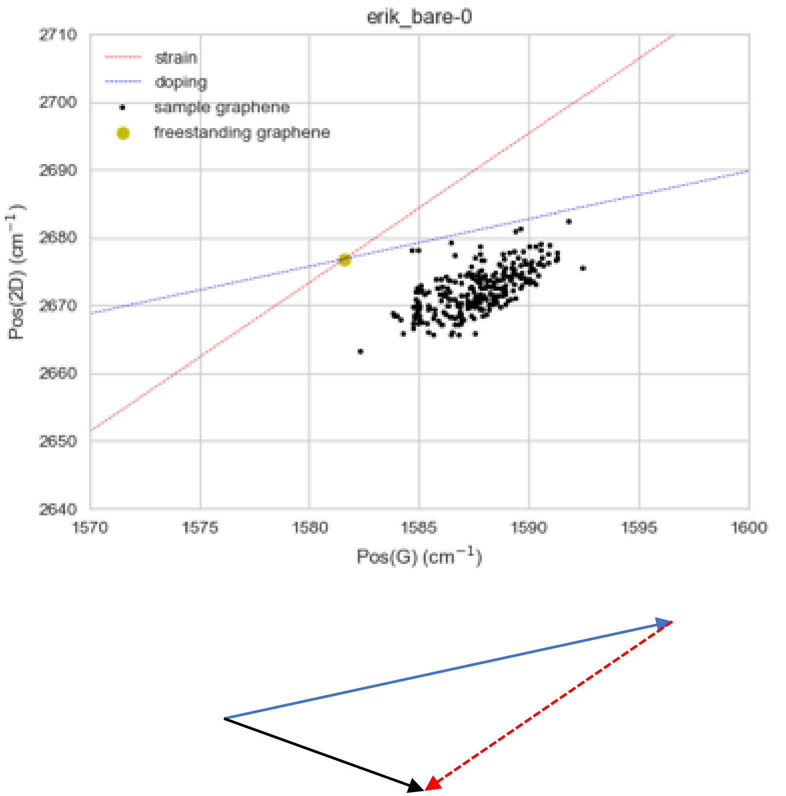

Tensile strain, high doping

OTS

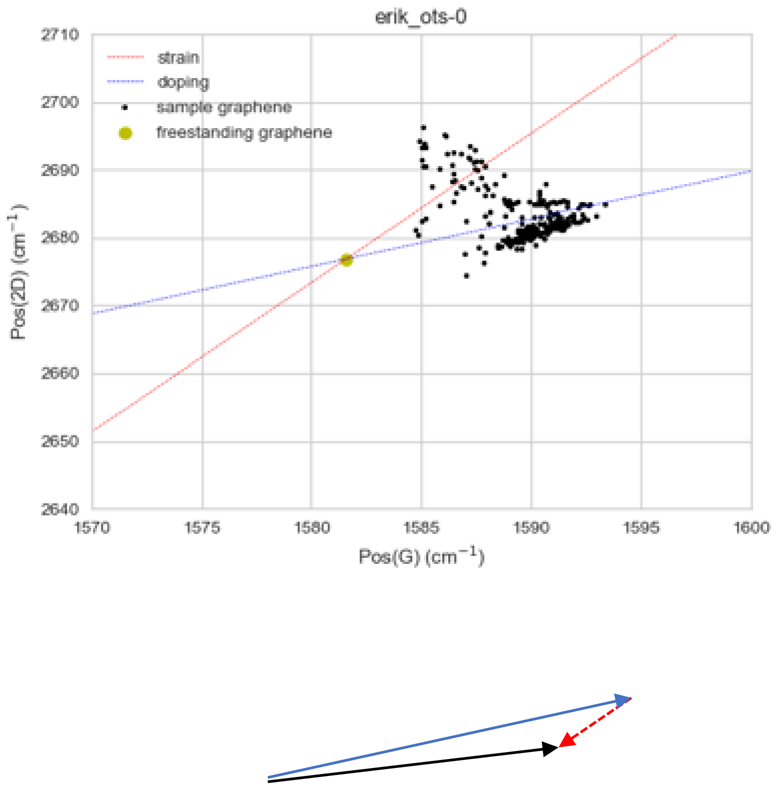

Reduced Tensile strain, reduced doping

Pyrene

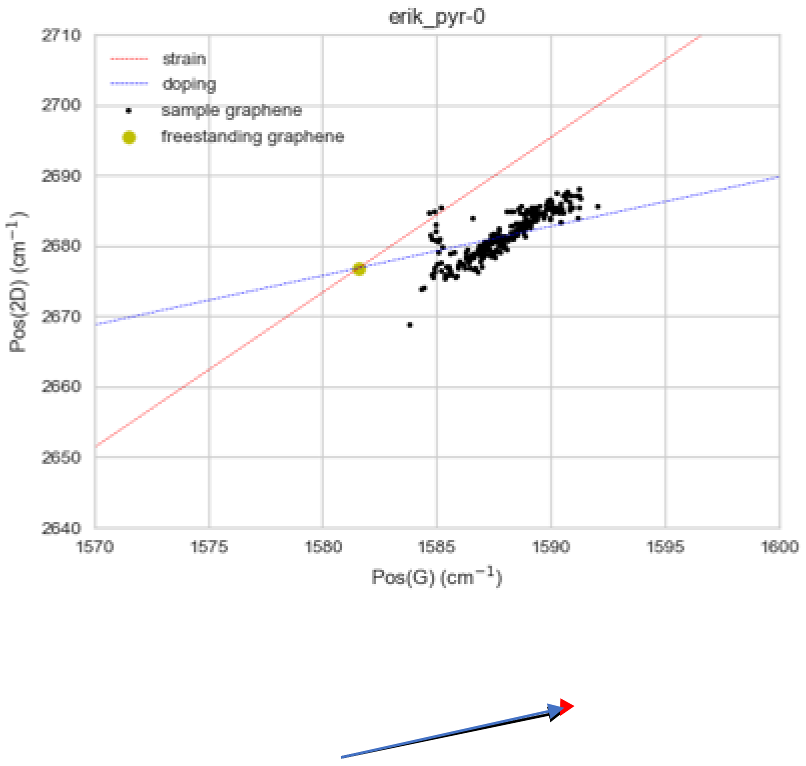

Zero strain, doping even more reduced

AFM data

AFM on wafers before transfers

Bare

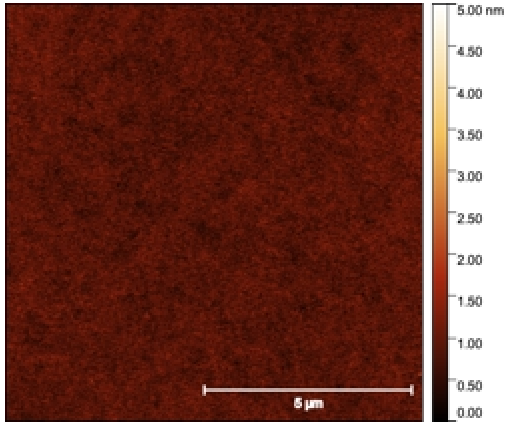

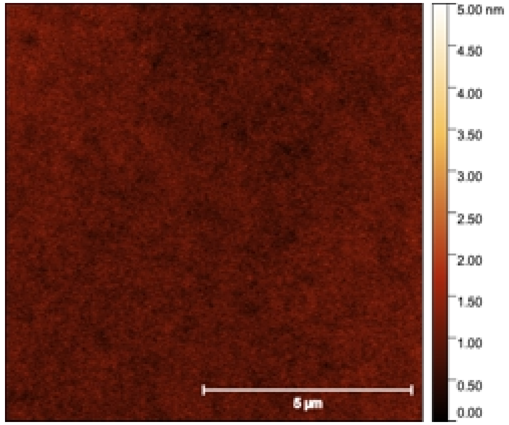

RMS roughness (Sq): 219,857 pm
Mean roughness (Sa): 174,785 pm

OTS

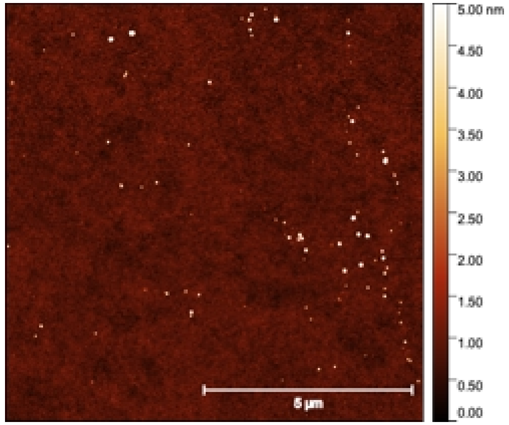

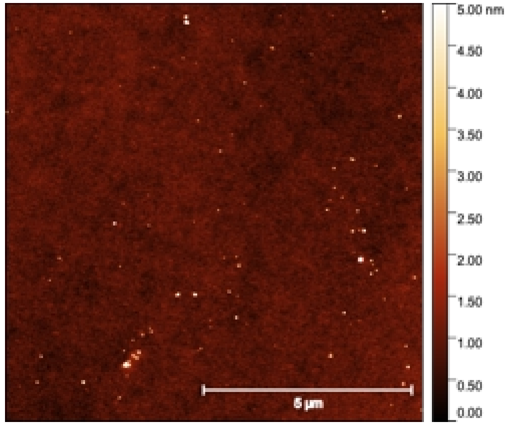

RMS roughness (Sq): 383,768 pm
Mean roughness (Sa): 166,329 pm

Pyrene

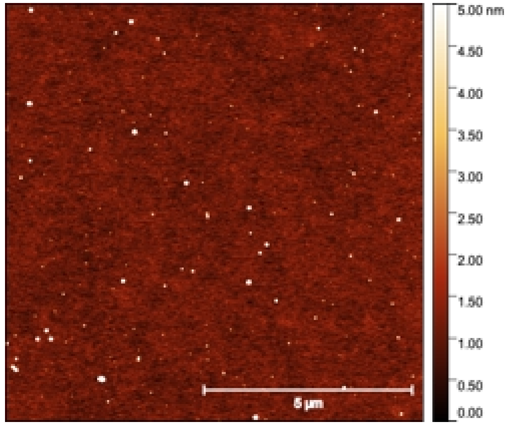

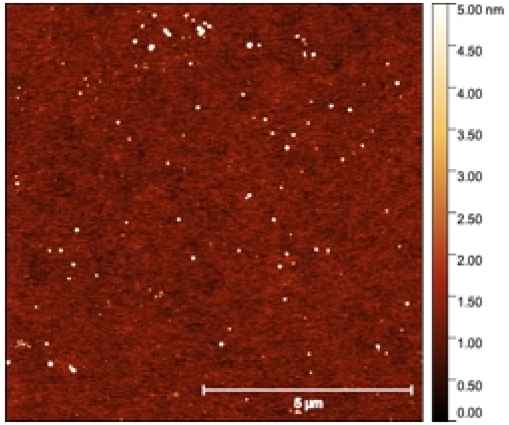

RMS roughness (Sq): 658,656 pm
Mean roughness (Sa): 250,672 pm

Graphene on Bare after transfer and polymer removal

bottom | center | top

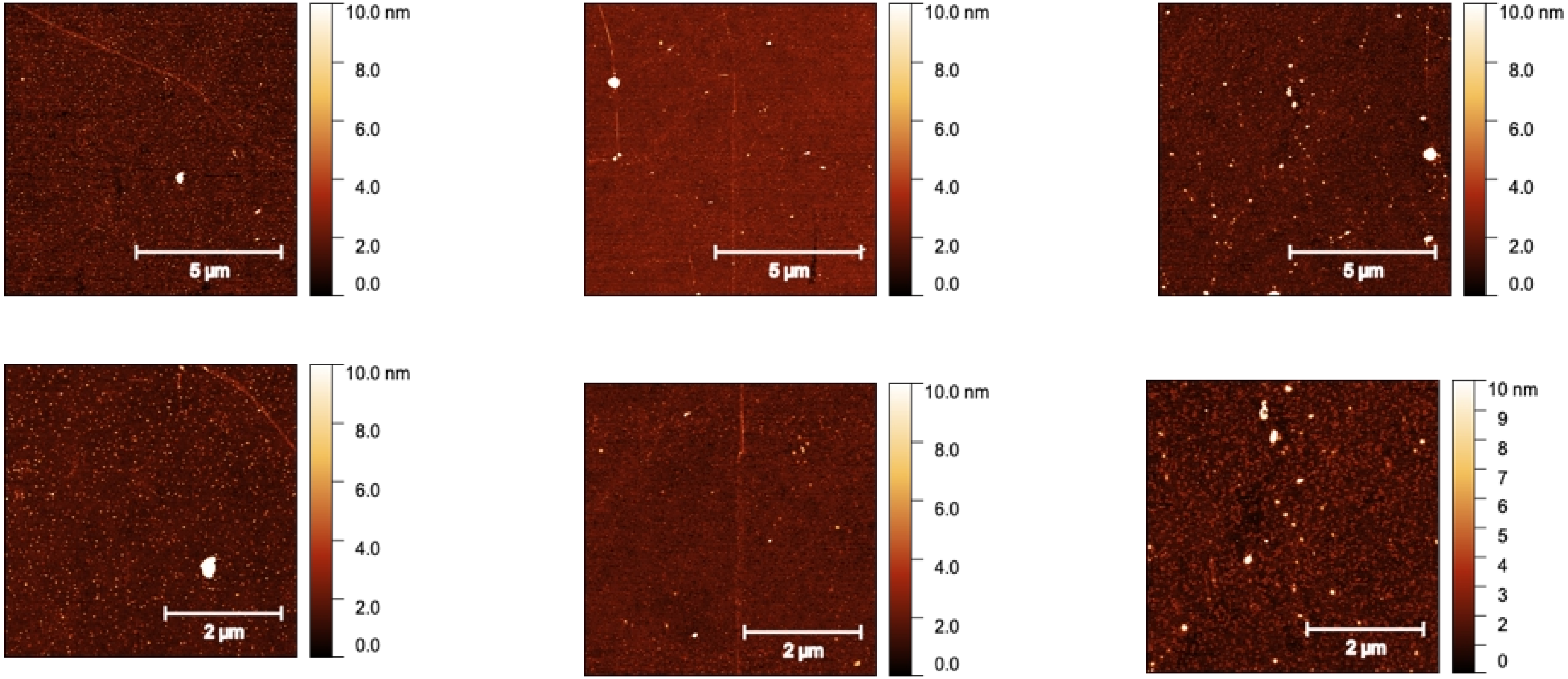

bottom
RMS roughness (Sq): 888,906 pm
Mean roughness (Sa): 466,128 pm

center
RMS roughness (Sq): 1,09125 nm
Mean roughness (Sa): 0,33533 nm

top
RMS roughness (Sq): 1,33134 nm
Mean roughness (Sa): 0,47457 nm

Graphene on OTS after transfer and polymer removal

bottom | center | top

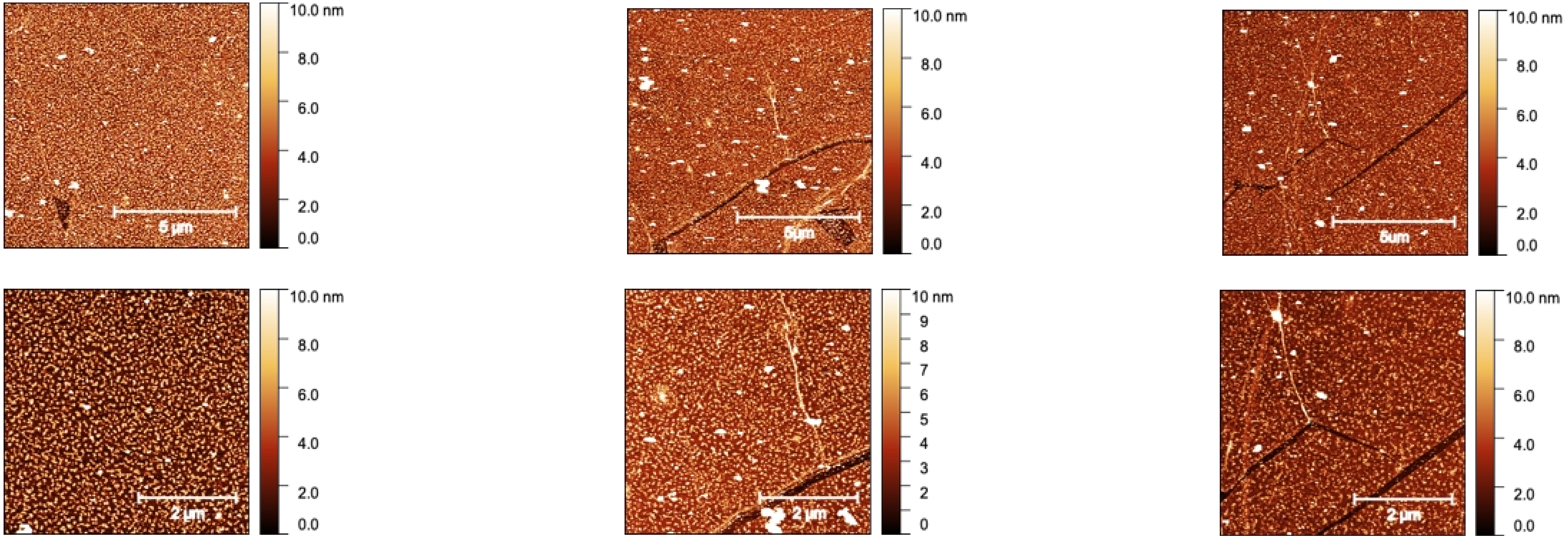

RMS roughness (Sq): 3,37539 nm
Mean roughness (Sa): 2,10155 nm

RMS roughness (Sq): 4,52076 nm
Mean roughness (Sa): 2,03865 nm

RMS roughness (Sq): 3,12640 nm
Mean roughness (Sa): 1,49301 nm

Graphene on Pyrene after transfer and polymer removal

bottom

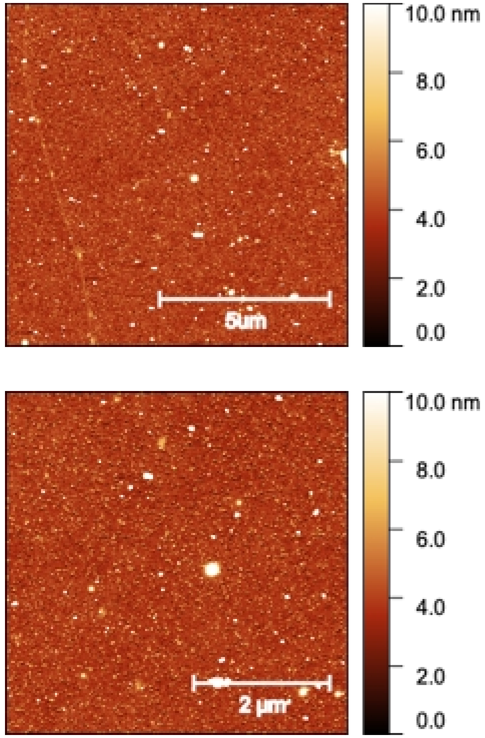

center

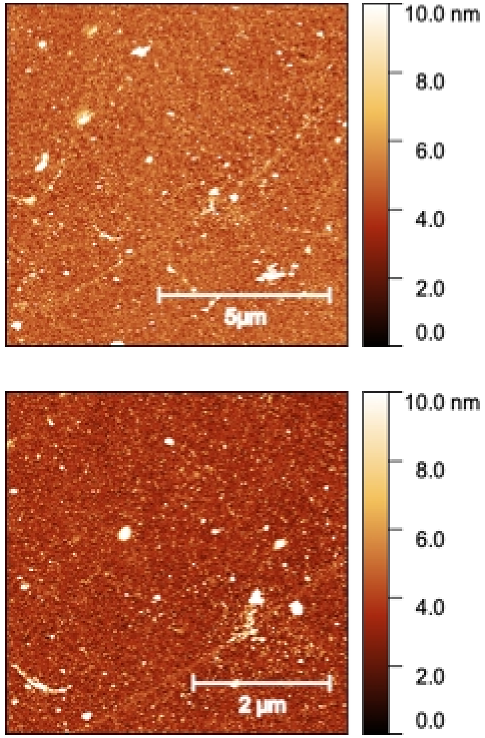

top

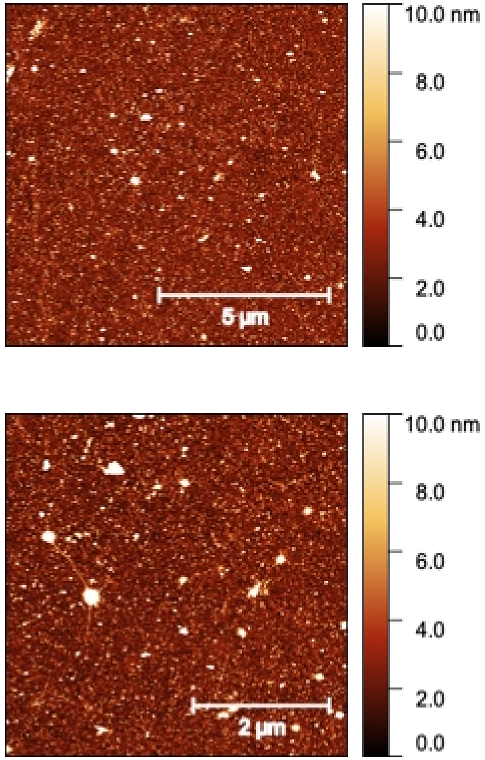

RMS roughness (Sq): 1,62348 nm
Mean roughness (Sa): 0,62964 nm

RMS roughness (Sq): 2,87724 nm
Mean roughness (Sa): 0,99592 nm

RMS roughness (Sq): 2,10077 nm
Mean roughness (Sa): 1,05717 nm
