# Supplementary material for: Wafer-scale robust graphene electronics under industrial processing conditions
Source: Chem Commun (Camb). 2026 Jul 3;62(57):14225–9. doi: 10.1039/d6cc01037g (PMC13330647; doi:10.1039/d6cc01037g)
Supplement: CC-062-D6CC01037G-s003 [file CC-062-D6CC01037G-s003.pdf]

Supplementary Information (SI) for Chemical Communications.
This journal is © The Royal Society of Chemistry 2026

Supplementary Information for

# Wafer-scale robust graphene electronics under industrial processing conditions

E. P. van Geest[1,2], B. Can,[1,2] M. Makurat,[1,2], C. Maheu[3,4], H. Sezen[4], M.D. Barnes[5], D. Bijl[5], M. Buscema[5], S. Shankar[5], D. J. Wehenkel[5], R. van Rijn[5], J.P. Hofmann[4], J. M. van Ruitenbeek[2]*, G. F. Schneider[1]*

[1] *Leiden Institute of Chemistry, Leiden University, Einsteinweg 55, 2333CC Leiden, The Netherlands*

[2] *Leiden Institute of Physics, Leiden University, Kamerlingh Onnes Laboratory, Niels Bohrweg 2, 2333 CA Leiden, The Netherlands*

[3] *Nantes Université, CNRS, Institut des Matériaux de Nantes Jean Rouxel, IMN, F-44000 Nantes, France.*

[4] *Surface Science Laboratory, Department of Materials- and Geosciences, Technical University of Darmstadt, Peter-Grünberg-Straße 4, 64287 Darmstadt, Germany*

[5] *Applied Nanolayers B.V. Feldmannweg 17, 2628 CD Delft, The Netherlands*

* to whom correspondence should be addressed:

g.f.schneider@chem.leidenuniv.nl

ruitenbeek@physics.leidenuniv.nl

Keywords: graphene device, molecular glue, pyrene, surface chemistry, device engineering

## Materials and methods

Chemicals were purchased at Sigma Aldrich or Brunschwig Chemie and used without further purification unless stated otherwise. Monolayer graphene on copper was purchased from Graphenea. Graphene on copper was spin-coated with poly(methyl methacrylate) (PMMA, 6% in anisole, Allresist GmbH, AR-P 662.06; 4000 rpm for 60 s), heated at 85°C for 10 minutes, then back-etched in oxygen plasma (0.30 mbar, 100 W, 2 minutes) and transferred on a cleaned wafer (sonication in acetone for 5 min, then rinsed with acetone, MilliQ and isopropanol (IPA), then treated with $O_2$ plasma (0.30 mbar, 100 W, 2 minutes).

Wafer surface functionalization was performed using silane chemistry. First, bare wafer pieces or an intact 4-inch wafer were cleaned by sonication in acetone for 5 minutes, then rinsed with acetone, ultra-pure water and IPA, then blown dry with pressurized nitrogen. Next, the wafer was treated with $O_2$ plasma (0.30 mbar, 100 W, 2 minutes), and directly transferred into a solution of 5vol% of the corresponding silane in 96% ethanol in the case of aminopropyl-triethoxysilane (APTES), hexamethyldisilazane (HMDS), and phenyltriethoxysilane (PHEN); for octadecyltrimethoxysilane (OTS) a 5vol% solution of the silane in hexane was used. Wafers were kept in solution to react overnight at room temperature. Then, they were taken out of the silane solution and sonicated in acetone for 5 minutes, rinsed with acetone, ultra-pure water and IPA, and blown dry with pressurized nitrogen. The BARE wafers used as reference underwent the same procedure, except that the wafers were immersed in 96% ethanol without any additives.

To produce PYRENE-coated wafers, clean APTES-modified wafer pieces were immersed in a solution of hexafluorophosphate azabenzotriazole tetramethyl uronium (HATU, 22 mM, 1.5 eq, 85 mg) and 1-pyrenebutyric acid (15 mM, 1.0 eq, 43 mg) in 10 ml DMF that was basified using 4 drops of triethylamine (TEA). For an intact 4-inch wafer, all amounts were multiplied by a factor 5. The wafers were kept in solution to react for three days at room temperature. Afterwards, the wafers were removed from solution, sonicated in acetone for 5 minutes, rinsed with acetone, ultra-pure water and IPA, and blown dry with pressurized nitrogen.

Graphene was transferred using a PMMA-assisted transfer method by etching copper with an ammonium persulfate solution (0.2 M in ultra-pure water) and rinsing the PMMA-graphene film by transferring it in three MilliQ baths consecutively, after which this film was transferred on the wafer by bottom fishing the floating film from below. Next, water was allowed to gently evaporate at 45°C; when water was evaporated, the coated wafer was heated at 150°C for 15 minutes. After successful transfer, the PMMA layer was removed by immersing the wafer in acetone for 10 minutes, then rinsing gently with acetone, ethanol and isopropyl alcohol, and blowing the wafer dry with pressurized nitrogen.

For the industrial graphene experiments, CVD graphene was dry-transferred on intact, modified 4-inch $Si/SiO_2$ wafers (Prime grade, p-doped, 90 or 285 nm $SiO_2$ (dry), single-side polished, Siegert Wafer GmbH), which were cleaned by sonication in acetone, DI water, and isopropanol (5 minutes in each solvent) and blown dry with pressurized nitrogen.

Oxygen plasma was generated using a capacitively coupled plasma system with radio-frequency of 40 kHz and 200 W power from Diener electronic (Femto), employed at room temperature. Spin coating was performed using a POLOS SPIN150i tabletop spin coater.

## Characterization

Optical images were obtained using a Leica DM2700 M Brightfield microscope fitted with Leica MC120 HD camera. Contact angles measurements were performed using a Ramé-Hart 250 goniometer (Netcong, NJ) in combination with the DROPimage advanced v 2.8 software. AFM images were recorded on a JPK Nanowizard Ultra in intermittent contact mode at room temperature in air, using Olympus micro cantilevers (OMCL-A160TS-R3) with a nominal resonance frequency of 300 kHz. Raman spectra were recorded on a Witec Alpha500 R Raman spectrometer using a 532 nm laser at low power (0.23 mW) and a 100x objective (lateral resolution 200-300 nm). ThermoFisher's Escalab-250 was used for XPS measurements. A monochromic Al Kα X-ray source (hv = 1486.68 eV) with a 650 μm spot size was employed. The XPS measurement chamber has a higher vacuum level than $5 \times 10^{-10}$ mbar. The high-resolution core-levels and survey spectra were obtained at pass energies of 20 eV and 50 eV, respectively. All XP spectra were calibrated with C 1s set to 285.0 eV for adventitious carbon. Electrical characterization of devices was using two Keithley Sourcemeters - model 2450 (one for the electrical measurement and one to supply a gate voltage) and Kickstart V2 software.

## Supplementary Figures and Tables

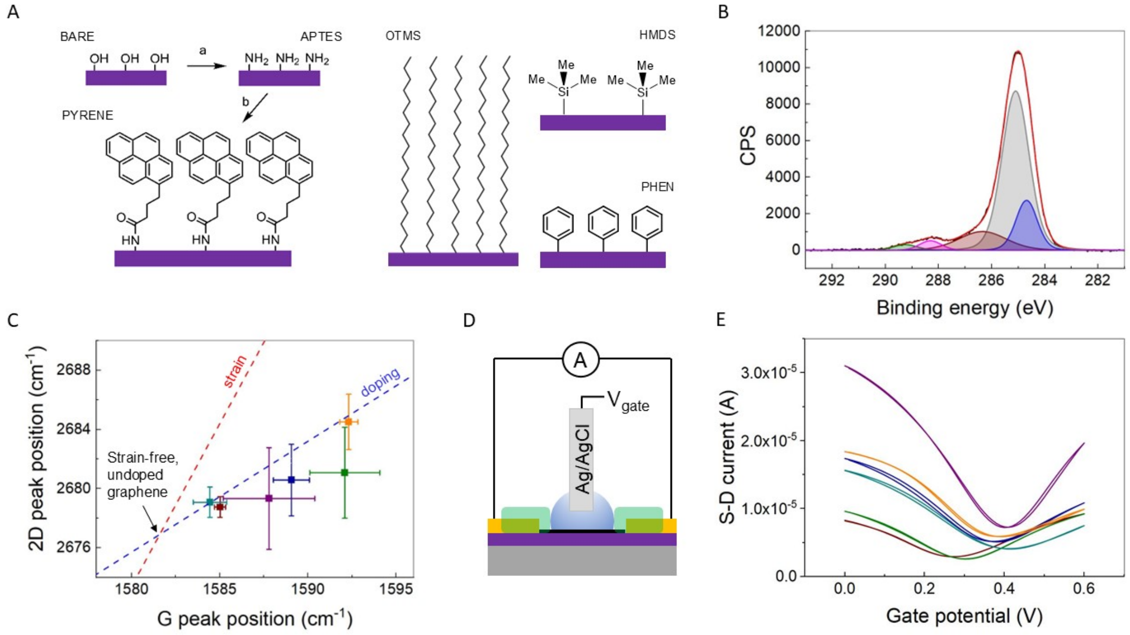


**Figure S1: Pyrene functionalization of silicon wafers and characterization of graphene on modified surfaces**. A) Synthesis scheme for the two-step functionalization, where (a) the $SiO_2$ wafer is immersed overnight in a 5v% solution of APTES in ethanol/water (96:4), then (b) 1-pyrenebutyric acid is reacted via a HATU peptide coupling in a $NEt_3$-basified DMF solution for three days. Both reactions are performed at room temperature. Reference surface modifications OTS, HMDS and PHEN are shown on the right hand side of the figure panel. B) Baseline-subtracted XPS spectrum of the pyrene-functionalized wafer, deconvolution of the C1s peak (CPS = counts per second). Interpretation: 284.7 eV (blue) = sp2 C 1s, 285.0 eV (grey) = sp3 C 1s, 286.3 eV (brown) = C-N (residual amine), 288.3 eV (pink) = N-C=O (amide), 289.3 eV (green) = O-C=O (residual carboxylic group). C) Deconvolution of the Raman (average) into the strain (red) and doping (blue) components of the vector. Each data point represents four independently functionalized samples (five spectra per sample). Error bars are generated from four independently functionalized wafers. The red and blue lines indicate zero strain and zero doping axes.[22] D) Schematic representation of the liquid-gated GFET. E) Typical $I_{SD}$ vs. $V_{gate}$ plots for liquid-gated GFETs (0.1M $LiClO_4$). $V_{gate}$ was swept ten times between 0 and 0.6 V at 0.01 $Vs^{-1}$, cycle 10 is displayed in the plot. Color code for all panels are: bare ($SiO_2$) = purple, OTS = red, APTES = green, HMDS = cyan, PHEN = blue and PYRENE = orange.

**Table S1: Contact angle of modified wafers and Raman analysis of graphene on the functionalized wafers.** Standard deviation in brackets. Hys. = hysteresis.

| **Surface** | **CA** | **AFM - roughness** | | **Raman - peak position** | | $I_{SD}$ ***vs.*** $V_{gate}$ | |
|---|---|---|---|---|---|---|---|
| | $\theta_c$ (°) | $R_a$ (pm) | RMS (pm) | G ($cm^{-1}$) | 2D ($cm^{-1}$) | $V_{CNP}$ (mV) | Hys. (mV) |
| Bare | 35 (7) | 157 (26) | 266 (66) | 1587.8 (2.6) | 2679.3 (3.4) | 379 (86) | 14 (44) |
| OTS | 92 (5) | 240 (55) | 529 (215) | 1585.0 (0.3) | 2678.7 (0.7) | 282 (30) | 12 (15) |
| APTES | 49 (7) | 203 (62) | 732 (665) | 1592.1 (2.0) | 2681.1 (3.1) | 303 (55) | 7 (12) |
| HMDS | 61 (7) | 194 (32) | 396 (80) | 1584.4 (1.0) | 2679.1 (1.0) | 377 (36) | 10 (12) |
| PHEN | 42 (2) | 305 (206) | 688 (577) | 1589.1 (1.0) | 2680.6 (2.4) | 353 (63) | 2 (9) |
| PYRENE | 69 (5) | 231 (66) | 565 (298) | 1592.3 (0.5) | 2684.5 (1.9) | 379 (50) | -8 (7) |

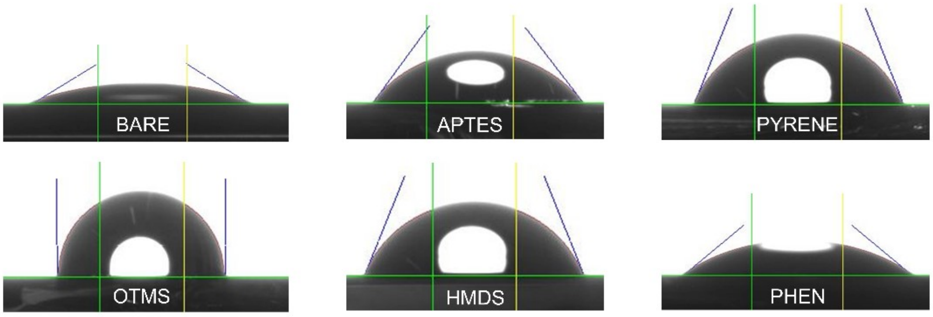


**Figure S2: Optical images of CA measurements**. Blue lines indicate contact angle measurement.

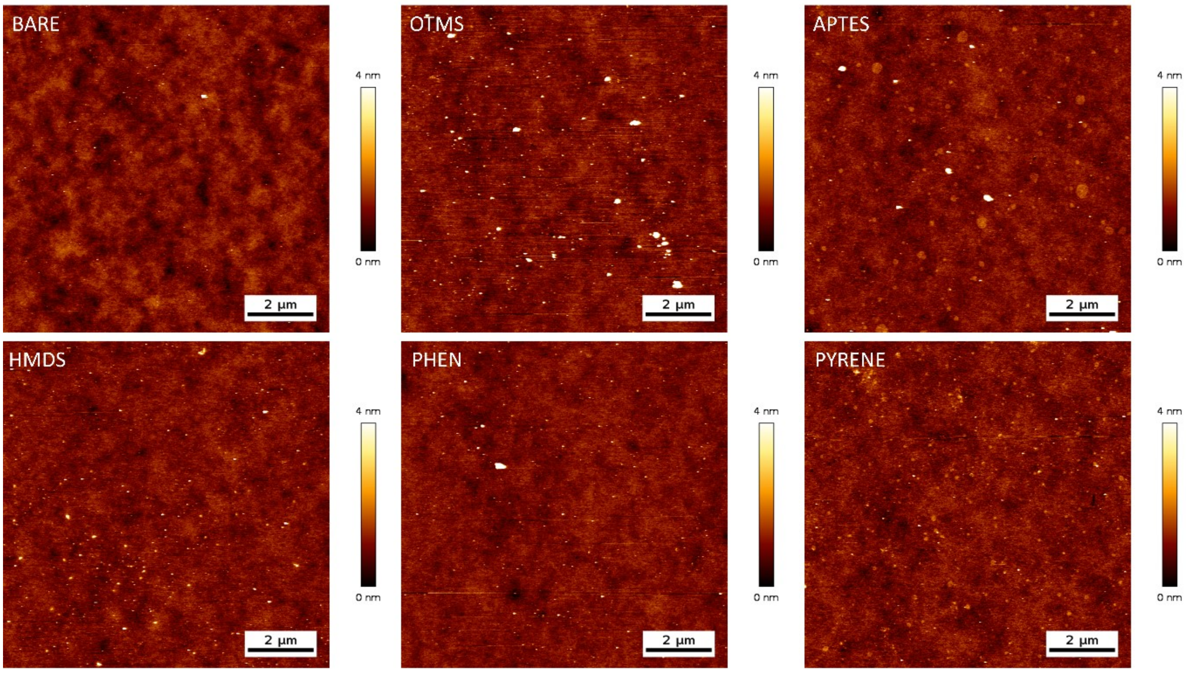


**Figure S3: AFM images of chemically modified wafers.**

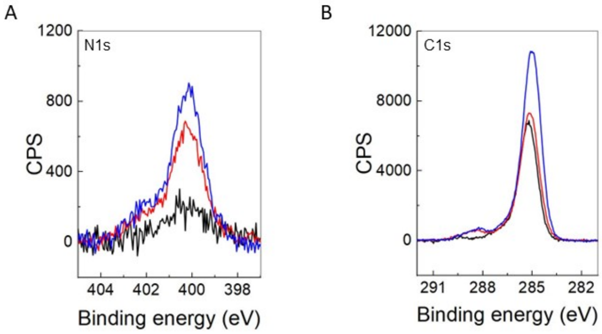


**Figure S4: Baseline-subtracted XPS spectra of bare, APTES, and pyrene-functionalized wafers**. N 1s (A) and C 1s (B) core level spectra for bare, APTES-, and pyrene functionalized wafer (black, red and blue respectively). CPS = counts per second.

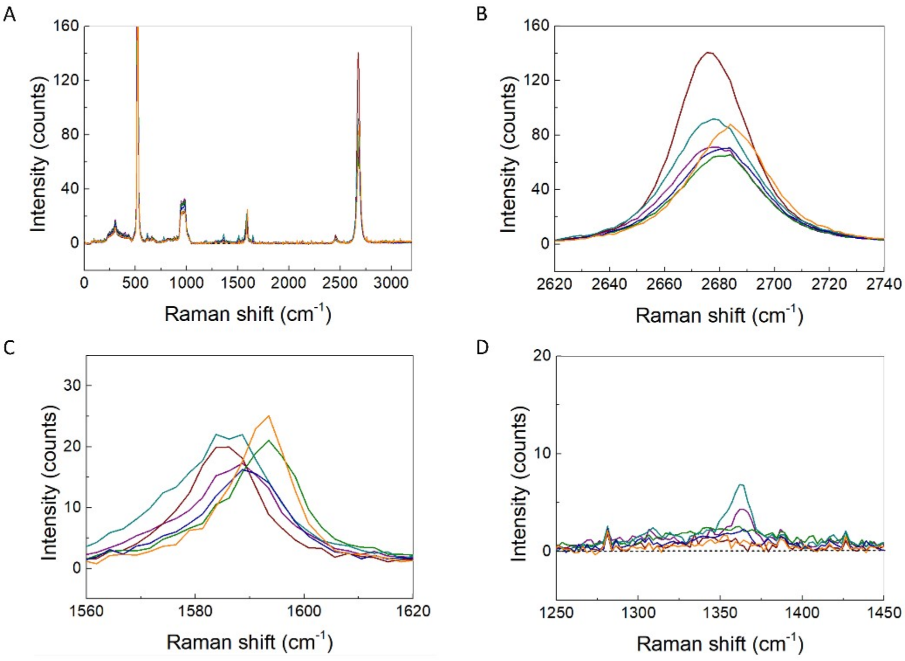


**Figure S5: Raman analysis of graphene on chemically modified wafers.** A) Full spectrum. B) Zoom on 2D peak region. C) Zoom on G peak region. D) Zoom on D peak region. Colour code: bare ($SiO_2$) = purple, OTMS = red, APTES = green, HMDS = cyan, PHEN = blue and PYRENE = orange.

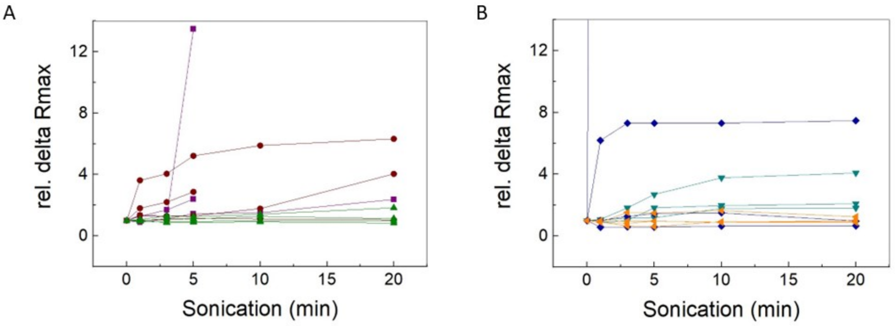


**Figure S6**: Individual values relative resistance variation ($R_{max,t}$ / $R_{max,0}$) for devices that were sonicated in acetone. Panel A displays BARE, OTMS and APTES, panel B displays HMDS, PHEN and PYRENE. Colour code: bare ($SiO_2$) = purple, OTMS = red, APTES = green, HMDS = cyan, PHEN = blue and PYRENE = orange.

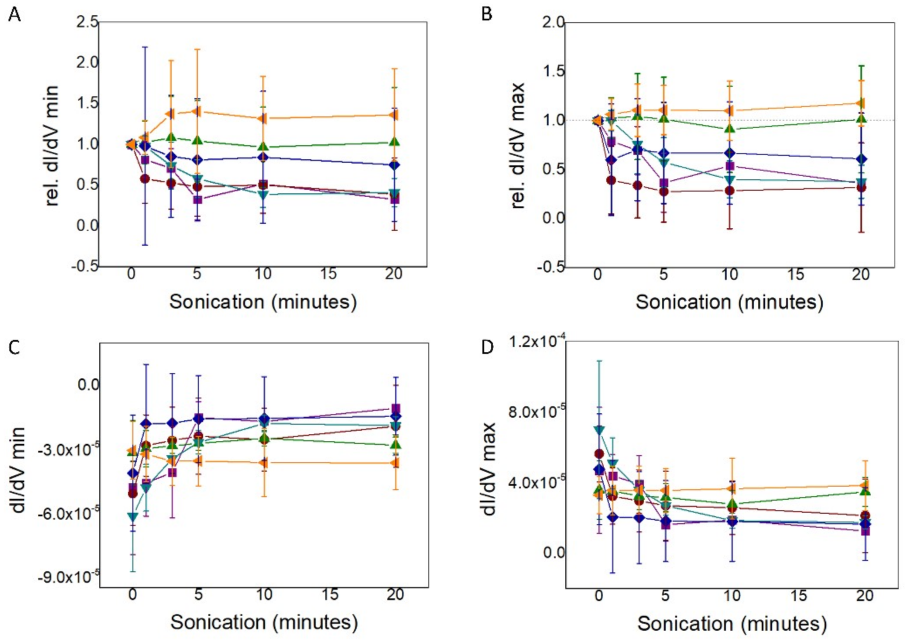


**Figure S7: Evolution of the minimum and maximum of *dI/dV*$_{gate}$ (absolute and relative to *dI/dV*$_{gate}$ at t = 0 min) during sonication**. A) relative minimum, B) relative maximum, C) absolute minimum, and D) absolute maximum of $dI/dV_{gate}$. Liquid gating performed using 0.1M $LiClO_4$, $V_{gate}$ was swept 10 times between 0 and 0.6V at 0.01Vs$^{-1}$, the data of the last cycle was used to construct the plots. Colour coding for all panels: bare ($SiO_2$) = purple, OTMS = red, APTES = green, HMDS = cyan, PHEN = blue and PYRENE = orange.

**Table S2: Liquid gating (*I* vs. $V_{gate}$) results for GFETs constructed on modified silicon wafers, and for GFETs after sonication (t = 20 min).** Standard deviation in brackets. $dI/dV_{t=20min}$ values are reported relative to $\Delta dI/dV_{t=0min}$. Different sets of GFETs were used for the graphene characterization and sonication experiments.

| | $I_{SD}$ vs. $V_{gate}$ - sonication | | | | | | | | |
|---|---|---|---|---|---|---|---|---|---|
| | Survival | | $\Delta R_{max}$ | $V_{CNP,}$ (mV) | | Hysteresis (mV) | | $\mu_{hole}$ (x10$^3$ cm$^2$V$^{-1}$s$^{-1}$) | |
| **Surface** | $n_{t=0}$ | $n_{t=20}$ | $R_{t=20}$ / $R_{t=0}$ | 0 min | 20 min | 0 min | 20 min | 0 min | 20 min |
| Bare | 4 | 1 | 2.38 (-) | 336 (54) | 309 (-) | 8 (8) | 37 (-) | 0.98 (0.61) | 0.41 (-) |
| OTS | 4 | 3 | 3.80 (2.6) | 286 (53) | 241 (84) | 12 (3) | 22 (15) | 1.37 (0.66) | 0.64 (0.78) |
| APTES | 4 | 4 | 1.19 (0.4) | 272 (73) | 312 (53) | 11 (11) | 7 (7) | 0.82 (0.52) | 0.67 (0.28) |
| HMDS | 4 | 3 | 2.66 (1.2) | 333(24) | 342 (17) | 19 (7) | 35 (11) | 1.52 (0.53) | 0.51 (0.23) |
| PHEN | 4 | 3 | 3.03 (3.8) | 319 (96) | 283 (21) | 0 (7) | 2 (1) | 1.26 (0.99) | 0.54 (0.72) |
| PYRENE | 4 | 4 | 1.01 (0.2) | 279 (38) | 330 (20) | 0 (2) | 2 (16) | 0.86 (0.39) | 1.04 (0.41) |

**Table S3: Surface characterization of modified 4-inch wafers for industrial graphene application.** Average values are tabulated, standard deviation indicated in brackets.

| **Surface** | **CA** | **AFM before transfer** | | **AFM after transfer** | | **Raman - peak position** | |
|---|---|---|---|---|---|---|---|
| | $\theta_c$ (°) | Ra (pm) | RMS (pm) | Ra (pm) | RMS (pm) | G (cm$^{-1}$) | 2D (cm$^{-1}$) |
| Bare | 42 (-) | 175 (-) | 220 (-) | 425 (78) | 1104 (221) | 1587.7 (1.8) | 2672.2 (3.3) |
| OTS | 91 (5) | 166 (-) | 384 (-) | 1878 (335) | 3674 (744) | 1590.0 (1.9) | 2683.2 (3.8) |
| PYRENE | 69 (3) | 251 (-) | 659 (-) | 894 (231) | 2200 (633) | 1588.1 (1.7) | 2681.7 (3.3) |

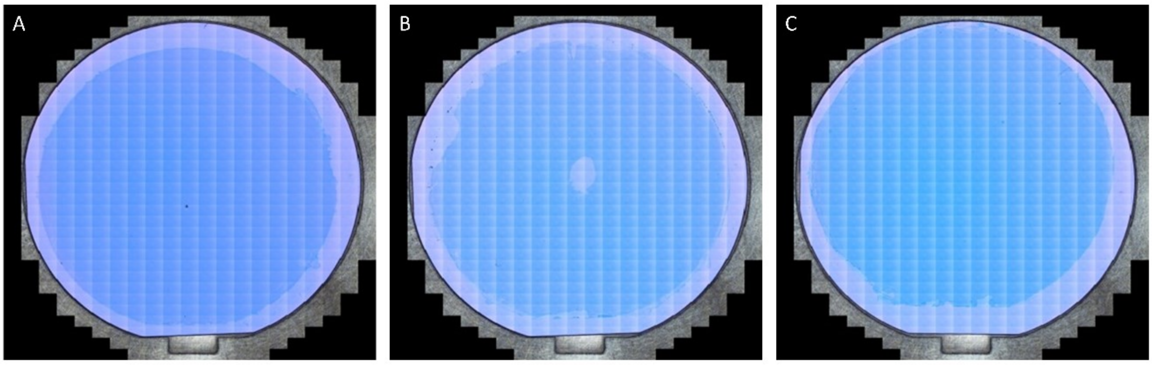


**Figure S8: Dry-transferred graphene on 4-inch modified wafers.** Optical images for bare (A), OTS- (B) and PYRENE-functionalized (C) wafers.

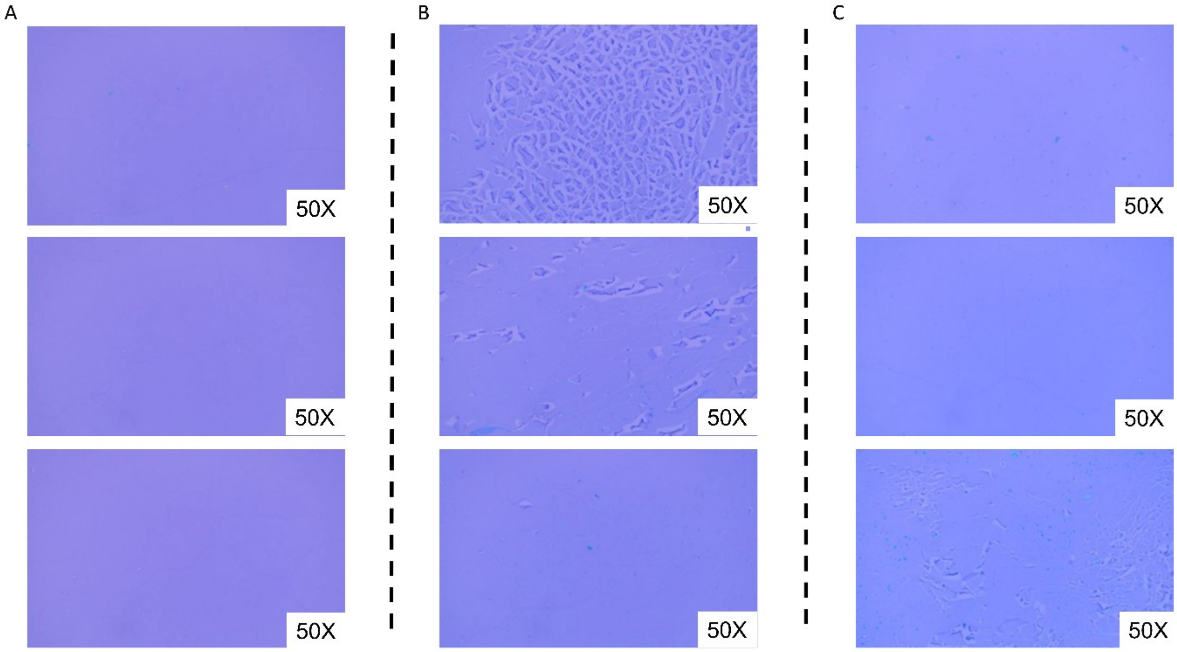


**Figure S9:** Zoomed in optical images (on three randomly chosen locations) for graphene on bare (A), OTS (B) and PYRENE (C) modified wafers on 4-inch wafer scale.

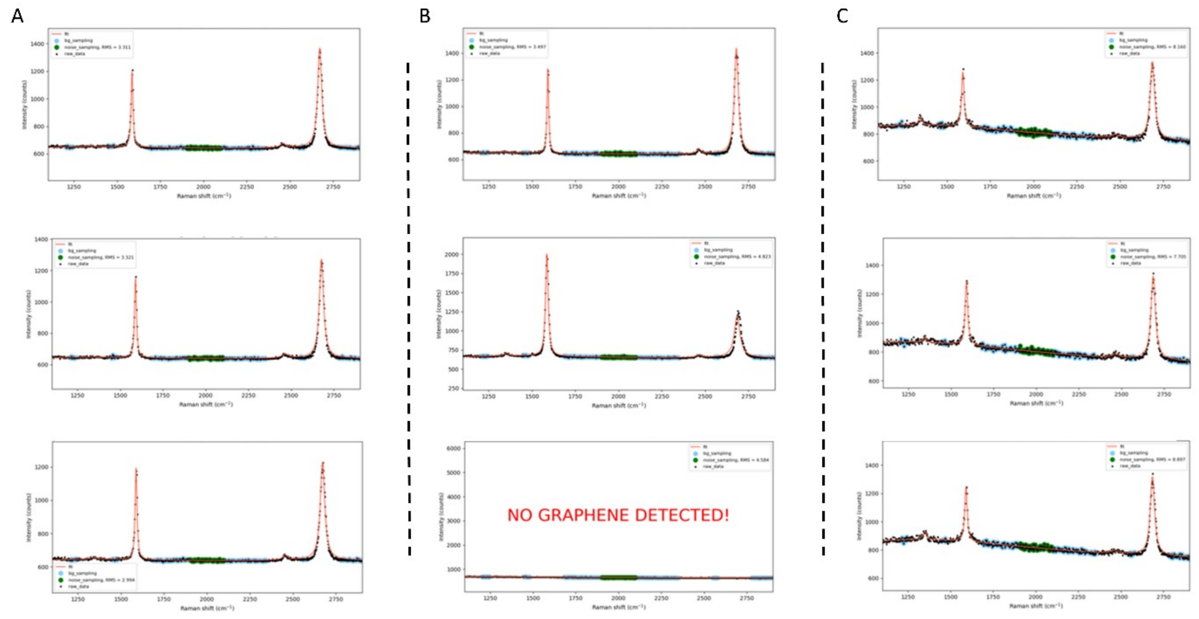


**Figure S10**: Raman spectra (on three randomly chosen locations) for graphene on BARE (A), OTS (B) and PYRENE (C) wafers on 4-inch wafer scale.

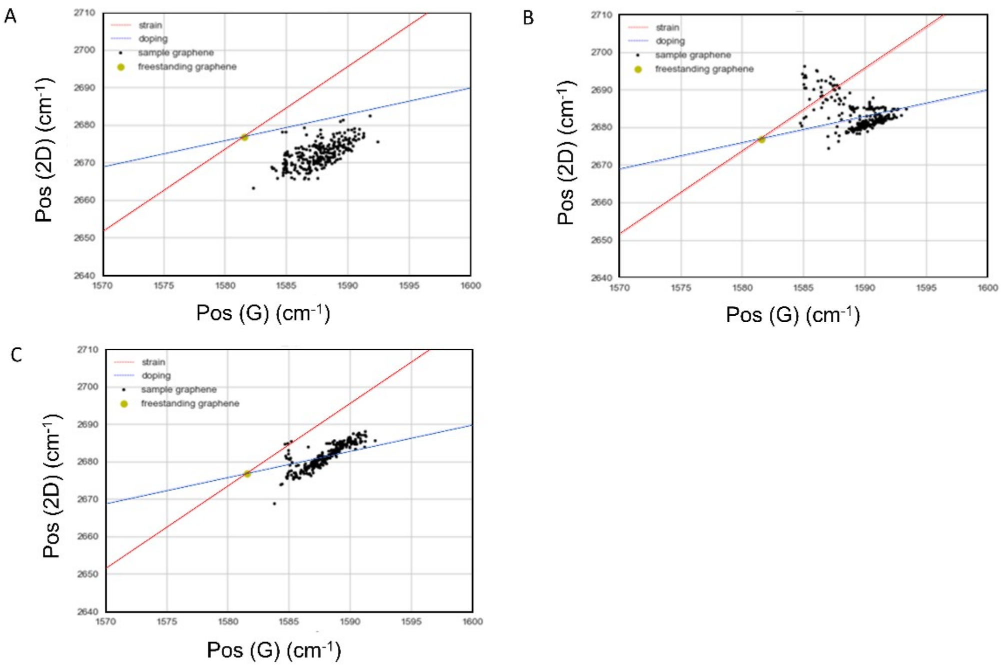


**Figure S11**: Raman spectroscopy G vs 2D scatterplot for graphene on BARE (A), OTS (B) and PYRENE (C) wafers on 4-inch wafer scale.

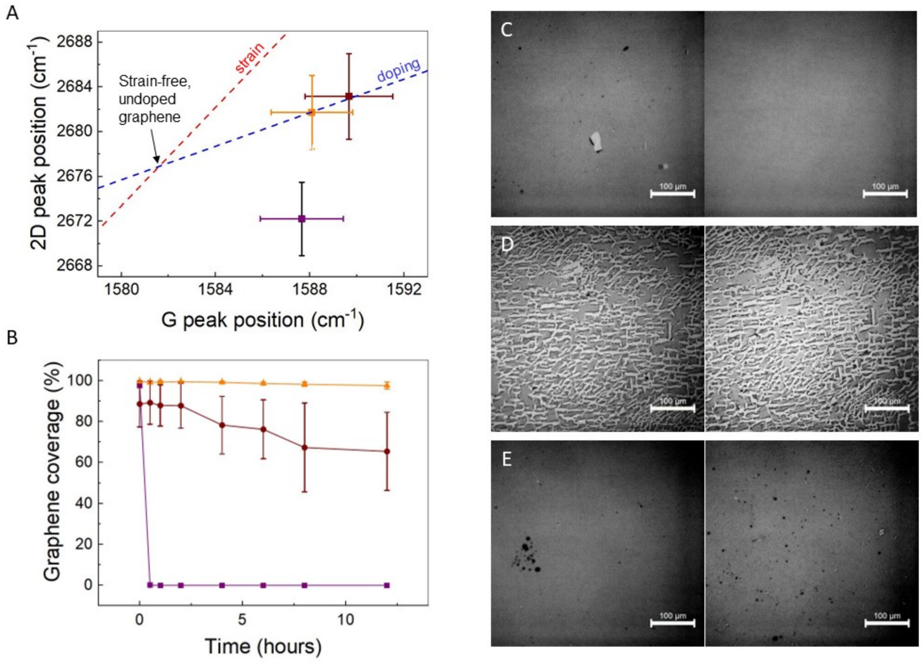


**Figure S12:** Full wafer (4”) scale dry transfer of graphene on modified wafers. A) Raman analysis of graphene on functionalized wafers (bare = purple, OTS = red, PYRENE = orange). Red and blue diagonal lines represent the axes for strain and doping. Deconvolution of the Raman (average) into the strain (red) and doping (blue) components of the vector. B) Graphene coverage vs. time for samples immersed in KOH (0.5M in water, room temperature), for graphene on BARE, OTS and PYRENE wafers (purple, red, and orange respectively). Samples were optically inspected (three samples for each functionalization, four photos per sample) and delamination was determined as the percentage of graphene coverage in the centre of the image (a circle with ⌀ = 150 µm). C-E) Contrast-enhanced optical images (magnification x20) for graphene on bare (C), OTS (D), and PYRENE (E) wafers immersed in KOH for 0 and 12h (left and right, respectively). The tears in the right image of (E) indicate that graphene has small damages but has not delaminated.

**Table S4:** Full wafer-scale graphene on modified surfaces - characterization. Graphene coverage values tabulated for 12h in 0.5M KOH, 12h in glacial acetic acid (AA), and 18h in pure NMP. Average values are tabulated, standard deviation indicated in brackets.

| **Surface** | **Raman - peak position** | | **Graphene coverage** | | |
|---|---|---|---|---|---|
| | G ($cm^{-1}$) | 2D ($cm^{-1}$) | KOH (%) | AA (%) | NMP (%) |
| Bare | 1587.7 (1.8) | 2672.2 (3.3) | 1 (0) | 30 (41) | 3 (3) |
| OTS | 1590.0 (1.9) | 2683.2 (3.8) | 65 (19) | 82 (16) | 22 (18) |
| PYRENE | 1588.1 (1.7) | 2681.7 (3.3) | 98 (2) | 99 (0) | 99 (1) |

**Table S5: Raman analysis of graphene on modified wafers on 4-inch wafer scale.** Averages shown of spectra data that was suitable for fitting (indicated as #scans showing graphene). FWHM = full-width half maximum.

| | **BARE** | | **OTS** | | **PYRENE** | |
|---|---|---|---|---|---|---|
| **Parameter** | **Average** | **σ** | **Average** | **σ** | **Average** | **σ** |
| Total Raman scans in measurement | 276 | N/A | 276 | N/A | 276 | N/A |
| Number of scans showing graphene | 274 | N/A | 261 | N/A | 274 | N/A |
| Percentage of scans with graphene | 99% | N/A | 94% | N/A | 99% | N/A |
| D position | 1343.36 | 5.5 | 1347.67 | 4.8 | 1347.44 | 5.1 |
| D intensity | 50.34 | 669 | 52.59 | 590 | 26.46 | 10 |
| D FWHM | 25.5 | 10 | 23.16 | 8 | 32.85 | 8 |
| G position | 1587.66 | 1.8 | 1589.66 | 1.9 | 1588.09 | 1.7 |
| G intensity | 622.78 | 138 | 675.97 | 523 | 490.3 | 44 |
| G FWHM | 11.75 | 1.6 | 12.4 | 2.6 | 16.54 | 1.2 |
| 2D position | 2672.21 | 3.3 | 2683.15 | 3.8 | 2681.73 | 3.3 |
| 2D intensity | 638.74 | 67 | 757.78 | 310 | 677.43 | 51 |
| 2D FWHM | 30.45 | 1.6 | 28.26 | 3.3 | 29.87 | 1.1 |
| G/D Ratio | 77.1 | 38 | 60.45 | 39 | 20.33 | 6 |
| G/2D Ratio | 0.98 | 0.2 | 0.93 | 0.5 | 0.73 | 0.1 |

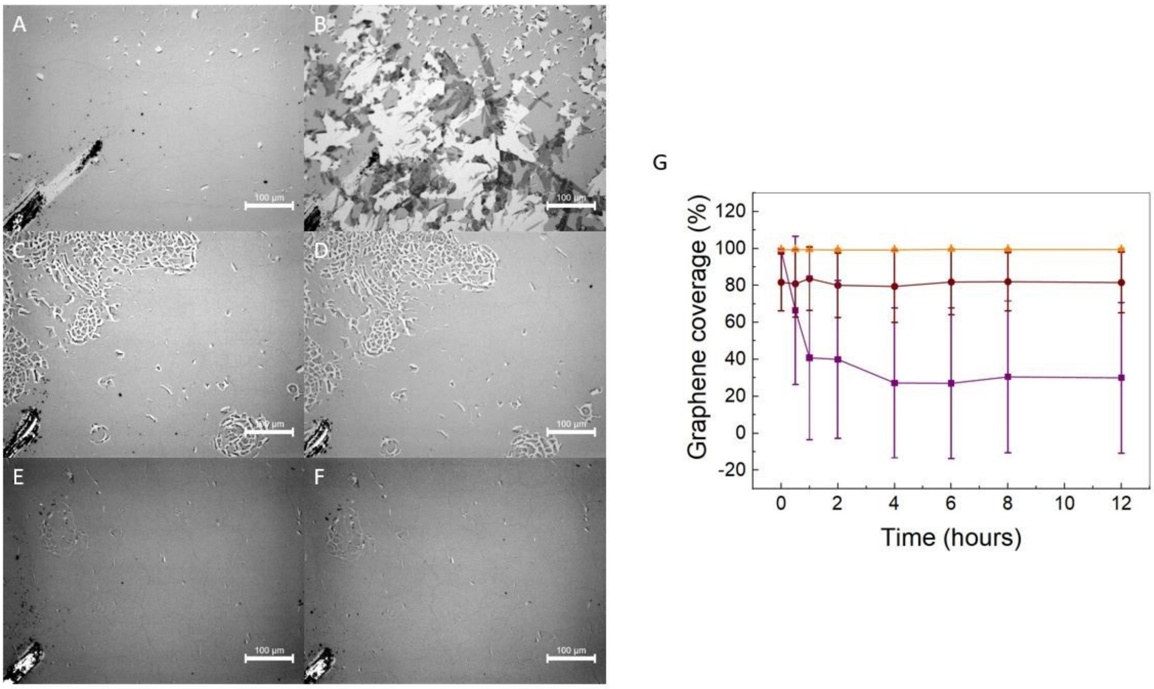


**Figure S13: Visually enhanced optical images from graphene on modified wafers, immersed in glacial acetic acid at room temperature**. BARE wafers before (A) and after 12 h (B), OTS wafers before (C) and after 12 h (D), and PYRENE wafers before (E) and after 12 h (F) immersion in acetic acid. G) Delamination vs. time for samples immersed in glacial acetic acid, for graphene on BARE, OTS and PYRENE wafers (purple, red, and orange respectively). Samples were optically inspected (three samples for each functionalization, four photos per sample) and delamination was determined as the percentage of area exposed in the center of the image (a circle with ⌀= 150 µm).

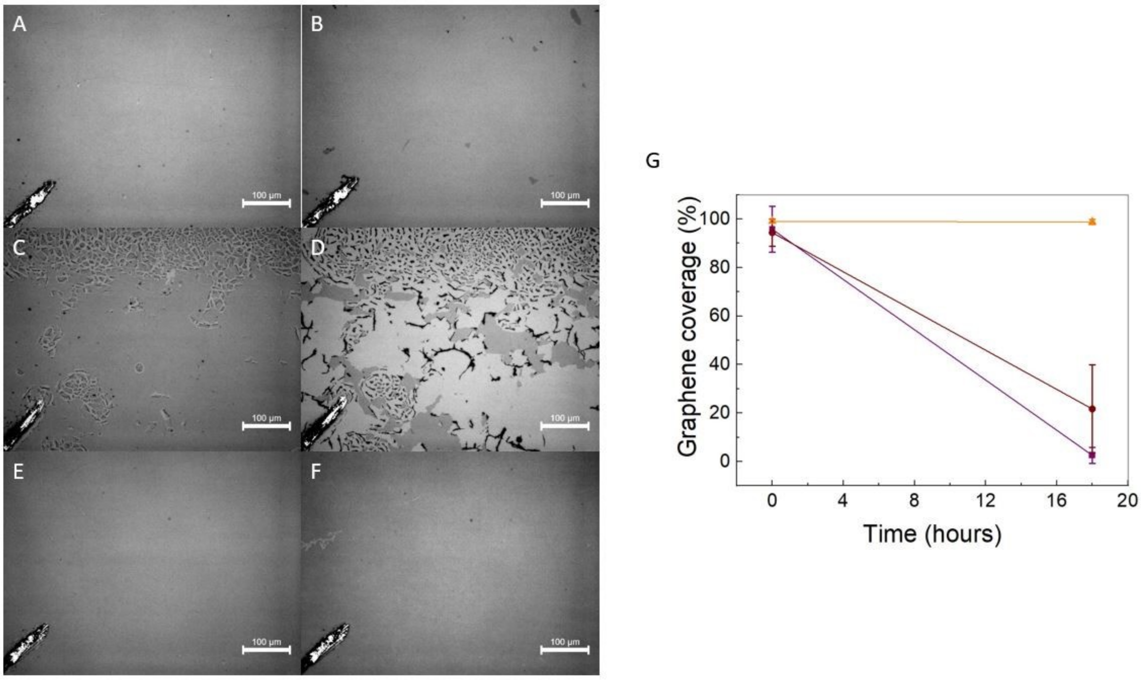


**Figure S14: Visually enhanced optical images from graphene on modified wafers, immersed in N-methyl pyrrolidine (NMP) at room temperature**. BARE wafers before (A) and after 12 h (B), OTS wafers before (C) and after 12 h (D), and PYRENE wafers before (E) and after 12 h (F) immersion in NMP. G) Delamination vs. time for samples immersed in NMP, for graphene on BARE, OTS and PYRENE wafers (purple, red, and orange respectively). Samples were optically inspected (three samples for each functionalization, four photos per sample) and delamination was determined as the percentage of area exposed in the center of the image (a circle with ⌀ = 150 μm).

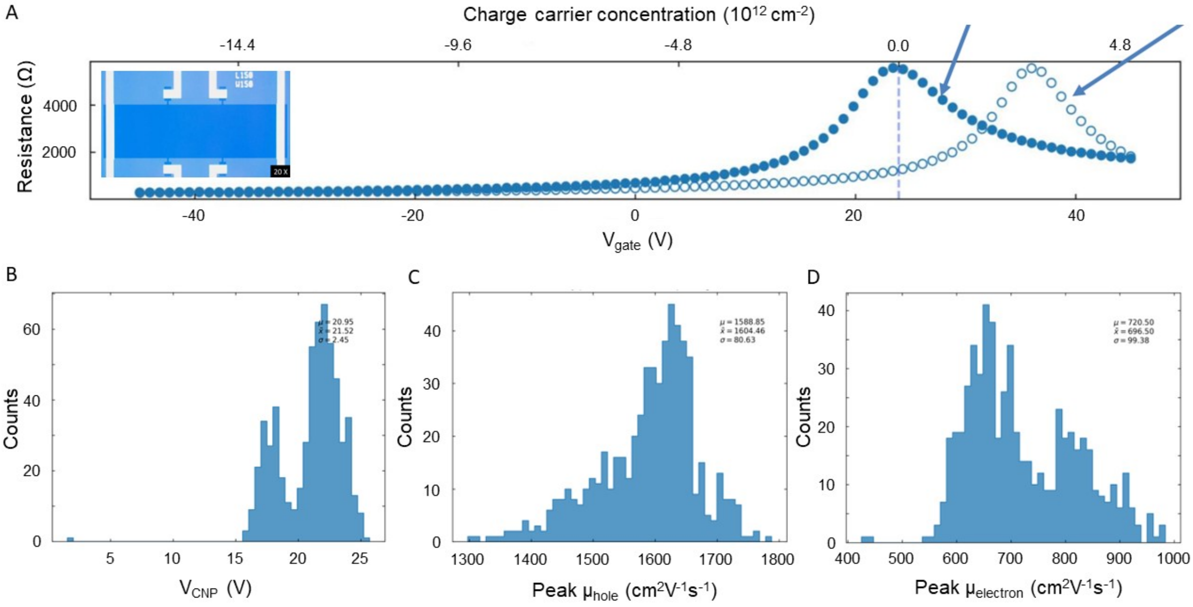


**Figure S15:** GFET characterization (4-terminal) of PYRENE wafer-scale device fabrication. A) Typical gate sweep for a GFET on a PYRENE wafer with 90 nm $SiO_2$ layer. Forward (backward) sweep in filled (empty) circles. B-D): $V_{CNP}$, $\mu_{hole}$, and $\mu_{electron}$ histograms for devices fabricated on a wafer with 90 nm $SiO_2$ thickness. Gate sweeps were performed at 10 V/s.

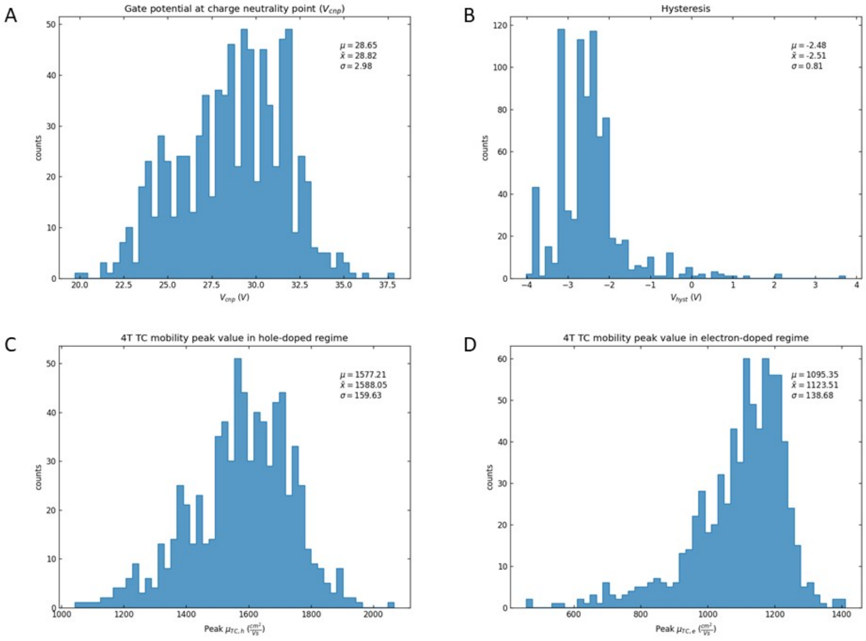


**Figure S16:** GFET characterization of BARE wafer-scale device fabrication. A-D): $V_{CNP}$, hysteresis, $\mu_{hole}$, and $\mu_{electron}$ histograms for devices fabricated on a wafer with 90 nm $SiO_2$ thickness. Gate sweeps were performed at 10 V/s.

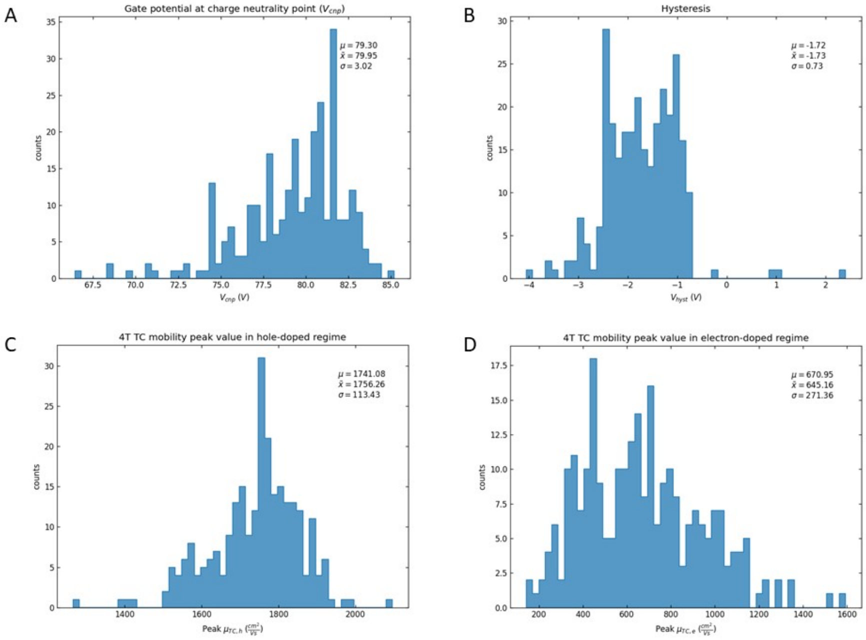


**Figure S17:** GFET characterization of BARE wafer-scale device fabrication. A-D): $V_{CNP}$, hysteresis, $\mu_{hole}$, and $\mu_{electron}$ histograms for devices fabricated on a wafer with 285 nm $SiO_2$ thickness. Gate sweeps were performed at 10 V/s.

**Table S6:** Statistical analysis for GFETS on BARE and PYRENE wafers. Average values are tabulated, standard deviation indicated in brackets.

| | Device fabrication | | Charge neutrality point (V) | | Carrier density ($x10^{12}$ $cm^{-2}$) | | Transconductance Mobility ($x10^3$ $cm^2V^{-1}s^{-1}$) | | | |
|---|---|---|---|---|---|---|---|---|---|---|
| | | | | | | | 2-terminal | | 4-terminal | |
| **Surface + oxide** | Count | Yield | $V_{CNP,up}$ | $V_{hyst}$ | $n_0$ | $n_{hyst}$ | $\mu_{hole}$ | $\mu_{electron}$ | $\mu_{hole}$ | $\mu_{electron}$ |
| BARE 285 nm | 830 | 31.4 | 79.3 (3.0) | -1.7 (0.7) | 6.00 (0.23) | -0.13 (0.06) | 1.76 (0.08) | 0.73 (0.31) | 1.74 (0.11) | 0.671 (0.27) |
| BARE 90 nm | 833 | 89.0 | 28.7 (3.0) | -2.5 (0.8) | 6.85 (0.71) | -0.59 (0.19) | 1.53 (0.15) | 1.08 (0.17) | 1.58 (0.16) | 1.10 (0.14) |
| PYRENE 285 nm | 325 | 86.2 | 74.8 (4.4) | -5.0 (1.4) | 5.66 (0.33) | -0.38 (0.1) | 2.10 (0.09) | 1.01 (0.15) | 1.99 (0.12) | 0.94 (0.14) |
| PYRENE 90 nm | 586 | 99.7 | 21.0 (2.5) | -12.8 (1.0) | 5.02 (0.58) | -3.06 (0.17) | 1.63 (0.05) | 0.76 (0.10) | 1.59 (0.08) | 0.72 (0.10) |
